# Secretory IgA amplification during immune checkpoint blockade enhances the control of tumor growth by enterotropic T cells

**DOI:** 10.1126/sciadv.aeb5308

**Published:** 2025-10-03

**Authors:** Benedetta De Ponte Conti, Rebecca Marino, Tanja Rezzonico-Jost, Mattia Forcato, Davide Mangani, Elisabetta Notario, Giorgio Gargari, Elena Carelli, Andrea Rinaldi, Andrea Raimondi, Simone Moro, Marinella Marzano, Grazia Visci, Lisa Perruzza, Matteo Raneri, Denise Dallavalle, Giacomo Mantegazza, Ludovica Montani, Francesco Prisco, Roshan Takur, Jens Geginat, Frauke Seehusen, Samuele Notarbartolo, Graziano Pesole, Silvio Bicciato, Simone Guglielmetti, Fabio Grassi

**Affiliations:** ^1^ Institute for Research in Biomedicine, Faculty of Biomedical Sciences, Università della Svizzera Italiana, Bellinzona 6500, Switzerland.; ^2^ Graduate School of Cellular and Molecular Sciences, University of Bern, Bern 3012, Switzerland.; ^3^ Department of Molecular Medicine, University of Padova, Padova 35121, Italy.; ^4^ Institute of Biomembranes, Bioenergetics and Molecular Biotechnologies, National Research Council, Bari 70126, Italy.; ^5^ Division of Food Microbiology and Bioprocesses, Department of Food, Environmental and Nutritional Sciences (DeFENS), University of Milan, Milan 20133, Italy.; ^6^ Istituto Nazionale Genetica Molecolare “Romeo ed Enrica Invernizzi”, Milan 20122, Italy.; ^7^ Institute of Oncology Research, Oncology Institute of Southern Switzerland, Bellinzona 6500, Switzerland.; ^8^ Department of Biosciences, Biotechnologies and Environment, University of Bari Aldo Moro, Bari 70126, Italy.; ^9^ Department of Biotechnology and Biosciences (BtBs), University of Milano-Bicocca, Milan 20126, Italy.; ^10^ Infectious Diseases Unit, Fondazione IRCCS Ca’ Granda Ospedale Maggiore Policlinico, Milan 20123, Italy.; ^11^ Laboratory for Animal Model Pathology, Institute of Veterinary Pathology, Vetsuisse Faculty, University of Zurich, Zurich 8057, Switzerland.; ^12^ Department of Clinical Sciences and Community Health, University of Milan, Milan 20129, Italy.; ^13^ Department of Medical Biotechnology and Translational Medicine, University of Milan, Milan 20129, Italy.

## Abstract

The gut microbiota is essential for many aspects of host physiology, and secretory immunoglobulin A (sIgA) modulates its function. The microbiota community determines the efficacy of immune checkpoint blockade (ICB) in cancer immunotherapy; however, mechanisms able to improve this function are not known. Extracellular adenosine triphosphate (ATP) released by the microbiota restricts the sIgA repertoire by limiting T follicular helper (T_FH_) cell activity in the Peyer’s patches via stimulation of the ionotropic P2X7 receptor. We show that sIgA amplification by oral administration of the ATP hydrolyzing enzyme apyrase corrects enteropathic features of ICB and improves therapeutic efficacy. Consistent with sIgA function in reshaping the gut ecosystem and enhancing ICB, *IgA^−/−^* mice did not show any improvement of antitumor response by apyrase administration. Mechanistically, data in mice and patients with cancer suggest that invigorated enterotropic cytotoxic T cells expressing the chemokine receptor CCR9 replenish the tumor microenvironment in a CCL25-mediated manner and control tumor growth, resulting in improved ICB efficacy.

## Teaser

Secretory IgA adapts the intestinal ecosystem to cancer immunotherapy and enhances tumor growth control by enterotropic T cells.

## INTRODUCTION

Cancer represents one of the most important causes of mortality in Western countries. In recent years, several therapeutic strategies have been successfully exploited to unleash the antitumor immune response and treat various forms of the disease (*1*). In solid tumors, immune checkpoint inhibitors (CPIs) (e.g., anti–PD-1/PD-L1 and anti–CTLA-4 antibodies) have provided unprecedented successes in eradicating previously incurable tumors in a fraction of responder subjects (*2*). However, the considerable number of patients nonresponding or developing resistance to the therapy and subjects showing severe immune-related adverse events (irAEs) associated with CPI administration has prompted the search for more effective and less toxic combinatorial therapeutic regimens able to overcome the unsuccessful or pathogenic application of immune checkpoint blockade (ICB).

The gut microbiota is essential for many aspects of host physiology, including intestinal and immune system differentiation, tissues homeostasis, and systemic metabolism (*3*). Deleterious alterations in the composition of the microbial community structure, referred to as dysbiosis, have been associated to a number of diseases (*4*–*6*). The intestinal ecosystem represents an essential cofactor in ICB outcome, and preclinical studies have pinpointed the specific beneficial function of selected microbes, such as *Bacteroides fragilis*, *Bifidobacterium*, *Akkermansia muciniphila*, and Ruminococcaceae (*7*–*12*). Accordingly, dysbiosis induced by antibiotics inhibited the clinical benefit of ICB in patients with advanced cancer (*13*), which could be restored by administration of different bacterial taxa; in this respect, particularly relevant was the association of improved tumoricidal activity with the accumulation of CD4^+^ T cells expressing the small intestine–associated chemokine receptor 9 (CCR9) and the T helper 1–associated chemokine receptor CXCR3 in mesenteric lymph nodes (mLNs), tumor-draining LNs, and tumor beds of mice gavaged with *A. muciniphila* after antibiotic treatment (*10*). Different microbes can condition the migration of T cells from the intestine to the tumor bed with opposite influence on ICB outcome (*14*, *15*). Beyond preclinical models, the most compelling evidence associating the intestinal microbiota with ICB outcome was the demonstration that fecal microbiota transplant from patients with advanced melanoma responding to ICB could promote the antitumor response in immunotherapy-refractory subjects (*16*–*18*).

Many factors contribute to the shaping of the gut microbiota, but specific mechanisms responsible for host microbiota mutualism are not thoroughly understood. Secretory immunoglobulin A (sIgA) may enhance commensal bacteria colonization by promoting adhesion and/or nutrient utilization of bacteria within the mucosal niche (*19*, *20*). IgA-coated bacteria contribute to host physiology and metabolism (*21*) and are important for the preservation of commensals diversity and community networks in the human gut (*22*). High-affinity T cell–dependent sIgA in the gut is mainly generated in gut-associated lymphoid tissue (GALT), such as Peyer’s patches (PPs), in the small intestine. We have previously shown that microbiota-derived extracellular adenosine triphosphate (eATP) can regulate sIgA production by limiting T follicular helper (T_FH_) cell abundance in the PPs of the terminal ileum through the stimulation of the ionotropic P2X7 receptor (P2X7R) (*23*). This signaling mechanism proved to be important in shaping a beneficial gut microbiota (*24*) and, conversely, limited the effective generation of protective sIgA upon oral vaccination with attenuated enteric pathogens (*25*). Apyrase is an ATP diphosphohydrolase that catalyzes the sequential hydrolysis of ATP to adenosine diphosphate (ADP) and ADP into adenosine monophosphate (AMP). The abrogation of intestinal eATP in gnotobiotic mice colonized with *Escherichia coli* transformants expressing the apyrase gene from *Shigella flexneri* (*E. coli^pApyr^*) resulted in sIgA repertoire amplification (*26*). In addition, the enhanced production of sIgA in mice lacking the P2X7R resulted in increased sIgA coating of bacteria typically residing in the small intestine, especially *Lactobacillus*, *Enterococcus*, and Enterobacteriaceae that conditioned systemic metabolism (*21*). Given the importance of the gut ecosystem in conferring responsiveness to ICB, we leveraged the function of apyrase on sIgA repertoire to enhance intestinal fitness during ICB. This approach proved to be effective in preventing small intestinal alterations associated to ICB, enhancing effector T cell migration from the gut to the tumor bed and boosting the tumoricidal effect of CPIs.

## RESULTS

### sIgA amplification and coating of commensal microbes improve ICB efficacy

sIgA has a pleiotropic function in adapting the gut microbiota community to a multitude of perturbations. Among many effects (*27*), it directly limits absorption of bacterial products (*23*), accelerates the small intestinal transit (*28*), and regulates topography of bacteria, thereby conditioning epithelial gene expression and nutrient absorption (*26*). In patients with IgA deficiency, the impairment of the mucosal IgA response leads to aberrant systemic exposure to commensal microbes and CD8 T cell dysfunction consistent with an exhaustion-like process (*29*). The quantification of IgA-coated bacteria and soluble IgA in the ileum of mice bearing subcutaneous MC38 colon adenocarcinoma and treated with anti–PD-L1 showed a negative correlation with tumor size, suggesting that sIgA abundance could contribute to the therapeutic efficacy of ICB (fig. S1, B and C).

**Fig. 1.**
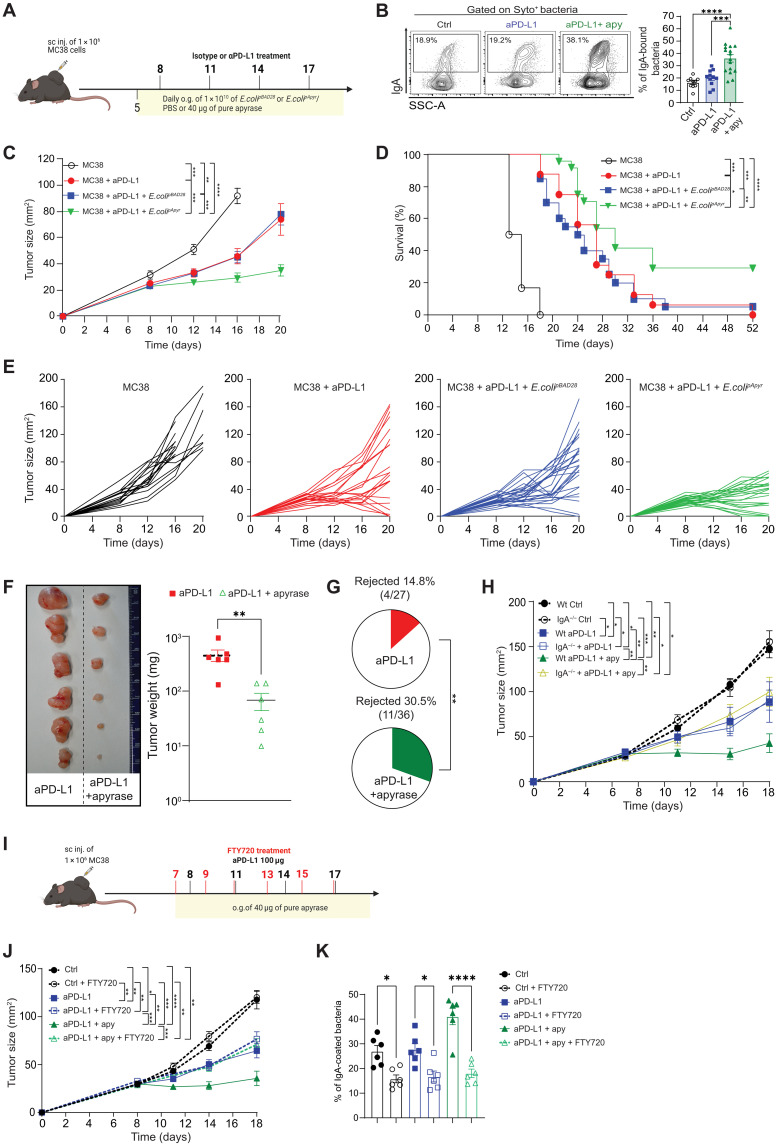
sIgA correlates with ICB efficacy. (**A**) Mice engrafted with MC38 tumor cells were daily gavaged from day 5 with 10^10^ CFU (colony-forming units) of *E. coli^pBAD28^* or *E. coli^pApyr^* or PBS or 40 μg of pure apyrase. On days 8, 11, 14, and 17, mice were intraperitoneally treated with 100 μg of isotype control or anti–PD-L1. sc, subcutaneous; o.g., oral gavage. (**B**) Flow cytometry and bar plots of IgA-coated bacteria in the ileum. (**C**) Tumor growth and (**D**) survival curves of mice treated as indicated (*n* = 20 to 30 mice per group). In (**E**), each line represents the tumor growth of a single mouse. Data are presented as means ± SEM from three pooled experiments. (**F** and **G**) Mice were treated with isotype or anti PD-L1 alone or in combination with 40 μg of pure apyrase. (F) Representative tumor images (left) and statistical analysis of tumor weights in the two groups (right). (G) Tumor rejection rate on day 18. The number of mice, which rejected the tumor over total mice, is indicated above the pie charts. Means ± SEM from four pooled experiments are shown. (**H**) Tumor growth in WT or IgA^−/−^ mice treated with isotype or anti PD-L1 combined with 40 μg of pure apyrase. Data are from two pooled experiments (*n* = 5 to 13 mice per group). (**I**) Treatment schedule: Starting from the day before anti–PD-L1 treatment, mice were orally treated with either PBS or 40 μg of pure apyrase; DMSO (dimethyl sulfoxide) or FTY720 (1 mg/kg) was administered at the indicated time points. (**J**) Tumor growth and (**K**) bar plots of IgA-coated bacteria in the ileum of mice treated as indicated in (I) (*n* = 10 to 18 mice per group). Data are presented as means ± SEM. The Spearman correlation for linear correlation, two-way ANOVA for tumor growth, Mantel-Cox log-rank test for survival curves, and two-tailed Mann-Whitney *U* test for tumor weight were performed. *P < 0.05; **P < 0.01; ***P < 0.001; ****P < 0.0001.

Microbiota-derived eATP limits sIgA production via P2X7R-mediated inhibition of T_FH_ cells in PPs (*23*). Therefore, we tested whether reducing eATP concentration in the terminal ileum by orogastric administration of *E. coli^pApyr^* resulted in enhanced control of tumor growth by anti–PD-L1. Quantification of ileal eATP upon administration of anti–PD-L1 combined with *E. coli^pApyr^* or *E. coli* transformants carrying an empty vector (*E. coli^pBAD28^*) (*30*) showed that combining *E. coli^pBAD28^* to anti–PD-L1 did not cause significant changes in eATP concentration as compared to mice treated with standalone *E. coli^pBAD28^*; however, the combination of anti–PD-L1 with *E. coli^pApyr^* resulted in a significant decrease in eATP concentration in the ileum and a significant increase in the percentage of IgA-coated bacteria as compared to the other groups of mice (fig. S1, D and E). Moreover, the frequency of IgA-bound bacteria was inversely correlated with ileal eATP concentration (fig. S1F). We grafted subcutaneously either MC38 cells or the B16-OVA melanoma cell line into C57BL/6 mice and starting from day 5, when tumors were measurable, we administered to mice *E. coli^pApyr^* or *E. coli^pBAD28^* in combination with intraperitoneal administration of anti–PD-L1 from day 8 after tumor engraftment (Fig. 1A). As expected, we observed enhanced sIgA coating of ileal bacteria in mice treated with apyrase (Fig. 1B), as well as an increase in soluble IgA concentration (fig. S1A). Notably, in both tumor models, *E. coli^pApyr^* administration significantly improved anti–PD-L1–dependent control of tumor growth (Fig. 1, C and E, and fig. S1H) and mice survival (Fig. 1D and fig. S1I), compared to mice receiving anti–PD-L1 alone or in combination with *E. coli^pBAD28^*. Analogous results were obtained by oral administration of purified recombinant apyrase, showing that the efficacy of the treatment did not depend on the delivery of apyrase by the bacterial vector (Fig. 1F and fig. S1G). Tumor eradication in mice treated with the enzyme combined with anti–PD-L1 doubled with respect to standalone CPI (Fig. 1G). As previously described, mice that rejected the tumor were resistant to subsequent tumor engraftment, suggesting that these mice developed tumor-specific memory (*31*). Administration of apyrase in the absence of CPI did not result in enhanced control of tumor growth with respect to untreated mice, suggesting that it exerted an adjuvant function on the antitumor response induced by the CPI (fig. S2A). To exclude that the effect of apyrase could be due to reduced eATP levels in the tumor microenvironment (TME), we injected *E. coli^pApyr^* into the tumor of mice treated with anti–PD-L1. However, we did not observe any variation in tumor growth with respect to mice injected with *E. coli^pBAD28^* (fig. S2B). We checked that gavaging of *E. coli^pApyr^* did not affect eATP concentration in the TME by the analysis of tumor luminescence in mice engrafted with B16 cells expressing plasma membrane–targeted luciferase (B16-pmeLUC) (*32*). Figure S2C shows that oral *E. coli^pApyr^* did not result in reduction of eATP in the TME, whereas a reduction was observed in mice in which *E. coli^pApyr^* was injected intratumorally.

To ascertain whether this effect was limited to anti–PD-L1 treatment, we tested different CPIs in combination with *E. coli^pApyr^* or recombinant apyrase. We found that apyrase could improve the efficacy of ICB in mice treated with standalone anti–CTLA-4 or in combination with anti–PD-L1 (fig. S3, A to D), as well as in mice bearing poorly immunogenic Lewis lung carcinoma (LLC) and treated with anti–PD-L1 combined with agonist anti-CD40 antibodies (fig. S3, E and F). These data suggest that apyrase could convert immunotherapy-resistant tumors to CPI responsiveness. To prove that apyrase effect was not restricted to C57BL/6 genetic background, we engrafted CT26 colon carcinoma in BALB/c mice, which are known to display a different microbiota and IgA polyreactivity with respect to C57BL/6 mice (*33*), and treated them as MC38 tumor-bearing mice (Fig. 1A). As observed in C57BL/6 mice, apyrase treatment promoted the enhancement of the antitumor response induced by anti–PD-L1 (fig. S3, G and H). Together, these data suggest that ileal eATP reduction by oral apyrase could be beneficial in different immunotherapeutic strategies.

To assess whether the antitumor activity of apyrase was dependent on sIgA, we engrafted wild-type (WT) and *IgA*^−/−^ C57BL/6 mice with MC38 cells and administered anti–PD-L1 in combination with apyrase. As shown in Fig. 1H, the beneficial effect of apyrase in the control of tumor growth by anti–PD-L1 was lost in *IgA*^−/−^ mice, whereas the effect of anti–PD-L1 was not influenced by the lack of IgA. Therefore, the efficacy of ICB seems to benefit from sIgA amplification whereas immune dysregulation of *IgA*^−/−^ mice did not affect the immune stimulating properties of CPI. To exclude the influence of systemic effects resulting from congenital IgA deficiency on the outcome of apyrase addition to the CPI, we took advantage of fingolimod (FTY720), a functional antagonist of the S1P1 receptor, which blocks lymphocyte egress from secondary lymphoid organs (Fig. 1I). Tumor control induced by standalone anti–PD-L1 is not influenced by FTY720 because it is predominantly mediated by T cells already present in the TME (*34*). However, fingolimod administration to WT mice concomitant to apyrase abrogated the improvement of ICB efficacy (Fig. 1J). This effect was associated to the significant reduction of IgA coating of the microbiota induced by apyrase due to blockade of IgA^+^ plasmablasts egress from PPs (*35*) (Fig. 1K). These data together with the lack of effect of apyrase in *IgA*^−/−^ mice suggest that sIgA amplification by apyrase during ICB is a prerequisite for enhanced control of tumor growth.

### sIgA amplification by apyrase corrects the enteropathic response to ICB

**Fig. 2.**
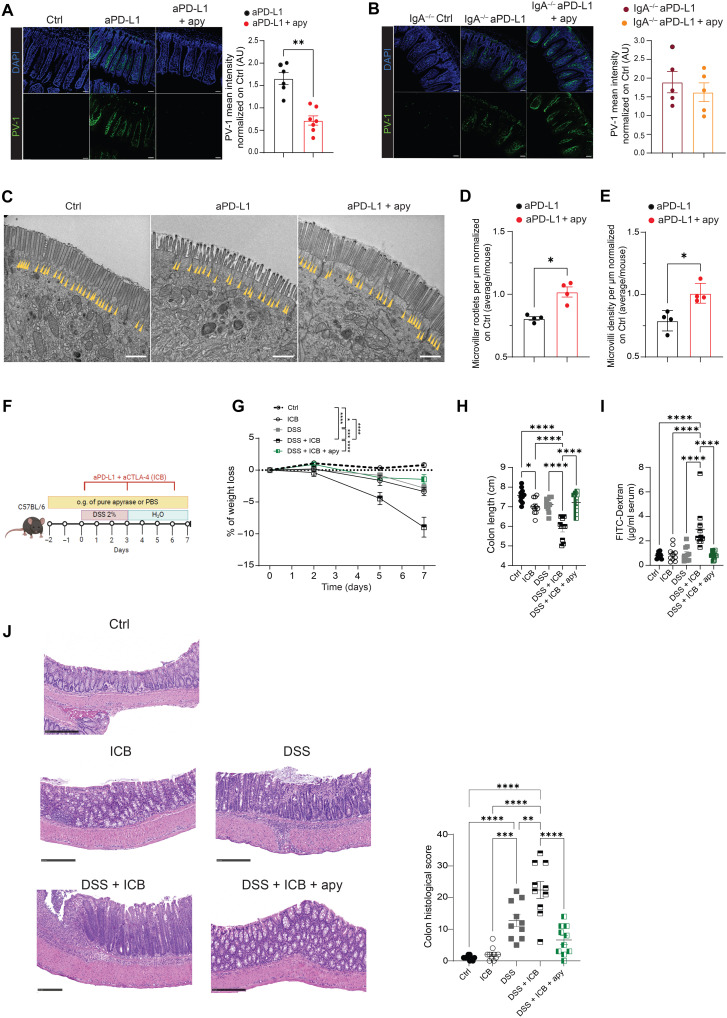
sIgA amplification by apyrase corrects ICB-mediated intestinal alterations. (**A** and **B**) Immunostaining of PV-1 (green) in ileal villi of C57BL/6 WT (A) or IgA^−/−^ (B) MC38 tumor-bearing mice that were untreated (Ctrl, *n* = 4), treated with standalone anti–PD-L1, or combined with apyrase. Nuclei were stained with DAPI (blue). Representative images of four to seven mice per group from two pooled experiments. Scale bars, 50 μm. PV-1 intensity in individual mice was normalized to controls. Data points represent single mice; means ± SEM. AU, arbitrary units. **P < 0.01 by the Mann-Whitney *U* test. (**C** to **E**) TEM images of enterocytes captured from the upper portion of villi from MC38 tumor-bearing mice that were untreated (Ctrl, *n* = 4), treated with standalone anti–PD-L1, or combined with apyrase. Yellow arrowheads designate microvilli rootlets. TEM images representative of four mice per group. Scale bars, 1 μm (C). Graphs of average rootlets (D) and microvilli (E) density quantified from TEM images are displayed as means ± SEM. Data points represent single mice. *P < 0.05 by the Mann-Whitney *U* test. (**F** to **J**) Murine model of irAEs. (F) Schematic of the irAE model. Mice were given 2% DSS in drinking water for 3 days, followed by 4 days of water. Starting 2 days before DSS, a group received daily gavage of 40 μg of apyrase. On days 0, 3, and 6, mice received intraperitoneally either 100 μg of anti–CTLA-4 or 100 μg of anti–PD-L1 or IgG. Body weight loss over time (G), colon length (H), and dextran-FITC serum analysis (I) at the end of the experiment (*n* = 10 per group). (J) Representative H&E sections of colonic sample and statistical analysis of histopathological scores. Scale bars, 250 μm. Data points represent single mice. Error bars represent SEM. One-way ANOVA with Tukey’s multiple comparison test was used. *P < 0.05; **P < 0.01; ***P < 0.001; ****P < 0.0001.

ICB can result in severe gastrointestinal irAEs (*36*, *37*). In a phase 1 clinical trial, responder patients that benefited from fecal microbiota transplantation (FMT) displayed improved epithelial integrity and decreased gut permeability (*18*). The analysis of plasmalemma vesicle–associated protein-1 (PV-1), a marker of altered vascular gut permeability (*38*, *39*), in small intestinal villi of tumor-bearing mice by confocal microscopy showed the increased PV-1 expression in the ileum of anti–PD-L1–treated mice, suggesting that intestinal integrity was compromised by ICB (*8*); the combination of anti–PD-L1 with apyrase prevented the increase in PV-1 expression (Fig. 2A). Conversely, when apyrase was combined with anti–PD-L1 in tumor-bearing *IgA^−/−^* mice, we no longer observed the beneficial reduction of the intensity of PV-1 staining in ileal villi (Fig. 2B). The brush border membrane is organized into apical F-actin–supported protrusions called microvilli that increase the surface area and functional capacity of enterocytes lining the intestinal tract. The reduction of microvilli and microvilli rootlets is a feature characterizing the small intestinal pathology of Crohn’s disease (*40*). Transmission electron microscopy (TEM) of the ileum of tumor-bearing mice treated with anti–PD-L1 showed the marked reduction of the density of microvilli and their rootlets. By contrast, apyrase supplementation significantly prevented these structural abnormalities in ileal enterocytes (Fig. 2, C to E). Again, consistent with the role of sIgA in mediating protection from ICB-induced intestinal damage by apyrase, these lesions were not prevented by apyrase administration in *IgA^−/−^* mice (fig. S4, A to C). To further investigate the impact of apyrase on intestinal “fitness” during ICB, we performed RNA sequencing (RNA-seq) on intestinal epithelial cells (IECs) from mice treated with standalone anti–PD-L1 or anti–PD-L1 combined with apyrase. In apyrase-treated mice, several genes involved in gut barrier integrity and immunomodulation (*Ido1*, *Cldn4*, *Adm*, *Atoh1*, *Muc2*, *Lama3*, *Itgav*, *Egfr*, and *Lpn2*) (*41*–*48*) were significantly up-regulated (fig. S4D and table S1). Moreover, the administration of apyrase prevented the up-regulation of genes involved in the cellular response to stress (fig. S4E and table S2). Together, these data suggest that apyrase-mediated sIgA amplification could prevent ileal epithelium damage induced by ICB within the treatment of solid tumors.

Because ICB does not induce an aggressive enteropathy in mice, to address the possible efficacy of apyrase in improving intestinal function in severe gastrointestinal irAEs, we exploited the experimental layout described by Zhou *et al.* (*49*), in which the combined administration of CPIs with low-dose dextran sulfate sodium (DSS) results in the intestinal irAE that characterizes ICB failure and threatens the life of patients treated with CPIs. We recapitulated that low-dose DSS synergizes with CPIs (anti–CTLA-4 and anti–PD-L1) in leading to a significant reduction in body weight and colon length, enhanced intestinal permeability, and histopathological traits in the colon. Notably, apyrase supplementation restored these features induced by the combination of the two CPIs with DSS, suggesting that it could prevent the intestinal irAEs induced by immunotherapy in patients with tumor (Fig. 2, F to J).

### Increased effector functions in TILs by apyrase administration

**Fig. 3.**
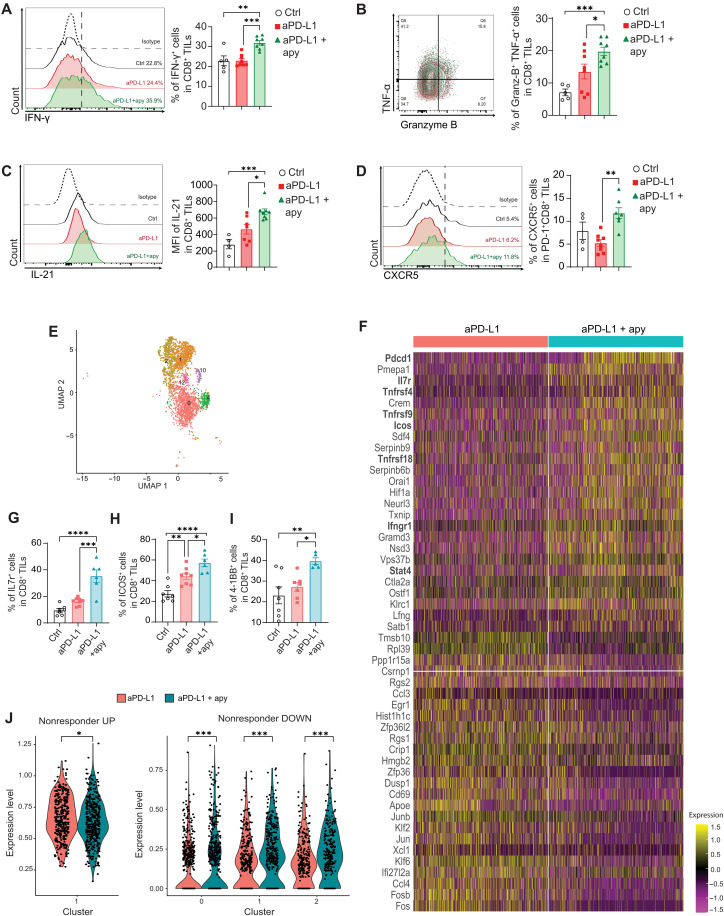
Increase in effector functions in CD8 TILs by apyrase administration. (**A** to **D**) Representative flow cytometry histograms and bar plots of CD8^+^ TIL subpopulation recovered from the tumor of mice on day 12 of the schedule shown in Fig. 1A (24 hours after the 2nd dose of anti–PD-L1). Cells were selected as Zombie^−^, CD45^+^, and CD8^+^TCRβ^+^ and analyzed on the gated lymphocyte population. Histograms and bar graphs reflect the proportion of IFN-γ^+^ (A), TNF-α^+^Granzyme B^+^ (B), and CXCR5^+^ (D) and the mean fluorescence intensity (MFI) of IL-21 (C) in the indicated subsets of CD8 cells. The boundaries to quantify positive cells with the different staining were established using isotype-matched negative antibodies (dotted line). Data points represent single mice. Error bars represent SEM. One-way ANOVA with Tukey’s multiple comparison test was used. (**E**) Uniform manifold approximation and projection (UMAP) visualization of CD8 T cells infiltrating tumors treated with either anti–PD-L1 plus *E. coli^pBAD28^* or anti–PD-L1 plus *E. coli^pApyr^*. Cells are colored according to cluster identity. The clusters in the UMAP are coming from pooled mice from the two differently treated groups. (**F**) Gene expression heatmap featuring the top 25 up-regulated and down-regulated differentially expressed genes between CD8 T cells clusters in tumors treated with anti–PD-L1 plus *E. coli^pBAD28^* versus anti–PD-L1 plus *E. coli^pApyr^* (*n* = 3/4 mice per group). (**G** to **I**) Statistical analysis of IL-7R (G), ICOS (H), and 4-1BB (I) CD8 TILs recovered from tumors treated as detailed in Fig. 1A on day 18. Data points represent single mice. Error bars represent SEM. One-way ANOVA with Tukey’s multiple comparison test was used. (**J**) Violin plots showing the expression of the ICB nonresponder gene signatures [up-regulated and down-regulated genes in patients not responding to ICB from ref. (*55*)] in clusters of CD8 T cells. The Mann-Whitney test with Benjamini and Hochberg correction was used. *P < 0.05; **P < 0.01; ***P < 0.001; ****P < 0.0001.

Then, we aimed at characterizing possible functional and phenotypic differences of tumor-infiltrating lymphocytes (TILs) induced by combining apyrase to anti–PD-L1 in the treatment of tumor-bearing mice. To avoid scoring differences that were due to different tumor masses, we analyzed TILs after the second anti–PD-L1 injection when differences in tumor size between the various groups of treatment were not significant. In mice bearing B16-OVA tumors, tetramer staining revealed a significant increase in the number of OVA-specific CD8^+^ T cells in the group treated with anti–PD-L1 and apyrase with respect to mice treated with vehicle or standalone anti–PD-L1 (fig. S5A), consistent with increased infiltration of tumor antigen–specific CD8 T cells. We addressed the antitumor proficiency of TILs infiltrating MC38 tumors and found significantly increased secretion of interferon-γ (IFN-γ) (Fig. 3A), increased proportion of TNF-α^+^Granzyme B^+^ cells (Fig. 3B) in CD8^+^ TILs, as well as IFN-γ and tumor necrosis factor–α (TNF-α) secreting CD4^+^ TILs in anti–PD-L1/apyrase-treated mice (fig. S5, B and C). Interleukin-21 (IL-21) was shown to improve T cell–mediated antitumor immunity within ICB (*50*); notably, we found enhanced expression of IL-21 in both CD8^+^ (Fig. 3C) and CD4^+^ (fig. S5D) TILs by apyrase administration. In addition, we detected a significant increase in CD8^+^PD-1^+^CXCR5^+^ T cells (Fig. 3D), a cell subset associated with the proliferative burst induced by PD-1 blockade (*51*). The same functional improvement of CD8^+^ TILs was also observed in mice bearing B16-OVA (fig. S5, F to H) and LLC (fig. S5, I to L) tumors by combining apyrase to anti–PD-L1 and anti–PD-L1/anti-CD40, respectively. These phenotypic changes in TILs were not observed in mice solely treated with apyrase (fig. S5, M to Q), suggesting that they resulted from the synergy of the enzyme in the intestine with ICB.

To gain further insights into the underlying biology, we performed single-cell RNA sequencing (scRNA-seq) of the CD45^+^ cells infiltrating tumors treated with either anti–PD-L1 or anti–PD-L1 plus apyrase. On the basis of CD3 gene expression, we reclustered and annotated the T cells by checking the expression of several markers related to T cell biology (fig. S6, A to C). The *Cd8a* gene characterized eight clusters (C0, C1, C2, C4, C5, C7, C10, and C12), whereas the *Cd4* gene only two clusters [C3 regulatory T (T_reg_) and C8 conventional T (T_conv_) cells]. We also annotated a γδ T cell receptor (TCRγδ) T cell cluster (C6), a shared cluster between αβ T cell receptor (TCRαβ) CD4 and CD8 encompassing naïve-like cells (C9), and a cluster expressing high levels of CD14 and most likely being a contaminant of myeloid origin (C11). This added resolution allowed us to focus our attention on clusters characterized by *Cd8a* gene expression as our preliminary data suggest that they play a major role in mediating the effects induced by apyrase combination (Fig. 3E). C5 and C7 were excluded from further analysis as the former showed expression of markers associated with CD8-expressing nonconventional T cells such as γδT and natural killer (NK) cells (*Klra1*, *Klrb1c*, *Tcrg-c4*, and *Cd160*), whereas the latter showed signs of low-quality capture and sequencing (low transcript content, mainly of mitochondrial origin) (fig. S6B). We did not detect significant differences in the distribution of CD8 clusters in mice treated with standalone anti–PD-L1 versus anti–PD-L1 combined with apyrase (fig. S6D). The overall comparison of the transcriptome of CD8 T cell clusters showed a significant increase in gene transcripts associated with an active and functional antitumor immune response, including *Pdcd1*, *Il7r*, *Icos*, *Tnfrsf4*, *Tnfrsf9*, *Tnfrsf18*, *Ifngr1*, and *Stat4* in the ICB plus apyrase group (Fig. 3F), which was confirmed by flow cytometry analysis for IL-7R, ICOS, and 4-1BB (Fig. 3, G to I). To annotate the remaining CD8 T cell clusters beyond the mere expression of few markers, we ran a series of transcriptional signatures known to characterize diverse CD8 T cell states. Specifically, we tried to pinpoint the main clusters that follow the CD8 T cell trajectory within tumors. This analysis revealed that C1 displayed the highest score for a T precursor exhausted (T_pex_) signature (*52*) (fig. S6E). C2 and C12 showed the highest score for CD8 dysfunctional cells (*53*), with C12 being the transitory effector–like population (T_eff_-like), still retaining some proliferative potential, whereas C2 being the terminally exhausted (T_ex_) pool (*54*) (fig. S6F). C0 most likely resembled T effector memory (T_em_) cells (*Ccl5*, *Gzmk*, *Gzma*, *Nkg7*, *Cxcr3*, *Ifgr1*, and *Klf2*), whereas C10 appeared to feature tissue-resident memory T (T_rm_) cells (*Pgk1*, *Jun*, *Bnip3*, and *Tnfsf9I*) (fig. S6G). In an effort to associate the transcriptional changes happening within the tumor-infiltrating CD8 T cells with data of human relevance, we further projected the ICB nonresponder signature (*55*) in the various CD8 T cell clusters by comparing differently treated mice. Genes up-regulated in patients not responding to ICB were more represented in CD8 T cells from mice treated with anti–PD-L1 combined with control *E. coli* transformants, whereas genes down-regulated in nonresponders scored higher in the T_em_, T_pex_, and T_ex_ (C0, C1, and C2) infiltrating tumors in mice where anti–PD-L1 was associated with apyrase releasing *E. coli* transformants (Fig. 3J). Together, these data suggest that oral apyrase in combination with ICB could be exploited to improve the antitumor proficiency of cytotoxic T cells and obtain a better control of tumor growth.

### Enrichment of ileal *Lactobacillus johnsonii* characterizes apyrase-mediated enhancement of ICB efficacy

**Fig. 4.**
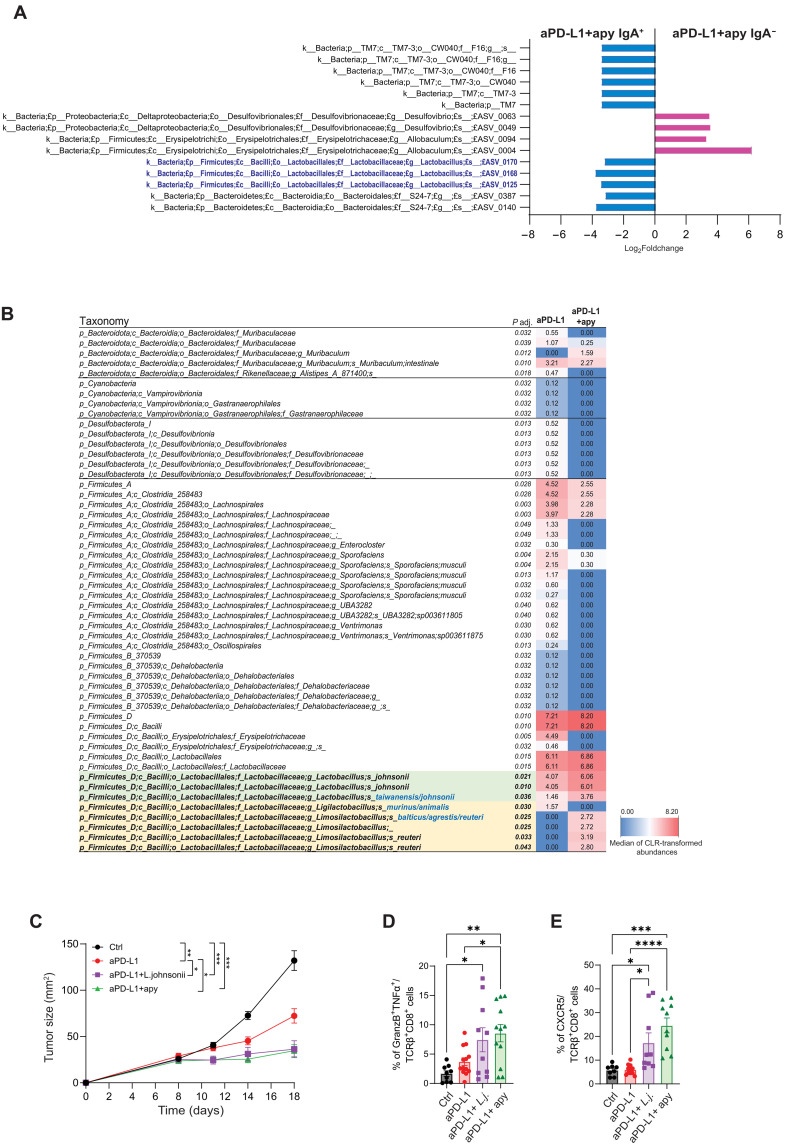
Enrichment of ileal *Lactobacilli* characterizes apyrase-mediated enhancement of ICB efficacy. (**A**) Differential abundance analysis performed with DESeq2 comparing microbial taxa in IgA^+^ versus IgA^−^ ileal fractions from mice treated with anti–PD-L1 and apyrase (*n* = 7). Log₂ fold changes of significantly different bacterial taxa, as determined by Wald tests followed by Hochberg correction for multiple testing. Taxa belonging to the genus *Lactobacillus* are highlighted in blue. All taxa shown met the statistical significance threshold after correction (*P* adj. < 0.05). The same analysis performed in mice treated with standalone anti–PD-L1 did not reveal any significantly different abundance of bacterial taxa between the IgA^+^ and IgA^−^ fraction. (**B**) Significantly different taxa determined through the Mann-Whitney test carried out with Centered Log-Ratio (CLR)-transformed bacterial abundances. The blue-white-red heatmap represents the median CLR-transformed abundances of the reported taxonomic units. Taxonomic names written in blue were determined through a manual BLASTN search in GenBank using the sequence of the corresponding ASV. The bacterial community structure of the ileum was analyzed in anti–PD-L1–treated mice with (*n* = 8) or without (*n* = 8) the administration of recombinant apyrase. (**C** to **E**) On day 0, mice were subcutaneously injected with 1 × 10^6^ MC38 tumor cells and treated with isotype or anti–PD-L1 and 40 μg of pure apyrase or 10^9^ *L. johnsonii* or PBS. (C) Tumor growth of mice treated as indicated. Means ± SEM from two experiments (*n* = 8 to 15 mice per group). [(D) and (E)] Bar plots of CD8^+^ TIL subpopulations. Cells were selected as Zombie^−^, CD45^+^, and CD8^+^TCRβ^+^. Proportion of Granzyme B^+^TNF-α^+^ (D) and CXCR5^+^ (E) CD8 TILs. The boundaries to quantify positive cells with the different staining were established using isotype-matched negative antibodies. Data points represent single mice (*n* = 10 to 12). ANOVA for tumor growth and one-way ANOVA with Tukey’s multiple comparison test were used. *P < 0.05; **P < 0.01; ***P < 0.001.

sIgA contributes to the topographical distribution of the microbiota and controls the translocation of pathobionts into the lamina propria (*56*). Several studies have shown the targeting of selected bacterial species by sIgA under physiological and pathological conditions (*57*). To define commensal microbes targeted by sIgA during apyrase administration, we sorted IgA-coated ileal bacteria by fluorescence-activated cell sorting (FACS) and performed 16*S* ribosomal RNA (rRNA) gene sequencing. Bacteria isolated from *IgA*^−/−^ mice were used as negative controls for sorting (fig. S7C); to identify bacteria that were conditioned by sIgA coating in differently treated groups, we calculated the “IgA Index” or “Enrichment Score” of the different taxa for every single mouse by comparing the abundance in the IgA^+^ fraction to the IgA^−^ and unsorted fractions. Three amplicon sequence variants (ASVs) belonging to the genus *Lactobacillus* were significantly enriched in the IgA^+^ with respect to the IgA^−^ fraction in the group of mice treated with anti–PD-L1 and apyrase, whereas taxa belonging to families Erysipelotrichaceae and Desulfovibrionaceae were depleted in the IgA^+^ fraction. Conversely, no bacteria with a significant differential abundance in the IgA^+^ versus IgA^−^ fraction were identified in the group of mice treated with standalone anti–PD-L1 (Fig. 4A). This result suggests that the increased presence of *Lactobacilli* in the IgA^+^ sorted fraction within the combination treatment group was due to specific IgA coating and not merely a reflection of their overall gut abundance. Further bacterial taxa with a significant IgA index were identified by comparing the IgA^+^ to the unsorted fraction (fig. S7, A and B, and table S3). The enrichment of *Lactobacilli* in the IgA^+^ fraction of apyrase-treated mice was reminiscent of previous data obtained in mice with deletion of the *P2rx7* gene and enhanced sIgA coating of *Lactobacilli* (*21*). We performed a deeper metagenomic analysis of the microbiome by sequencing the full-length 16*S* rRNA gene using PacBio SMRTbell sequencing of bacteria residing in the ileum of mice treated with standalone anti–PD-L1 or anti–PD-L1 combined with apyrase. Relative abundance analysis of differentially represented ASVs revealed the selective enrichment of *L. johnsonii* and *L. reuteri* by apyrase administration. Conversely, Lachnospiraceae-, Erysipelotrichaceae-, and Desulfovibrionaceae-related taxa were reduced (Fig. 4B). The analysis of the ileal content of differently treated mice by quantitative polymerase chain reaction (qPCR) with primers specific for *L. johnsonii* showed the absolute increase in the abundance of this species in mice treated with anti–PD-L1 combined with apyrase (fig. S7D). β-Diversity by principal coordinate analysis (PCoA) derived from unweighted and weighted UniFrac showed that *L. johnsonii* was the bacterial species that most induced the segregation of mice treated with anti–PD-L1 combined with apyrase, whereas *Dubosiella newyorkensis* belonging to the family Erysipelotrichaceae promoted the variance of anti–PD-L1 mice (fig. S7E).

In mice, the abundance of *L. johnsonii* (*8*, *58*) or oral consumption of an exopolysaccharide from *L. delbrueckii* (*59*), which belongs to the same phylogroup, positively correlated with CD8 T cell antitumor cytotoxicity during ICB, suggesting that these microbes could have an adjuvant effect on ICB. To test the functional relevance of *Lactobacilli* enriched in mice treated with apyrase and anti–PD-L1, we isolated *L. gasseri/johnsonii*, *Limosilactobacillus balticus/agrestis/reuteri* and *Ligilactobacillus murinus/animalis* from this group of mice. To discriminate the importance of the different phylogroups in promoting the observed enhancement of the response to ICB, we checked the sensitivity to vancomycin of the different isolates. We found that *Limosilactobacillus* and *Ligilactobacillus* were resistant to the antibiotic [minimum inhibitory concentration (MIC) > 512 μg/ml], whereas *L. gasseri/johnsonii* was sensitive (MIC < 2 μg/ml), as previously shown (*60*). Sequencing of ileal bacteria from tumor-bearing mice that were either treated or not with vancomycin, followed by anti–PD-L1 combined with apyrase, confirmed the selective elimination of *L. johnsonii* by the antibiotic treatment (fig. S8). Therefore, to address whether the apyrase-mediated therapeutic improvement of ICB could be conditioned by vancomycin-sensitive *L. johnsonii*, we administered vancomycin to mice before MC38 tumor engraftment and kept mice under antibiotic treatment for the whole duration of the experiment. This scheme of vancomycin administration did not affect the antitumor response induced by anti–PD-L1; however, it completely abolished the enhancement of tumor growth control provided by the addition of apyrase to anti–PD-L1 (fig. S9A). The effect of vancomycin was associated with the abrogation of the increase in CD8^+^ICOS^+^ and IFN-γ secreting CD8^+^ TILs (fig. S9, B and C). To directly demonstrate the function of *L. johnsonii* in conditioning the intestinal ecosystem and promoting the tumoricidal effect of apyrase, we daily gavaged mice with *L. johnsonii* isolated from the ileum of apyrase-treated mice as a substitute of apyrase. The treatment of mice with anti–PD-L1 combined with bacteria provided analogous control of tumor growth as the combination of the antibody with apyrase (Fig. 4C) as well as a similar increase in TNF-α^+^Granzyme B^+^ and CXCR5^+^ CD8 TILs (Fig. 4, D and E). The enhancement of anti–PD-L1 efficacy mediated by *L. johnsonii* was dependent on sIgA because it was not observed in *IgA*^−/−^ mice (fig. S9D). Together, these data suggest that an apyrase-mediated increase in small intestinal colonization by *L. johnsonii*– and *L. johnsonii*–specific sIgA amplification promotes the beneficial influence of these bacteria on the tumoricidal activity of enterotropic T cells.

### Apyrase conditioning during ICB invigorates gut-resident CD8 T cells and promotes migration of enterotropic T cells to the TME

**Fig. 5.**
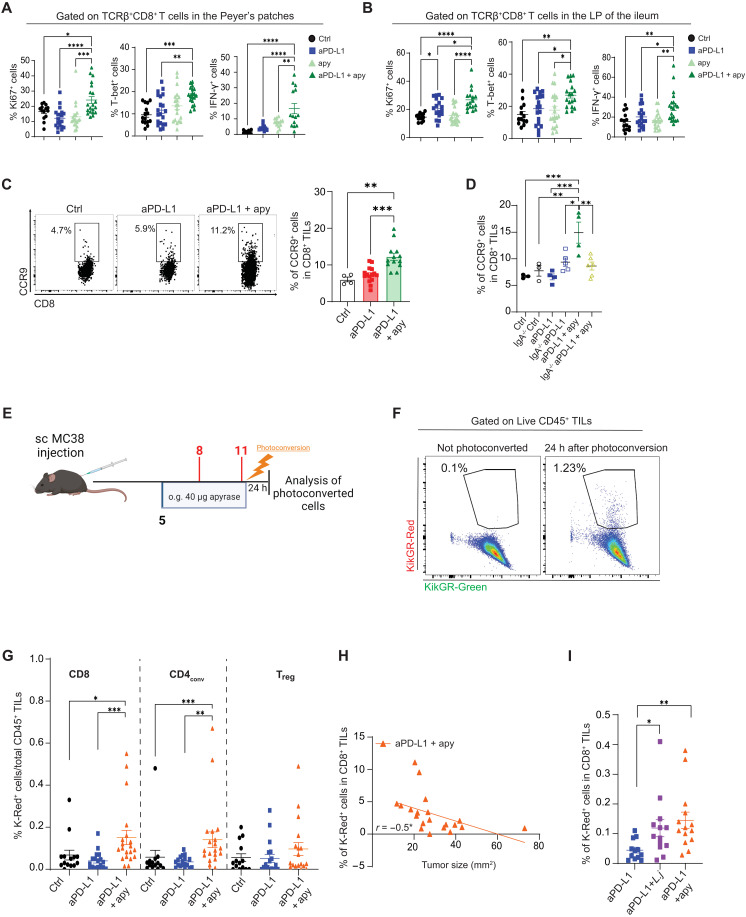
Apyrase conditioning of ICB invigorates gut-resident CD8 T cells and promotes their migration in the TME. (**A** and **B**) Statistical analysis of Ki-67, T-bet, and IFN-γ–expressing CD8 T cells recovered from PPs (A) and lamina propria (B) on day 12 (24 hours after the second dose of anti–PD-L1) of mice treated as detailed in Fig. 1A. Data points represent single mice from three experiments. (**C** and **D**) Representative histograms and bar graphs of CCR9^+^CD8^+^ TILs in tumors recovered from C57BL/6 WT (C) or C57BL/6 WT and IgA^−/−^ (D) mice on day 18 (24 hours after the fourth dose of anti–PD-L1). Cells were selected as Zombie^−^, CD45^+^, and CD8^+^TCRβ^+^. Data points represent single mice from two experiments. Error bars represent SEM. One-way ANOVA with Tukey’s multiple comparison. (**E**) Schematic of the experimental setup of KikGR mice engrafted subcutaneously with MC38 cells. The first three PPs of the ileum starting from the cecum were photoconverted on day 11 after the second shot of anti–PD-L1, and organs were collected 24 hours after photoconversion. h, hours. (**F**) Representative dot plot of TILs from tumor 24 hours after photoconversion or recovered from a nonphotoconverted mouse. (**G**) Percentage of K-Red CD8^+^, CD4^+^CD25^neg^ T_conv_ and T_reg_ cells within total CD45^+^ cells in the TME 24 hours postphotoconversion. Data points represent single mice from three experiments. (**H**) Correlation between K-Red CD8^+^ T cells in the TME and tumor size 24 hours after photoconversion (day 12). (**I**) Percentage of K-Red CD8^+^ within total CD45^+^ cells in the TME 24 hours postphotoconversion in mice treated with anti–PD-L1 alone or in combination with oral gavage of apyrase or *L. johnsonii*. Data points represent single mice. Error bars represent SEM. One-way ANOVA with Tukey’s multiple comparison test and Spearman correlation for linear correlation were used. *P < 0.05; **P < 0.01; ***P < 0.001.

In CD8 T cells isolated from PPs and ileal lamina propria of mice treated with anti–PD-L1 combined with apyrase, we detected enhanced staining in flow cytometry for the nuclear proliferation marker Ki-67 and T-box transcription factor T-bet, as well as increased IFN-γ secretion upon in vitro stimulation, all features associated with improved effector potential (Fig. 5, A and B). This apyrase-induced functional improvement of intestinal CD8 T cells was not observed in mice lacking IgA (fig. S10A). The chemokine receptor CCR9 characterizes enterotropic T cells. In patients with melanoma and mice with spontaneous melanoma, higher frequencies of peripheral blood CD8^+^CCR9^+^ T cells correlated with prolonged overall survival (*61*). We observed a significant increase in CCR9^+^ cells among both CD8^+^ (Fig. 5C) and CD4^+^ (fig. S5E) TILs isolated from MC38 tumor-bearing mice treated with anti–PD-L1 and apyrase compared to mice treated with anti–PD-L1, suggesting that intestinal conditioning by apyrase favored the infiltration of enterotropic T cells into the TME. An analogous increase in CCR9^+^ CD8 TILs upon apyrase treatment was observed in B16-OVA and LLC tumor-bearing mice as well (fig. S10, B and C). Consistent with the function of sIgA in promoting tumor infiltration by CCR9^+^ cells, this effect was lost in *IgA^−/−^* mice (Fig. 5D) as well as in vancomycin-treated mice (fig. S10D), whereas the proportion of CCR9^+^ CD8 TILs was similar between apyrase-treated and *L. johnsonii*–treated mice (fig. S10E).

A multitude of microbial and dietary antigens shapes the TCR repertoire of intestinal T cells. Recently, structural homologs of tumor-associated antigens able to elicit major histocompatibility complex (MHC) class I–restricted cytotoxic response have been identified in the microbiota of complete responders to ICB (*62*). Therefore, the presence of particular commensal bacteria in the microbiota might improve antitumor immunity by providing tumor-mimicking peptides for antigen presentation. To address whether the combination of apyrase with anti–PD-L1 could result in the expansion of particular T cell clones in the intestine and/or tumor bed, we analyzed the TCRVβ repertoire of CD8 T cells in the PPs and TME by flow cytometry. The comparison of mean frequencies of the different TCRVβ regions in the PPs of mice treated with anti–PD-L1 and *E. coli^pBAD28^* or *E. coli^pApyr^* (mean frequencies from four different groups of mice/treatment constituted by five mice per group) showed comparable values. In contrast, the frequencies of the different TCRVβ regions in the TME of differently treated mice were characterized by marked variability and a significant increase in CD8 T cells expressing the TCRVβ6 region in mice treated with *E. coli^pApyr^* versus *E. coli^pBAD28^* (fig. S10F). These data suggest tumor-specific T cell clones as well as variable bystander cells could be enriched in the TME following intestinal conditioning by apyrase.

Then, we tested whether these phenomena corresponded to enhanced migration of T cells from the intestine to the TME. We took advantage of photoconvertible KikGR mice that express a green-to-red photoconvertible protein under the ubiquitously expressed CAG promoter (*63*). We photoconverted the three terminal PPs in the ileum on day 11 after tumor engraftment and analyzed systemic cells 24 hours later (Fig. 5E). After 2.5 min of ultraviolet (UV) light exposure, most CD45^+^ cells in the PPs were photoconverted (fig. S11A). Analysis of T cells in the TME 24 hours after PPs photoconversion revealed that intestinal-derived CD45 TILs were found in the TME (Fig. 5F); anti–PD-L1 administration did not affect baseline migration from the intestine to the TME as compared to untreated tumor-bearing mice. However, the association of apyrase to anti–PD-L1 resulted in a significantly increased tumor infiltration by PP-derived CD8^+^ T cells as well as CD4^+^CD25^−^ T_conv_ cells, whereas we did not find significant variations in the abundance of gut-derived T_reg_ cells in the TME of differently treated mice (Fig. 5G and fig. S11B). Notably, the proportion of photoconverted CD8^+^ T cells in the TME of mice treated with apyrase negatively correlated with tumor size (Fig. 5H), suggesting that these cells could be proficient in controlling tumor growth during ICB. We did not find any substantial enrichment of CD8^+^ T or CD4^+^ T_conv_ or T_reg_ cells in tumor-draining and nondraining inguinal or mLNs or spleen of apyrase-treated mice (fig. S11, C to E), ruling out modified trafficking of these cells in lymphoid organs by apyrase. In summary, these data support the idea that intestinal conditioning by sIgA during ICB could improve the effector potential of gut T cells, which ultimately infiltrate the TME and exert antitumor cytotoxic activity. To address the function of enhanced *L. johnsonii* colonization in promoting the migration of intestinal CD8 T cells to the TME in mice treated with anti–PD-L1 combined with apyrase, we photoconverted ileal PPs in mice treated with anti–PD-L1, anti–PD-L1/apyrase, and anti–PD-L1 combined with oral gavage with *L. johnsonii*. The administration of *L. johnsonii* resulted in a significant increase in the abundance of photoconverted CD8^+^ TILs as compared to mice treated with standalone anti–PD-L1, displaying values similar to mice treated with anti–PD-L1/apyrase (Fig. 5I).

### CCL25 in the TME recruits gut-derived CD8^+^CCR9^+^ T cells and positively correlates with ICB responsiveness in patients with melanoma

**Fig. 6.**
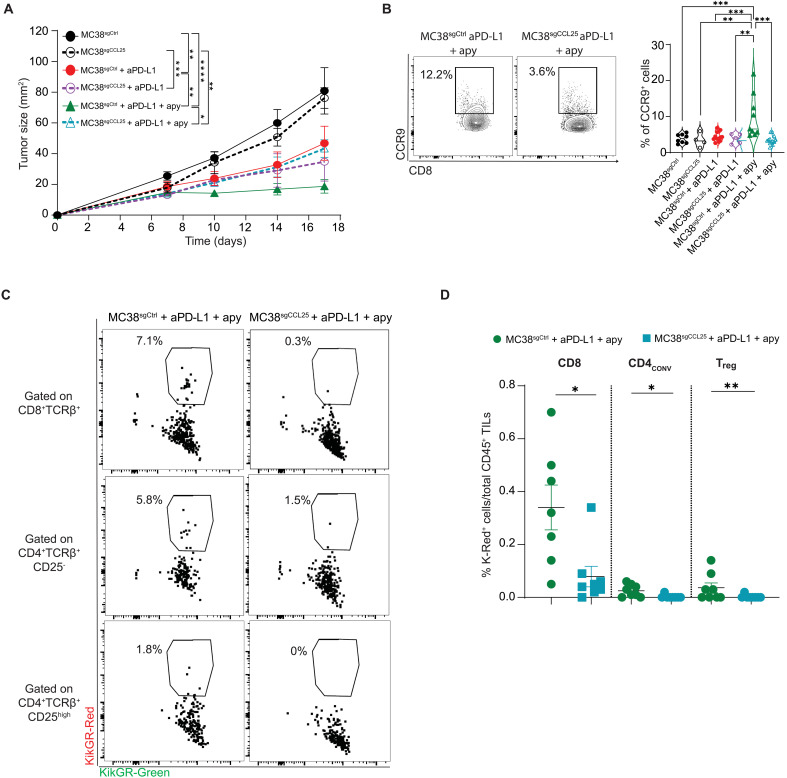
CCL25 in the TME recruits gut-derived CD8^+^CCR9^+^ T cells. (**A**) Tumor growth in mice subcutaneously engrafted with 1 × 10^6^ MC38^sgCtrl^ or MC38^sgCCL25^ cells and either untreated or treated with anti–PD-L1 alone or plus 40 μg of pure apyrase (as in Fig. 1A). Data are from one experiment representative of three (*n* = 6 to 8 mice per group). Error bars represent SEM. Two-way ANOVA for tumor growth was performed. (**B**) Representative dot plot and statistical analysis of CCR9-expressing CD8^+^ T cells in MC38^sgCtrl^ or MC38^sgCCL25^ tumors from mice treated as indicated. Data points represent single mice. Data were from two pooled experiments. Error bars represent SEM. One-way ANOVA with Tukey’s multiple comparison test was used. (**C** and **D**) Representative dot plots (C) and percentages (D) of K-Red CD8^+^, CD4^+^CD25^neg^ T_conv_ and T_reg_ cells within total CD45^+^ cells in tumors 24 hours postphotoconversion of mice engrafted with MC38^sgCtrl^ or MC38^sgCCL25^ cells and treated with anti–PD-L1 plus apyrase (as detailed in Fig. 5E). Data points represent single mice. Data were from two pooled experiments. Error bars represent SEM. The two-tailed Mann-Whitney *U* test multiple comparison test was used. *P < 0.05; **P < 0.01; ***P < 0.001.

Chemokines and their receptors control lymphocyte trafficking and play an instrumental role in shaping the immune tumor infiltrate. CCR9 is a gut-homing chemokine receptor expressed by T cells and other innate and adaptive immune cell subsets that preferentially reside in the small intestinal mucosa, where its sole ligand CCL25/TECK is expressed. In this respect, CCL25 expression in human solid tumors could further enhance the recruitment of CCR9-expressing cells. We then asked whether CCL25 expression by tumor cells could condition the extent of CCR9^+^ cell infiltration. To address this point, we used CRISPR-Cas9–mediated *Ccl25* down-regulation in MC38 cells (fig. S12A). The reduction of *Ccl25* expression resulted in the loss of the apyrase-mediated improvement of tumoricidal activity during ICB (Fig. 6A). Coherently, CD8^+^CCR9^+^ T cells were reduced in the TME (Fig. 6B). To test whether this impaired infiltration of CCR9^+^ CD8 TILs was effectively due to reduced migration from the gut, we implanted MC38 cells transduced with sg^Ctrl^ or sg^CCL25^ in KikGR mice and treated them with anti–PD-L1 and apyrase (as detailed in Fig. 5E). The accumulation of photoconverted CD8 TILs was significantly reduced in mice engrafted with MC38^sgCCL25^ cells as compared to mice bearing tumors transduced with sg^Ctrl^; similarly, the baseline migration of CD4^+^ T_conv_ and T_reg_ cells from the gut to the TME was reduced in MC38^sgCCL25^ as compared to control tumors (Fig. 6, C and D). In addition, CD8^+^ TILs expressing CCR9 produced IFN-γ more efficiently compared to their negative counterpart, suggesting an increased cytotoxic potential of this CD8 T cell subset (fig. S12B).

**Fig. 7.**
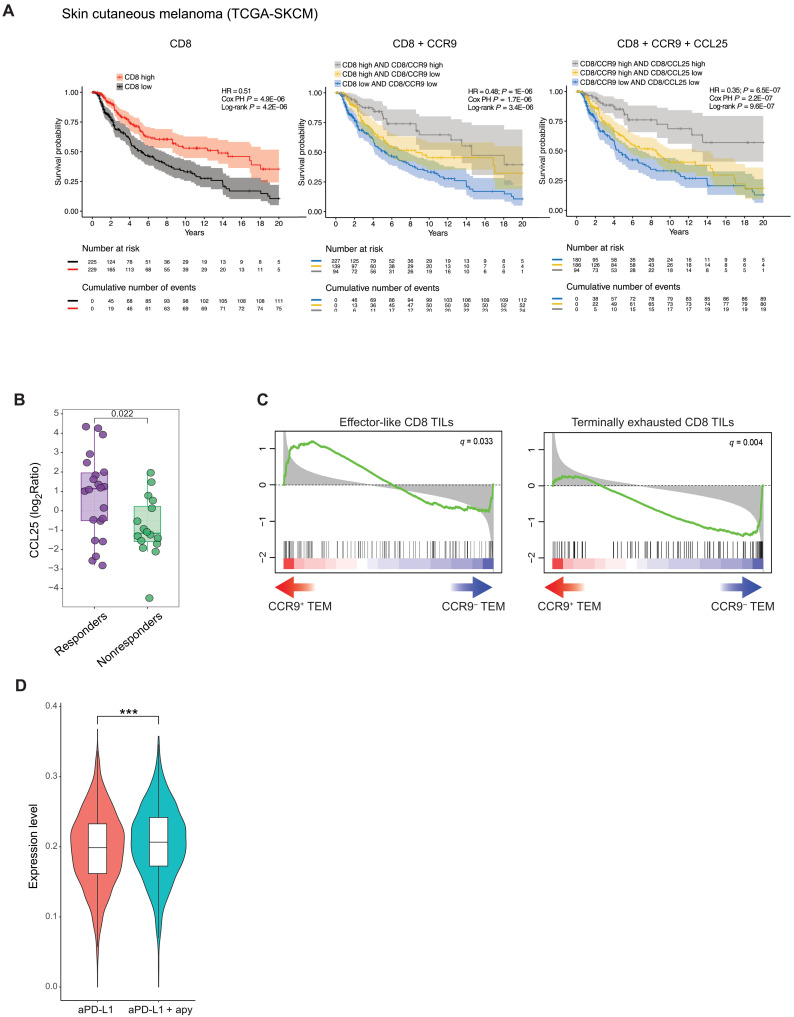
CCL25 in the TME positively correlates with ICB responsiveness in patients with melanoma. (**A**) Kaplan-Meier curves show the survival probability of patients with melanoma over 20 years, divided on the basis of the expression of CD8A and CD8B (left); CD8A, CD8B, and CCR9 (middle); and CD8A, CD8B, CCR9, and CCL25 (right). Graphs show the Cox PH ratio (HR), associated *P* value, and log-rank test results between groups. When more than two groups of patients were compared (middle and right), the Cox PH *P* value was calculated between both the “high_high” group versus all the other groups as a whole (first line) and any of the three groups (second line). The “Number at risk” table represents live individuals for each group at each time point. The “Cumulative number of events” table tracks deaths occurred up to the corresponding time point. (**B**) *CCL25* gene expression ratio between after and before anti–PD-L1 treatment in responder (purple) (*n* = 22) or nonresponder (green) patients with melanoma (*n* = 16). Data were taken from (*84*) and (*85*) and represent single patients. Error bar represents SEM. Two-tailed Mann-Whitney *U* test. *P < 0.022. (**C**) GSEA enrichment plots of gene sets from (*52*) significantly enriched or depleted in CD8^+^CCR9^+^ compared to CD8^+^CCR9^−^ T effector cells from PBMCs of healthy donors. Four healthy donors per group were used. (**D**) Expression of the CCR9 signature obtained by comparing bulk RNA-seq human CCR9^+^ and CCR9^−^ TEM cells on CD8 cells from scRNA-seq. Briefly, it was compiled by selecting differentially expressed genes with a *q* value lower than 0.01 and up-regulated in CCR9^+^ cells and converted to their murine orthologs. The expression of the signature in the scRNA-seq experiment was calculated for each cell as the average expression of the genes comprising the signature; significant difference was evaluated using the Wilcoxon rank sum test. ***P < 0.001.

In an effort to translate the T cell tumoricidal proficiency of enterotropic T cells into control of solid tumor growth in humans, we analyzed *CCL25* gene expression in human cancers. In most examined tumors, the gene encoding for CCL25 was overexpressed in neoplastic (in red) as compared to the adjacent normal tissue (in blue) (fig. S13A). To address the function of the CCL25/CCR9 axis in enhancing the antitumor CD8 T cell cytotoxic activity, we generated disease-specific survival (DSS) curves by comparing the effect of the combination of CCR9, CCL25, CD8A, and CD8B genes as a proxy for the infiltration of CD8^+^CCR9^+^ cells and production of CCL25 by the tumor. Notably, the combined expression of CCR9, CCL25, CD8A, and CD8B genes in tumors positively correlated with a higher DSS probability in patients with melanoma, with a better hazard ratio (HR) and *P* value compared to the expression of CD8A and CD8B genes only (Fig. 7A). Moreover, the analysis of public scRNA-seq data from CD8 T cells isolated from patients with melanoma (GSE123139) (*64*) revealed a gradient of increasing CCR9 expression in CD8 T cells from ICB-naïve patients to previously treated and on-treatment patients with melanoma (fig. S13B). Last, the analysis of variations of *CCL25* expression in tumor samples from patients with melanoma treated with anti–PD-1 (table S4) revealed that only responders displayed a significantly increased ratio between *CCL25* levels before and after ICB (Fig. 7B), suggesting that CCL25 bears the potential of being a predictive biomarker of antitumor response by ICB.

In adult human healthy donors, CCR9 is expressed by around 1% of circulating CD8^+^ T cells (fig. S13C). When we checked the distribution of T cells subsets among CCR9^+^ and CCR9^−^ CD8 T cells (according to CCR7 and CD45RA expression), we found that CCR9^+^ cells were enriched for T central memory (T_cm_) cells (CCR7^+^CD45RA^−^) while depleted for terminally differentiated effector T (T_emra_) cells (CCR7^−^CD45RA^+^) (fig. S13D). This functional cell subset distribution suggests that CCR9^+^ CD8 T cells may bear greater expansion and effector potential and thus could be exploited for immunotherapeutic strategies. Last, we isolated CCR9^+^ and CCR9^−^ CD8 effector T cells from healthy donors and ran bulk RNA-seq. Genome-wide comparative transcriptional analysis of circulating CD8^+^CCR9^+^ versus CD8^+^CCR9^−^ effector T cells from human healthy donors revealed an enrichment of genes characterizing effector-like CD8 TILs with antitumor activity and pauperization of genes belonging to a terminally exhausted like signature in CCR9^+^ cells (*52*) (Fig. 7C). As *Ccr9* was expressed at low levels in single cells, which likely led to a high dropout rate and lack of detection of the transcript by 10X Genomics scRNA-seq, we projected the genes differentially expressed in human CD8^+^CCR9^+^ versus CD8^+^CCR9^−^ effector cells on the CD8 cluster of our mouse scRNA-seq. We observed a significant enrichment of genes characterizing CCR9^+^ human effector cells in CD8 TILs from mice treated with anti–PD-L1 and apyrase compared to standalone anti–PD-L1 (Fig. 7D). Together, these results point to the intestinal environment as a crucial hub in the immune system for conditioning the antitumor immune response and sIgA as an inducible safeguard of small intestinal fitness that may hold true also in human clinical settings.

## DISCUSSION

The intestinal microbiota community was recently shown to have a decisive impact on the outcome of ICB in patients with solid tumors (*16*, *17*). Beneficial microbiota modifications in patients experiencing response to ICB after FMT resulted in the enrichment of CD8 T cells infiltrating the tumor and contacting melanoma cells as well as an increase in circulating ICOS^+^CD8^+^ T cells (*18*). Therefore, modulating the gut microbiota represents a promising approach to enhance the success rate of immunotherapies. Apart from FMT, selected live biotherapeutics, bacterial products, and engineered microbes are already used in clinical trials with oncologic patients in combination with ICB (*65*).

The gut mucosal immune system and the microbiota have a reciprocal relationship; in this respect, sIgA coating plays an instrumental function in host-microbiota mutualism by supporting beneficial bacteria while excluding from epithelial contact harmful pathogens (*27*, *66*). The interaction between B and T_FH_ cells in the PPs results in the production of affinity-matured sIgA by plasma cells in the ileal mucosa (*67*). Extracellular ATP has emerged as a bacterial metabolite present in the intestinal lumen at concentrations sufficient to permeate PPs and limit T_FH_ cell activity via P2X7R (*23*). Administration of the ATP-degrading apyrase proved to be successful in amplifying the sIgA repertoire and correcting antibiotic-induced dysbiosis (*26*). In the present study, we demonstrated that apyrase can be used to improve intestinal fitness and promote the adaptive antitumor immune response during ICB. This adjuvant therapy led to an increased production of tumoricidal cytokines and infiltration of functionally active CD8 T cells. Among the transcripts selectively up-regulated by apyrase administration, we detected *Il7r*, which characterizes a stemlike and tumor-responsive CD8 T cell population capable of sustaining the therapeutic response induced by ICB (*55*, *68*–*70*). The gene signature obtained by scRNA-seq of CD8 TILs from mice treated with anti–PD-L1 combined with apyrase correlated with the response to immunotherapy in patients with melanoma (*55*), suggesting that apyrase-elicited conditioning of the gut ecosystem has the potential to increase the response rate to ICB in patients with cancer. The combination of apyrase to anti–PD-L1 resulted in improved responsiveness of CD8^+^ T cells residing in the small intestine, prompting us to speculate that this functional invigoration could beneficially reflect on the antitumor response. This hypothesis was corroborated by experiments in photoconvertible KikGR mice (*63*) bearing MC38 tumors, in which we could show the enhanced migration of photoconverted CD8 T, but not T_reg_ cells, from the gut to the TME by combining apyrase to anti–PD-L1 administration. Tumor-specific T cells in the gut can arise via molecular mimicry of tumor associated antigens. In addition, beyond direct antigen mimicry, bystander activation of gut-derived T cells may also contribute to the control of tumor growth. Whereas the various TCRVβ regions were uniformly expressed in CD8 T cells isolated from the PPs of differently treated mice, a significant increase in TCRVβ6-expressing cells was observed in CD8 TILs from mice treated with anti–PD-L1 and apyrase as compared to mice treated with standalone anti–PD-L1, suggesting that particular clonotype/s were selectively enriched within the tumor bed and could eventually confer enhanced control of tumor growth.

The deficiency of IgA in both mice and humans results in systemic immune dysregulation, enhanced bacterial translocation, and elevated levels of systemic IgG bound to bacteria (*29*). The importance of sIgA in our preclinical proof of concept for apyrase function in ICB was demonstrated by (i) the percentage of ileal sIgA-coated bacteria negatively correlated with the tumor size of mice treated with anti–PD-L1 independently of the addition of apyrase; (ii) the adjuvant activity of apyrase during ICB was lost in mice lacking IgA. We addressed which bacterial components of the microbiota were preferentially targeted by the amplified sIgA repertoire induced by apyrase. Both IgA sequencing (IgA-seq) and full-length 16*S* rRNA gene sequencing revealed *Lactobacillus*, in particular *L. johnsonii* and *L. reuteri*, which are known to exert an antitumor effect (*8*, *58*, *71*, *72*), as possible contributors to the therapeutic effect. Abrogation of the therapeutic effect of apyrase by vancomycin treatment suggested that *L. johnsonii* played a major role. This finding is interesting with respect to the human response to ICB because *L. gasseri*, belonging to the same phylogroup of *L. johnsonii*, had consistently baseline higher and increasing overtime abundances in ICB-treated patients with advanced melanoma showing significantly extended progression-free survival (*73*). We have shown the enhanced migration to the tumor bed and tumoricidal function of enterotropic T cells as well as improved control of tumor growth by daily gavaging of *L. johnsonii* in IgA-competent but not IgA-depleted mice. This result directs to the relevance of sIgA and *L. johnsonii* richness in improving the efficacy of ICB through a gut-tumor axis involving the migration of invigorated cytotoxic T cells.

Dysbiosis is often linked to ICB, which frequently causes gastrointestinal irAEs (*37*). In addition, cancer itself can result in severe ileopathy (*74*). Patients responding to ICB after FMT showed significantly reduced serum levels of IL-1R–like 1 (IL-1RL1 or ST2) protein, a marker of heightened intestinal permeability, thereby suggesting that an enhanced intestinal epithelial integrity can contribute to ICB outcome (*18*). We have shown that anti–PD-L1 treatment in MC38 tumor-bearing mice increases PV-1 expression in the ileum, consistent with a negative impact of ICB on vascular gut barrier. Supplementation of apyrase restored PV-1 expression to baseline levels, further supporting the role of apyrase in improving the intestinal barrier integrity during dysbiosis (*26*). Severe intestinal irAEs, which characterizes ICB failure and threatens life of patients, can be reproduced in mice by combining oral DSS to anti–CTLA-4 and anti–PD-L1 administration (*49*). Provision of apyrase in this experimental setting resulted in the maintenance of intestinal integrity, further extending the usefulness of this adjuvant approach in the prevention of this severe condition.

Lymphocyte migration from the intestine into the tumor is an interesting possible mechanism by which gut microbiota, probiotics, or metabolites might augment the antitumor immune response. When we examined the phenotype of TILs following oral administration of apyrase, we observed that CCR9 expression was significantly augmented in CD8 and CD4 T cells infiltrating the TME. According to a possible beneficial influence of these cells in the control of cancer progression, CCR9^+^ T cells in the melanoma microenvironment have been demonstrated to inhibit metastasis (*61*). Moreover, intratumoral delivery of CCL25 enhanced the response to immunotherapy in triple-negative breast cancer by recruiting CCR9^+^ T cells (*75*). We addressed whether intratumoral CCL25 could influence the therapeutic effect of apyrase by knocking down the *Ccl25* gene in MC38 cells. This intervention was sufficient to inhibit the recruitment of CCR9^+^ T cells in the TME and abrogate tumor growth control by apyrase. We analyzed variations of *CCL25* expression in tumor samples from patients with melanoma treated with anti–PD-1 and showed that sustained CCL25 expression during ICB characterized patients responding versus nonresponding to the therapy. We observed that, in adult human healthy donors, CCR9 is expressed by a small subset of circulating T cells, presumably originating from the intestine, which represents around 1% of circulating CD8 T cells. This cell subset expresses increased levels of CD25, CD69, CD71, and HLA-DR as compared to homologous CCR9^−^ cells, thereby indicating CCR9 expression is associated to an activated phenotype (*76*). The gene signature of these cells showed the enrichment of genes involved in robust antitumor functionality and durable response. Together, these data suggest that conditioning of the gut ecosystem by apyrase-mediated sIgA amplification could promote the expansion of proficient intestinal CD8 T cells in oncologic patients for obtaining durable antitumor responses during ICB.

## MATERIALS AND METHODS

### Study design

The aim of this study was to investigate how sIgA amplification could beneficially condition the small intestinal ecosystem and enhance the antitumor response during cancer immunotherapy. Experimental approaches included scRNA-seq to investigate the gene signature of CD8 TILs in differently treated mice and to compare it with publicly available dataset from human samples. We used immunofluorescence and TEM to investigate structural features in the ileal epithelium, which could be induced by ICB and modified by our treatment. PacBio 16*S* sequencing was used to determine ileal bacteria composition at the strain level. In addition, we used photoconvertible mice to monitor in vivo the migration of enterotropic T cells from the intestine to peripheral tissues. Immune system and epithelial cells, and bacteria were further characterized by various downstream analyses, including multiparameter flow cytometry, bulk RNA-seq, and enzyme-linked immunosorbent assay (ELISA). Sample sizes used reflect animal or material availability. Numbers of biological repeats are provided in the figure legends, and no randomization or blinding was used.

### Mice

C57BL/6, BALB/c, and *Igha^−/−^* (Ighatm1Grh) mice were bred in specific pathogen–free (SPF) facility at the Institute for Research in Biomedicine (Bellinzona, Switzerland). B6.Cg-Gt(ROSA)26Sor<tm1.1(CAG-kikGR)Kgwa> (B6-ROSA/kikGR KI) was provided by M. Tomura (RIKEN BioResource Research Center, Ibaraki, Japan) and bred in homozygosis in our SPF facility. All animal experiments were performed in accordance with the Swiss Federal Veterinary Office guidelines and approved by the Ethical Committee of the Cantonal Veterinary, with authorization numbers 31985, 33464, 33489, and 35407. For all the experiments, mice (7 to 9 weeks old, age and sex matched) were used. Mice were housed, up to five per cage, in ventilated cages under standardized conditions (20° ± 2°C, 55 ± 8% relative humidity, and 12-hour/12-hour light/dark cycle). Food and water were available ad libitum, and mice were examined daily.

### Tumor cell lines

Melanoma B16F10-OVA (B16-OVA) were obtained from M. Bellone (HSR Scientific Institute, Milan, Italy), colon adenocarcinoma MC38 cells were obtained from Kerafast (National Cancer Institute/NIH), B16 melanoma cells expressing the ATP reporter plasma membrane luciferase (B16-pmeLUC) were obtained from F. Di Virgilio (University of Ferrara, Italy), and colon carcinoma (CT26) and LLC cells were purchased from the American Type Culture Collection. MC38, B16-OVA, and CT26 were cultured in RPMI 1640 supplemented with 10% heat-inactivated fetal bovine serum (FBS), penicillin/streptomycin (100 U/ml), kanamycin (100 U/ml), and 2 mM GlutaMAX (RPMIc+), whereas LLC cells were cultured in Dulbecco’s modified Eagle’s medium (DMEM) supplemented with 10% heat-inactivated FBS, penicillin/streptomycin (100 U/ml), and 2 mM GlutaMAX. Frozen aliquots were thawed for each in vivo experiment and passaged in vitro for the minimum time required. Tumor cells at 70 to 80% confluence were harvested by diluting them 1:5 in 0.25% trypsin. Cells were tested for the absence of *Mycoplasma* and maintained in 5% CO_2_ at 37°C.

### Culture conditions of bacterial strains

*E. coli^pBAD28^* or *E. coli^pApyr^* were grown in LB broth supplemented with l-arabinose (0.03%), ampicillin (100 μg/ml), and chloramphenicol (30 μg/ml) at 37°C and 220 rpm. The pBAD promoter is constantly active in the mouse gut lumen.

### Purification of recombinant apyrase

The apyrase gene from *S. flexneri* was cloned into the pET21a vector (Novagen, 69740) with a C-terminal histidine tag. The construct was codon optimized for expression in *E. coli* by GeneScript. The plasmid was transformed into Rosetta (DE3)pLysS cells (Novagen, 70956) and plated on LB agar (Sigma-Aldrich, L2897) supplemented with ampicillin (100 μg/ml) and chloramphenicol (34 μg/ml). After overnight incubation, a single colony was picked and inoculated into 50 ml of LB medium (Sigma-Aldrich, L3032) supplemented with ampicillin (100 μg/ml) and chloramphenicol (34 μg/ml). The culture was grown overnight to prepare the starter culture. For purification of apyrase, induction of a 4-liter culture was performed by adding 0.5 mM isopropyl-β-d-thiogalactopyranoside (IPTG; Sigma-Aldrich, I6758). The culture was harvested by centrifugation at 6000 rpm for 10 min at 4°C (Fiberlite F9-6 x 1000, Thermo Fisher Scientific), and the resulting cell pellet washed in 10 mM Tris (pH 8.0), 150 mM NaCl, and 1 mM EDTA. The cell pellet was then resuspended in 160 ml of 50 mM tris-HCl (pH 7.5) and snap frozen in liquid nitrogen. The frozen cell pellet was thawed by immersion in room temperature (RT) water and supplemented with 300 mM NaCl and 40 mM imidazole (Sigma-Aldrich, I202). The cell suspension was sonicated and clarified by centrifugation at 15,000 rpm for 45 min at 4°C (Fiberlite F20-12 x 50 LEX, Thermo Fisher Scientific). The clarified lysate was passed through a column (Pierce Disposable Columns, 10 ml, Thermo Fisher Scientific) containing 4 ml of Ni-NTA agarose beads (Qiagen) by gravity flow. The column was then washed twice with 30 ml of lysis buffer [50 mM tris-HCl (pH 7.5), 300 mM NaCl, and 40 mM imidazole]. The bound protein was eluted in 1-ml fractions with the addition of 20 ml of elution buffer [50 mM tris-HCl (pH 7.5), 300 mM NaCl, and 300 mM imidazole]. Aliquots of the collected fractions were loaded onto an SDS–polyacrylamide gel electrophoresis gel and stained with Imperial Protein Staining (Thermo Fisher Scientific) following the manufacturer’s instructions to determine the amount and purity of the enzyme. Fractions containing apyrase were pooled and concentrated to 5 ml using a 10-kDa Molecular Weight Cut-Off (MWCO) spin concentrator (Amicon Ultra-15, Millipore) and loaded onto a HiLoad S-75 size exclusion column (Cytiva) preequilibrated with phosphate-buffered saline (PBS; Sigma-Aldrich, D8537). The peak fractions were pooled and concentrated to ~2 mg/ml using a spin concentrator. The purified protein was filter sterilized using a 0.22-μm Millex-GP filter (Millipore) and stored in aliquots under appropriate conditions.

### Tumor model and in vivo treatments

B16OVA, MC38, CT26, and LLC were harvested at exponential growth. On day 0, B16OVA, MC38, and CT26 were resuspended in PBS at a concentration of 1 × 10^7^ cells/ml, whereas for LLC, a concentration of 0.6 × 10^7^ and a volume of 0.1 ml (1 × 10^6^ or 6 × 10^5^ tumor cells) was injected subcutaneously into the back of C57BL/6, BALB/c, *IgA^−/−^*, or KikGR mice. On days 8, 11, 14, and 17 after tumor inoculation, mice were treated intraperitoneally with 100 μg of anti–PD-L1 (10F.9G2, BioXCell), anti–CTLA-4 (9D9, BioXCell), or anti-CD40 (FGK4.5, BioXCell) antibody. For apyrase administration, mice were gavaged everyday with PBS, 40 μg of pure apyrase, or 1 × 10^10^ of *E. coli^pApyr^*/*E. coli^pBAD28^* from day 5 until the end of the experiment. For antibiotic treatment, vancomycin (GoldBio) was given in water ad libitum at a concentration of 200 mg/liter starting from 2 weeks before tumor implantation. For experiments with *L. johnsonii*, mice were daily orally gavaged with 10^9^ bacteria starting from day 5 from tumor injection. To quantify extracellular ATP in the TME, mice engrafted with B16-pmeLUC were anesthetized with 2.5% isoflurane and intraperitoneally injected with d-luciferin (150 mg/kg; Promega) and after a 15-min interval allowing for biodistribution, luminescence was captured from dorsal view. Photon emission was quantified using the Living Image software (PerkinElmer) and averaged as photons/seconds/cm^2^/steradian (abbreviated as p/s/cm^2^/s). To block the egress of IgA^+^ B cells, FTY720 (1 mg/kg; Cayman Chemicals) was given intraperitoneally 1 day before the initiation of anti–PD-L1 therapy regimens and continuously every second day until day 18 posttumor inoculation. For the mouse model of irAEs, mice were given 2% DSS (MP Biomedicals) in drinking water from day 0 for a total of 3 days, followed by 4 days of water. Two days before DSS treatment, a group of mice also received daily gavage of 40 μg of pure apyrase until the end of the experiment. On days 0, 3, and 6, mice received intraperitoneally either 100 μg of anti–CTLA-4 or 100 μg of anti–PD-L1 or IgG. Intestinal permeability was assessed using fluorescein isothiocyanate (FITC)–dextran assay. Mice were starved for 4 hours before treatment and then orally gavaged with 6 mg of FITC-dextran (molecular weight, 4000 Da) (46944, Sigma-Aldrich) in PBS. Blood was collected 4 hours posttreatment, the serum samples were diluted 1:2 with PBS with 25 mM Hepes (Gibco), and the fluorescence intensity was measured in the serum at 485/530 nm using a microplate reader (Biotek Synergy 2). Tumor growth was scored every 2/3 days with a caliper by measuring the greatest tumor diameter and its perpendicular to determine an average, and then the area was calculated as follows: (average/2)2π. For survival experiments, mice were euthanized when they reached humane endpoint, defined by tumor volume [estimated with the formula (length × width^2^)/2] more than 1 cm^3^ or when mice showed clinical signs of suffering. Upon euthanasia, tumors were directly excised from the skin using forceps. The small intestine, PPs, mLNs, inguinal lymph nodes (iLNs), and spleen were harvested and processed as described below.

### Cell isolation from tissues

Tumors were cut in small pieces and resuspended in RPMI 1640 with type I collagenase (1.5 mg/ml; Sigma-Aldrich), DNase I (100 μg/ml; Roche), and 5% FBS and digested for 45 min at 37°C under gentle agitation. The digestion product was then passed through a 70-μm cell strainer to obtain a single-cell suspension. Lymphocytes are then enriched by Percoll density gradient following the manufacturer’s protocol, and a single-cell suspension was counted before FACS analysis, making exception for tumors from KikGR mice, which were analyzed by FACS without performing Percoll enrichment. Spleens, PPs, mLNs, and iLNs are minced through 70-μm cell strainers. For spleens, cells are resuspended in ACK buffer (8.26 g of ammonium chloride, 1.0 g of potassium bicarbonate, and 0.037 g of EDTA in 1 liter of ultrapure water) to lyse red blood cells for 6 min and then washed twice in PBS. Cells are resuspended in RPMI with 10% FBS, and viable cells are counted by trypan blue exclusion. For intestinal lamina propria cell purification, PPs were removed from the ileum, and then it was washed thrice with ice-cold PBS and digested at 37°C for 30 min with RPMI added with 5 mM EDTA for two times to remove epithelial cells. The filtrated fragments were then digested in RPMI with 5% FBS, collagenase D (1 mg/ml) from *Clostridium histolyticum* 100 mg (Roche), and DNase I grade II (40 μg/ml; Roche) for 40 min. The filtered suspension was washed with RPMI with 10% FBS, and a single-cell suspension was used for FACS analysis using the BD Symphony A5 platform.

### Antibodies and flow cytometry

The following anti-mouse monoclonal antibodies (mAbs) were purchased from eBioscience: phycoerythrin (PE)–conjugated anti–4-1BB (clone: 17B5, catalog no. 251371 82), PercP/Cy5.5-conjugated anti-CCR9 (clone: CW-1.2, catalog no. 46-1991-82), and PE-conjugated anti–Ki-67 (clone: SolA15, catalog no. 12-5698-82). The following mAbs were purchased from BioLegend: AF700-conjugated anti-CD45 (clone: 30-F11, catalog no. 103127), PE-Cyanine7–conjugated anti-CD3 (clone: 145-2C11, catalog no. 100320), Pacific Blue–conjugated anti-CD8α (clone: C398.4A, catalog no. 313516), Allophycocyanin (APC)-Cyanine7–conjugated anti-CD4 (clone: GK1.5, catalog no. 100414), PE/Cy7-conjugated anti–IFN-γ (clone: XMG1.2, catalog no. 505808), APC-conjugated anti–IL-7Rα (clone: A7R34, catalog no. 135007), BV786-conjugated anti–PD-1 (clone: 29F.1A12, catalog no. 135225), FITC-conjugated anti-EPCAM (clone: G8.8, catalog no. 118210), BV605-conjugated anti–T-bet (clone: 4B10, catalog no. 644817), PercP/Cy5.5-conjugated anti–TNF-α (clone: MP6-XT22, catalog no. 506322), and APC-conjugated anti–Granzyme B (clone: BG11, catalog no. 515405). The following mAbs were purchased from BD Biosciences: anti-BUV737–conjugated anti-TCRβ (clone: H57-597, catalog no. 612821), BUV805-conjugated anti-CD8α (clone: 53-6.7, catalog no. 612898), biotin-conjugated anti-CXCR5 (clone: 2G8, catalog no. 551960), and BUV395-conjugated anti-CD45 (clone: 30-F11, catalog no. 564279). IL-21 was detected with a recombinant mouse IL-21R subunit/human IgG1 Fc chimera (R&D Systems, catalog no. 596-MR) with goat anti-human Fcγ conjugated to AF488 (Jackson ImmunoResearch, catalog no. 109-095-098). For surface staining, cells were stained with antibodies against surface antigens in blocking buffer after blocking unwanted antibody binding to Fc receptors using Fcγ receptors binding inhibitor. Viability dye (BD Bioscience) was used to exclude dead cells. For tetramer staining, 1 × 10^6^ cells were labeled for 2 hours at 37°C with H-2Kb OVA Tetramer SIINFEKL-PE (MBL, iTAg MHC tetramer, catalog no. T0300). Intracellular staining was performed using the eBioscience (for transcription factors) or BD (for cytokines) Cytofix/Cytoperm and Perm/Wash buffers. For cytokine staining, cells were incubated for 4 hours at 37°C in ionomycin (750 ng/ml) and PMA (phorbol 12-myristate 13-acetate; 20 ng/ml). For the last 3 hours, brefeldin (eBioscience, 1000X Solution) was added to the cultures. Samples were acquired on LSRFortessa (BD Bioscience) or BD FACSymphony flow cytometers (BD Bioscience). Data were analyzed using FlowJo software (TreeStar) or FACS Diva software (BD Biosciences).

### RNA extraction and scRNA-seq analysis of tumor-infiltrating immune cells

scRNA-seq was performed immediately after FACS of CD45^+^ TILs with the Chromium Next GEM Single Cell 3′ Reagent Kits v3.1 (10X Genomics, Pleasanton, CA) following the manufacturer’s protocol. The target number of captured cells ranged from 2000 to 10,000 cells. Sequencing libraries were prepared per the manufacturer’s protocol. Sequencing was performed on a NextSeq2000 (Illumina Inc., San Diego, CA) at a median depth of 115,216 reads per cell. The 10x Cell Ranger Count pipeline (version 3.1.0) was used to align reads to the reference transcriptome (mm10) and to calculate Unique Molecular Identifier (UMI) counts from the mapped reads. Expression data were imported in R (version 4.0.3) and analyzed using Seurat (version 4.0.0) (*77*) and popsicleR (version 0.2.1) (*78*) R packages. For each sample, low-quality cells were identified as outliers within the distribution of the number of genes, UMI counts, and percent of reads mapping on mitochondrial genes per cell and subsequently discarded. Doublets were identified and discarded using Scrublet (version 0.2.1) (*79*). Samples were integrated using the Seurat integration strategy (FindIntegrationAnchors and IntegrateData functions). Before dimensional reduction with principal components analysis (PCA), cell cycle scores were assigned to each cell and regressed out. We selected 10 principal components for cluster analysis; clusters representing T cells were identified using the expression level of the CD3 gene. These cells (*n* = 3751 in seven samples) were subsequently reintegrated and reclustered using the same procedure described above. We classified T cells clusters monitoring the expression of known markers. Differentially expressed genes between CD8 T cells infiltrating tumors treated with anti–PD-L1 and CD8 T cells infiltrating tumors treated with anti–PD-L1 plus apyrase were identified using the FindMarkers function in Seurat. Expression of gene signatures related to T cell biology was calculated as the average expression of the genes comprising the signature in each cluster, and significant differences were evaluated with the Mann-Whitney test and Benjamini and Hochberg correction.

### Quantification of extracellular ATP in the intestine

For quantification of extracellular ATP in the ileum, intestinal content was collected by lavage with 5 ml of intestinal wash buffer (PBS, 0.5 M EDTA, soybean trypsin inhibitor, and phenylmethylsulfonyl fluoride), spun, and filtered (0.22 μm) to remove any bacteria-sized contaminants and immediately frozen in dry ice. ATP concentration in the intestinal washes was multiplied for the dilution factor to obtain the actual endoluminal ATP concentration (*25*). The extracellular ATP concentration was evaluated by bioluminescence assay with recombinant firefly luciferase and its substrate d-luciferin according to the manufacturer’s protocol (Life Technologies Europe B.V.).

### Ileal IgA flow cytometry and sorting of IgA^+^ bacteria

For analysis of IgA-coated bacteria in flow cytometry, fresh ileal pellets were collected into sterile 1.5-ml Eppendorf tubes and homogenized in reduced PBS (0.1 g/ml). Large debris was separated by centrifugation (50*g*, 15 min, 4°C), and the clarified supernatant was passed through a 70-μm filter. For flow cytometry of IgA-coated bacteria, 20 μl of supernatants was resuspended in reduced PBS and centrifuged at 8000*g* for 10 min to remove unbound IgAs, whereas for IgA sorting, all the supernatant was collected. Bacterial pellets were resuspended in PBS with 10% rat serum and incubated for 10 min on ice. An additional 1 ml of PBS was added to the blocking suspension and centrifuged, and pelleted bacteria were washed with 1 ml of FACS buffer [PBS + 1% bovine serum albumin (BSA)]. Pellets were stained with APC-conjugated rabbit anti-mouse IgA antibodies. After 30-min incubation, bacteria were washed twice and resuspended in 2% paraformaldehyde (PFA) in PBS for acquisition on LSRFortessa or BD FACSymphony. SYTO BC was added to identify bacteria-sized particles containing nucleic acids. Both for analysis and sorting of the IgA^+^ fraction on FACSAria, forward scatter (FSC) and side scatter (SSC) parameters were used in logarithmic mode, and the threshold was set at 200. *IgA^−/−^* mice were used as negative controls.

### Enzyme-linked immunosorbent assay

Fresh ileal pellets were collected into sterile 1.5-ml Eppendorf tubes and homogenized in PBS (0.1 g/ml). The samples were then centrifuged two times for 10 min at maximum speed. ELISA plates (Corning half-area 96-well plate) were coated (16 hours at 4°C) with 25 μl of unlabeled goat anti-murine IgA (Southern Biotech) at a concentration of 5 μg/ml in 1X PBS, washed four times with PBS with 0.025% Tween 20, and saturated with 50 ml of PBS with 1% BSA (Sigma-Aldrich) for 1 hour at RT. Twenty-five microliters of serial dilutions of the different samples was incubated for 2 hours at RT. After four washes in PBS with 0.025% Tween 20, 25 μl of alkaline phosphatase–conjugated goat anti-mouse IgA (Southern Biotech) (1:500 in PBS with 1% BSA) was added and plates were incubated for 2 hours at RT. The assay was developed with 4-nitrophenyl phosphate disodium salt hexahydrate (Sigma-Aldrich) in carbonate buffer, and the absorbance was detected at 405 nm.

### 16*S* rRNA gene sequencing and data analysis for IgA-seq

Library preparation and sequencing: The starting input for 16*S* rRNA sequencing starts at 5 ng of isolated genomic DNA. Libraries are constructed by amplification via PCR with primers covering the V3 to V4 region (341F and 805R). Sequencing was performed on an Illumina MiSeq platform [2x250 base pairs (bp)]. The reads were analyzed by QIIME2. The raw sequences were 1,731,653 (average: 123,689; median: 121,036). Afterward, the filtering and denoising and merging were performed by DADA2, and the clean reads were 206,245 (average: 14,732; median: 14,472). The taxonomic assignment was performed by the BLAST feature classifier. Then, it assigns consensus taxonomy to each query sequence on the last database version of Greengenes (gg_12_10). To delve deeper into taxonomy assignment, manual blastn local alignment was executed between the query and reference reads from the 16*S* rRNA database. The retained ASVs were multialigned by applying MAFFT (PMID: 23329690), and the obtained multiple sequence alignment (MSA) was used to build a maximumlikelihood (ML) phylogenetic tree in Fasttree 2 (PMID: 20224823). Differential abundance analyses of bacterial taxa to establish the IgA index were carried out using the DESeq2 package within the R environment (version 4.3.1). The dataset included microbial profiles obtained from 16*S* rRNA gene sequencing, and the analysis focused on ASV-level taxonomic resolution. The aim was to investigate microbial shifts across four specific pairwise comparisons: *aPD-L1 IgA^+^* versus *aPD-L1 unsorted*, *aPD-L1 IgA^+^* versus *aPD-L1 IgA^−^*, *aPD-L1/apyrase IgA^+^* versus *aPD-L1/apyrase unsorted*, and *aPD-L1/apyrase IgA^+^* versus *aPD-L1/apyrase IgA^−^*. For each comparison, count matrices derived from raw, non-normalized read abundances were imported into DESeq2, and a DESeqDataSet object was constructed, assigning the appropriate experimental condition as the design variable. Data normalization was performed using DESeq2’s internal method on the basis of the estimation of size factors, which accounts for differences in sequencing depth across samples. Statistical inference for differential abundance was performed using the Wald test as implemented in DESeq2. To control for multiple hypothesis testing, *P* values were adjusted using the Hochberg correction method. Taxa with adjusted *P* values below 0.05 were considered significantly differentially abundant between groups. Log₂ fold changes were computed for each taxon to quantify the direction and magnitude of change between the conditions under comparison.

### 16*S* rRNA gene sequencing for full-length 16*S* PacBio rRNA sequencing

Two nanograms of total DNA extracted from ileum content of mice was used as the template for library preparation. Sequencing libraries were prepared by amplifying the full-length 16*S* rRNA gene, following the method described in (*80*). The SMRTBell library was sequenced on the PacBio Sequel System II platform. Output files were processed and assembled into CCS reads. The workflow for processing raw data obtained with the PacBio platform following the standard software tools provided by the manufacturer’s protocol (*81*). ASVs were taxonomically annotated by using the QIIME2 classify-sklearn plugin and the release 138 NR 199 of the SILVA database (PMID: 17947321). The reads were analyzed by QIIME2. The raw sequences were 583,370 (average: 36,461; median: 37,254). After the processing the reads by DADA2, comprising filtration, denoising, and merging, the resulting clean reads were 240,572 (average: 15,034; median: 14,915). ASVs resulting from the DADA2 process were taxonomically annotated by using the QIIME2 classify-sklearn plugin and the release 138 NR 199 of the SILVA database (PMID: 17947321). The retained ASVs were multialigned by applying MAFFT (PMID: 23329690), and the obtained multiple sequence alignment (MSA) was used to build an ML phylogenetic tree in Fasttree 2 (PMID: 20224823).

### qPCR analysis of *L. johnsonii*

The same DNA used for PacBio SMRTbell sequencing was used for the quantification of *L. johnsonii* by qPCR. Briefly, 5 μl of DNA was mixed with 7.5 μl of EvaGreen Supermix (Bio-Rad) and 0.75 μl of 10 mM forward and reverse primers. The qPCR cycling conditions were as follows: for EUB primers: 95°C for 3 min, followed by 40 cycles of 95°C for 10 s and 60°C for 30 s (*82*); for *L. johnsonii* primers: 95°C for 3 min, followed by 40 cycles of 95°C for 10 s and 60°C for 30 s. A melting curve analysis was performed at the end of each run to confirm amplification specificity. Data were analyzed using Bio-Rad CFX Maestro software. The primer sequences used were as follows: EUB F: 5′-TCCTACGGGAGGCAGCAGT-3′, EUB R: 5′-GGACTACCAGGGTATCTAATCCTGTT-3′; LJ1F: 5′-TGGTTAAATCAGAGCCTGCTT-3′, LJ1R: 5′-TTGCGGTAAAACATCCCCAG-3′. *The L. johnsonii*–specific primers were designed by aligning publicly available *L. johnsonii* genomes from the NCBI with closely related species (*L. gasseri*, *L. paragasseri*, *L. taiwanensis*, *and L. jensenii*) and selecting regions uniquely present in *L. johnsonii* genomes.

### Quantification of PV-1 expression by confocal microscopy

For the imaging of the ileum, intestines were dissected immediately following euthanasia and sections of ileum 10 to 20 mm in length were washed twice in ice-cold 1X PBS and immersion fixed in 2% PFA overnight at 4°C with gentle agitation followed by washes in 1X PBS. The tissues were then embedded in 3% low-gelling temperature agarose and were sectioned using a Leica VT1200S vibratome to produce 80-μm slices. Sections for immunofluorescence were blocked in blocking buffer (PBS, 1% FBS, 0.1% Triton X-100, and 0.01% NaN_3_) for 1 hour at RT and then stained with antibodies anti–PV-1 overnight in blocking buffer. Following three 20-min washes, sections were stained for the secondary antibody for 1 hour at RT and washed three times for 20 min and then cell nuclei were stained with 4′,6-diamidino-2-phenylindole (DAPI) for 15 min at RT. Sections were washed twice and mounted on glass slides in FluoroMount. A Leica Stellaris SP8 microscope was used to acquire images. The quantification of PV-1 intensity was performed using Fiji. Image processing was performed with Imaris Microscopy Imaging Software.

### Transmission electron microscopy

Tissue samples were fixed with 2% PFA and 2,5% glutaraldehyde in 0.1 M cacodylate buffer (pH 7.4) for 1 hour at RT and left overnight at 4°C in the same fixative. Tissues were then embedded in 3% low-gelling temperature agarose and sliced at 100 μm using a Leica VT1200S vibratome. Vibratome sections were then postfixed with reduced osmium solution [1% OsO_4_ and 1.5% potassium ferrocyanide in 0.1 M cacodylate buffer (pH 7.4)] for 2 hours on ice. After several washes in Milli-Q water, sections were incubated in 0.5% uranyl acetate overnight at 4°C. Samples were then dehydrated with increasing concentrations of ethanol embedded in epoxy resin and polymerized for 48 hours at 60°C between two plastic acetate sheets. Ultrathin sections were obtained using a Leica UC7 Ultramicrotome, collected on copper or nickel grids, stained with uranyl acetate and Sato’s lead solutions, and observed in a transmission electron microscope Talos L120C (FEI, Thermo Fisher Scientific) operating at 120 kV. Images were acquired with a Ceta charge-coupled device camera (FEI, Thermo Fisher Scientific). For quantification of microvilli and microvilli rootlets, images acquired at 4300x were quantified using Fiji software as previously described (*40*).

### IEC isolation, RNA extraction, and transcriptomic analysis

IECs were isolated by using a previously described method (*83*). Total RNA was extracted from IECs using the RNeasy Micro Kit and then digested with DNase I at 37°C for 15 min to remove any contaminating DNA. The NEBNext Ultra Directional RNA Library Prep Kit for Illumina (New England BioLabs Inc.) was used with the NEBNext Multiplex Oligos for Illumina (New England BioLabs Inc.) and NEBNext rRNA Depletion Kit v2 for cDNA synthesis with the addition of barcode sequences. The sequencing of the pre-pools was performed using the NextSeq2000 (Illumina) with the P2 reagents kit V3 (Illumina). Samples were processed starting from stranded, single-ended 120-bp-long sequencing reads. We used FastQC v0.11.9 (https://bioinformatics.babraham.ac.uk/projects/fastqc/) to assess the quality of the reads. Adapter sequences were removed using Trimmomatic v0.39. Reads were subsequently mapped to the mouse genome using HISAT2 v2.1.0. Reference genome sequences were retrieved from GENCODE (version GRCm39). We then used HTSeq-count v2.0.2 to generate the table of counts containing the number of reads mapping to each feature in each sequencing sample. We performed differential gene expression analysis and gene set enrichment analysis with DESeq2 and fgsea package, respectively.

### Histopathology and scoring of murine samples

Colon samples were rolled into a Swiss roll configuration and fixed in 10% neutral-buffered formalin. Tissues were routinely processed into paraffin blocks, sectioned at ~3 μm thickness, and stained with hematoxylin and eosin (H&E). Histological architecture and cytomorphological features were assessed and scored by a board-certified veterinary pathologist using a semiquantitative scale described by Zhou *et al.* (*49*), ranging from 0 (no change/lesion) to 4 (severe alteration/tissue destruction). Evaluated parameters included inflammatory infiltrates (the presence of neutrophils or mixed leukocytes in the lamina propria or submucosa), crypt hyperplasia (characterized by mitotic figures above the proliferative zone, nuclear pseudostratification, distorted crypt architecture, and goblet cell depletion), and epithelial damage. A cumulative histopathological score was then calculated by summing all individual parameter scores. In a second step, colon samples were stained with combined periodic acid–Schiff (PAS)/Alcian blue according to standard laboratory protocols.

### Photoconversion procedures

Photoconversion of PPs of KikGR mice was performed as described previously (*63*). Briefly, KikGR mice were anesthetized with isoflurane and the first three PPs starting from the distal part of ileum were exposed to violet light for 2.5 min (65 mW/cm^2^ at 5 mm of distance using UV Curing LED Systems with a 385-nm band-pass filter from ThorLabs). After the procedure, mice were monitored until full recovery from anesthesia, then every 2 hours for 6 hours after surgery, and then twice a day until the end of the experiment.

### CRISPR-Cas9 of CCL25 in MC38 cells

MC38 cells were electroporated with a pSpCas9(BB)-2A-GFP plasmid (PX458, Addgene #48138) in which guide RNAs (gRNAs) targeting different genes were inserted by Golden Gate cloning (forward, CACCGCATTCCATTTGATCCTGTGC; CCL25 reverse, AAACGCACAGGATCAAATGGAATGC). Using the Neon Transfection System (Invitrogen), 15 μg of plasmid was electroporated into 5 × 10^6^ cells in 100-μl transfection tips using the following settings: 1550 V, three pulses, and 10 ms. Control cells were electroporated with a PX458 vector containing a nontargeting gRNA sequence. After electroporation, cells were resuspended in complete RPMI supplemented with 10% heat-inactivated FBS, penicillin/streptomycin (100 U/ml), and kanamycin (100 U/ml). The next day, GFP^high^ cells were sorted and expanded in complete RPMI supplemented with 10% heat-inactivated FBS, penicillin/streptomycin (100 U/ml), and kanamycin (100 U/ml). Gene editing efficiency was analyzed by FACS analysis.

### Assessment of CCL25 expression in MC38 cells

MC38^sgCtrl^ or MC38s^gCCL25^ cells were subcutaneously injected into C57BL/6 mice (8 weeks old). After 15 days, the tumor was harvested, and cells were processed to obtain a single-cell suspension. Cells were stained with an anti-mCCL25/TECK conjugated to goat IgG (R&D Systems); the goat IgG was then bound by a biotin-conjugated anti-goat IgG antibody (Vector Laboratories). Last, APC conjugated to streptavidin (BioLegend) was used to stain the sample. As negative controls, MC38sgCtrl cells were stained only with the secondary and tertiary antibody. Samples were acquired using a BD FACSymphony flow cytometer (BD Bioscience), and data were analyzed using FlowJo v10.7 software (TreeStar).

### Human blood sample collection and cell sorting

The study was approved by the Ethical committees of Cantone Ticino, Switzerland (ref. 2018-02166/CE 3428). Blood from healthy donors was obtained from the Swiss Blood Donation Center of Lugano. All blood donors provided written informed consent for participation in the study. Peripheral blood mononuclear cells (PBMCs) were isolated with Ficoll-Paque Plus (GE Healthcare). Total CD8 T cells were isolated by positive selection using anti-CD8 magnetic microbeads (Miltenyi Biotech). Total CD8^+^ cells obtained by positive selection were stained for chemokine receptor CCR9 and CCR7 at 37°C and on ice for additional markers. Effector CCR9^+^ or CCR9^−^ CD8 T cells were sorted with a FACSAria III (BD Biosciences) to over 98% purity by gating on CD3^+^CD8^+^CD4^−^CCR7^−^ cells.

### Human cancer analysis

Comparison of CCL25 expression [transcripts per million (TPM)] between human cancer tissue and normal tissues was realized using the Gene Expression Profiling Interactive Analysis (GEPIA) using data from The Cancer Genome Atlas (TCGA) and the Genotype-Tissue Expression (GTEx). For survival analysis, CCL25 expression data (TPM) of TCGA cancer samples were obtained from the Human Protein Atlas and clinical data were obtained from the Broad Institute TCGA Genome Data Analysis Center (GDAC). Overall survival curves were presented and examined with Kaplan-Meier survival analysis by log-rank methods. The expression levels of the *CCL25* gene in tumors from patients with advanced melanoma were obtained from the study conducted by Chen *et al.* (*84*) and from GSE91061 (*85*). To better represent CCL25 expression in the different group of patients, data from Chen *et al.* were used as provided in supplementary table S6a of ref. (*84*). For GSE91061 [ref. (*85*)], raw counts were normalized to log_2_ counts per million (CPM) using the cpm function from the edgeR package [version 4.0.16; (*86*)]. Log_2_ CCL25 expression was quantified in matched pretreatment and on-treatment tumor samples from patients undergoing anti–PD-1 therapy, and log_2_ expression ratios were calculated. Samples with undetectable CCL25 expression in both time points were excluded from further analysis. Log_2_ expression ratios were compared between responders (*n* = 22) and nonresponders (*n* = 16) using the Mann-Whitney *U* test and performed in GraphPad Prism (table S4). A *P* value of <0.05 was considered statistically significant. All analyses were conducted in R (version 4.3.2).

### Survival analyses

Survival analyses were performed using the cSurvival (v1.0.6) web tool (*87*) on the indicated TCGA datasets, using the default parameters, except for the number of permutations, which we increased to 1000. Analyses generated Kaplan-Meier curves representing DSS probability. A log-rank test was used to assess the statistical differences in the DSS between groups. The HR and its *P* value were calculated using the Cox proportional hazard (PH) model. To analyze the interaction between biomarkers (i.e., CD8A, CD8B, CCR9, and CCL25), the “No. of analysis” parameter was increased to 2. When evaluating the impact of CCR9 and CCL25 expression on the survival probability, to normalize for CD8^+^ T cell tumor infiltration, we included CD8A and CD8B genes in both the “Analysis #1” and “Analysis #2” gene lists. When we compared more than two groups of patients, the HR was calculated between the “high high” group versus all the other groups as a whole, whereas the Cox PH *P* value was calculated between both the “high_high” group versus all the other groups as a whole and any of the three groups.

### scRNA-seq data processing and quality control

We obtained processed scRNA-seq data of CD8^+^ T cells isolated from patients with melanoma from the NCBI GEO (GEO accession: GSE123139) (*64*). Preprocessed expression matrices were imported into Scanpy (v1.9.6) (*88*) as annotated data (AnnData) objects and concatenated, using the pd.concat function, into a single object for unified analysis. Each sample was annotated with information about anonymized patient ID, tissue of origin, disease stage, and treatment. Then, CD8^+^ T cells were selected on the basis of the expression of CD3D, CD3E, CD3G, CD8A, and CD8B genes. Last, CCR9 expression in the different patients’ groups was visualized using the sc.tl.dotplot function using the following parameters: dot_max = 0.01, dot_min = 0, log = True, and standard_scale = ‘var’.

### Statistical analyses

All statistical analyses were performed using the statistical programming environment R version 4.0.3 (Team, 2017) or GraphPad Prism v7.04 (GraphPad Software, La Jolla, CA, USA). Statistically significant differences in the relative abundance of ASVs between groups were performed by the Wald test using FDR (false discovery rate) *P* value correction following DESeq2 read count normalization. Statistical significance was set at *P* < 0.05 (* at *P* < 0.05; ** at *P* < 0.01; *** at *P* < 0.001; **** at *P* < 0.0001). The mean differences with 0.05 < *P* < 0.10 were accepted as trends. The Mann-Whitney signed-rank test or one-way analysis of variance (ANOVA) was used for statistical evaluations as indicated in each experiment. For tumor growth curves, significance was determined via two-way ANOVA. For mouse survival studies, the Mantel-Cox log-rank test was used to evaluate statistical differences in Kaplan-Meier analysis. For analysis of contingency tables, Fisher’s test was used.

## References

[1] L. Galluzzi, E. Vacchelli, J. M. Bravo-San Pedro, A. Buque, L. Senovilla, E. E. Baracco, N. Bloy, F. Castoldi, J. P. Abastado, P. Agostinis, R. N. Apte, F. Aranda, M. Ayyoub, P. Beckhove, J. Y. Blay, L. Bracci, A. Caignard, C. Castelli, F. Cavallo, E. Celis, V. Cerundolo, A. Clayton, M. P. Colombo, L. Coussens, M. V. Dhodapkar, A. M. Eggermont, D. T. Fearon, W. H. Fridman, J. Fucikova, D. I. Gabrilovich, J. Galon, A. Garg, F. Ghiringhelli, G. Giaccone, E. Gilboa, S. Gnjatic, A. Hoos, A. Hosmalin, D. Jager, P. Kalinski, K. Karre, O. Kepp, R. Kiessling, J. M. Kirkwood, E. Klein, A. Knuth, C. E. Lewis, R. Liblau, M. T. Lotze, E. Lugli, J. P. Mach, F. Mattei, D. Mavilio, I. Melero, C. J. Melief, E. A. Mittendorf, L. Moretta, A. Odunsi, H. Okada, A. K. Palucka, M. E. Peter, K. J. Pienta, A. Porgador, G. C. Prendergast, G. A. Rabinovich, N. P. Restifo, N. Rizvi, C. Sautes-Fridman, H. Schreiber, B. Seliger, H. Shiku, B. Silva-Santos, M. J. Smyth, D. E. Speiser, R. Spisek, P. K. Srivastava, J. E. Talmadge, E. Tartour, S. H. Van Der Burg, B. J. Van Den Eynde, R. Vile, H. Wagner, J. S. Weber, T. L. Whiteside, J. D. Wolchok, L. Zitvogel, W. Zou, G. Kroemer, Classification of current anticancer immunotherapies. Oncotarget 5, 12472–12508 (2014). 25537519 10.18632/oncotarget.2998 PMC4350348

[2] P. Sharma, S. Goswami, D. Raychaudhuri, B. A. Siddiqui, P. Singh, A. Nagarajan, J. Liu, S. K. Subudhi, C. Poon, K. L. Gant, S. M. Herbrich, S. Anandhan, S. Islam, M. Amit, G. Anandappa, J. P. Allison, Immune checkpoint therapy-current perspectives and future directions. Cell 186, 1652–1669 (2023). 37059068 10.1016/j.cell.2023.03.006

[3] F. Sommer, F. Backhed, The gut microbiota—Masters of host development and physiology. Nat. Rev. Microbiol. 11, 227–238 (2013). 23435359 10.1038/nrmicro2974

[4] Y. Fan, O. Pedersen, Gut microbiota in human metabolic health and disease. Nat. Rev. Microbiol. 19, 55–71 (2021). 32887946 10.1038/s41579-020-0433-9

[5] Z. Jie, H. Xia, S. L. Zhong, Q. Feng, S. Li, S. Liang, H. Zhong, Z. Liu, Y. Gao, H. Zhao, D. Zhang, Z. Su, Z. Fang, Z. Lan, J. Li, L. Xiao, J. Li, R. Li, X. Li, F. Li, H. Ren, Y. Huang, Y. Peng, G. Li, B. Wen, B. Dong, J. Y. Chen, Q. S. Geng, Z. W. Zhang, H. Yang, J. Wang, J. Wang, X. Zhang, L. Madsen, S. Brix, G. Ning, X. Xu, X. Liu, Y. Hou, H. Jia, K. He, K. Kristiansen, The gut microbiome in atherosclerotic cardiovascular disease. Nat. Commun. 8, 845 (2017). 29018189 10.1038/s41467-017-00900-1 PMC5635030

[6] R. Wu, R. Xiong, Y. Li, J. Chen, R. Yan, Gut microbiome, metabolome, host immunity associated with inflammatory bowel disease and intervention of fecal microbiota transplantation. J. Autoimmun. 141, 103062 (2023). 37246133 10.1016/j.jaut.2023.103062

[7] V. Gopalakrishnan, C. N. Spencer, L. Nezi, A. Reuben, M. C. Andrews, T. V. Karpinets, P. A. Prieto, D. Vicente, K. Hoffman, S. C. Wei, A. P. Cogdill, L. Zhao, C. W. Hudgens, D. S. Hutchinson, T. Manzo, M. P. de Macedo, T. Cotechini, T. Kumar, W. S. Chen, S. M. Reddy, R. S. Sloane, J. Galloway-Pena, H. Jiang, P. L. Chen, E. J. Shpall, K. Rezvani, A. M. Alousi, R. F. Chemaly, S. Shelburne, L. M. Vence, P. C. Okhuysen, V. B. Jensen, A. G. Swennes, F. McAllister, E. M. R. Sanchez, Y. Zhang, E. Le Chatelier, L. Zitvogel, N. Pons, J. L. Austin-Breneman, L. E. Haydu, E. M. Burton, J. M. Gardner, E. Sirmans, J. Hu, A. J. Lazar, T. Tsujikawa, A. Diab, H. Tawbi, I. C. Glitza, W. J. Hwu, S. P. Patel, S. E. Woodman, R. N. Amaria, M. A. Davies, J. E. Gershenwald, P. Hwu, J. E. Lee, J. Zhang, L. M. Coussens, Z. A. Cooper, P. A. Futreal, C. R. Daniel, N. J. Ajami, J. F. Petrosino, M. T. Tetzlaff, P. Sharma, J. P. Allison, R. R. Jenq, J. A. Wargo, Gut microbiome modulates response to anti-PD-1 immunotherapy in melanoma patients. Science 359, 97–103 (2018). 29097493 10.1126/science.aan4236 PMC5827966

[8] L. F. Mager, R. Burkhard, N. Pett, N. C. A. Cooke, K. Brown, H. Ramay, S. Paik, J. Stagg, R. A. Groves, M. Gallo, I. A. Lewis, M. B. Geuking, K. D. McCoy, Microbiome-derived inosine modulates response to checkpoint inhibitor immunotherapy. Science 369, 1481–1489 (2020). 32792462 10.1126/science.abc3421

[9] V. Matson, J. Fessler, R. Bao, T. Chongsuwat, Y. Zha, M. L. Alegre, J. J. Luke, T. F. Gajewski, The commensal microbiome is associated with anti-PD-1 efficacy in metastatic melanoma patients. Science 359, 104–108 (2018). 29302014 10.1126/science.aao3290 PMC6707353

[10] B. Routy, E. Le Chatelier, L. Derosa, C. P. M. Duong, M. T. Alou, R. Daillere, A. Fluckiger, M. Messaoudene, C. Rauber, M. P. Roberti, M. Fidelle, C. Flament, V. Poirier-Colame, P. Opolon, C. Klein, K. Iribarren, L. Mondragon, N. Jacquelot, B. Qu, G. Ferrere, C. Clemenson, L. Mezquita, J. R. Masip, C. Naltet, S. Brosseau, C. Kaderbhai, C. Richard, H. Rizvi, F. Levenez, N. Galleron, B. Quinquis, N. Pons, B. Ryffel, V. Minard-Colin, P. Gonin, J. C. Soria, E. Deutsch, Y. Loriot, F. Ghiringhelli, G. Zalcman, F. Goldwasser, B. Escudier, M. D. Hellmann, A. Eggermont, D. Raoult, L. Albiges, G. Kroemer, L. Zitvogel, Gut microbiome influences efficacy of PD-1-based immunotherapy against epithelial tumors. Science 359, 91–97 (2018). 29097494 10.1126/science.aan3706

[11] A. Sivan, L. Corrales, N. Hubert, J. B. Williams, K. Aquino-Michaels, Z. M. Earley, F. W. Benyamin, Y. M. Lei, B. Jabri, M. L. Alegre, E. B. Chang, T. F. Gajewski, Commensal Bifidobacterium promotes antitumor immunity and facilitates anti-PD-L1 efficacy. Science 350, 1084–1089 (2015). 26541606 10.1126/science.aac4255 PMC4873287

[12] M. Vetizou, J. M. Pitt, R. Daillere, P. Lepage, N. Waldschmitt, C. Flament, S. Rusakiewicz, B. Routy, M. P. Roberti, C. P. Duong, V. Poirier-Colame, A. Roux, S. Becharef, S. Formenti, E. Golden, S. Cording, G. Eberl, A. Schlitzer, F. Ginhoux, S. Mani, T. Yamazaki, N. Jacquelot, D. P. Enot, M. Berard, J. Nigou, P. Opolon, A. Eggermont, P. L. Woerther, E. Chachaty, N. Chaput, C. Robert, C. Mateus, G. Kroemer, D. Raoult, I. G. Boneca, F. Carbonnel, M. Chamaillard, L. Zitvogel, Anticancer immunotherapy by CTLA-4 blockade relies on the gut microbiota. Science 350, 1079–1084 (2015). 26541610 10.1126/science.aad1329 PMC4721659

[13] A. Elkrief, L. Derosa, G. Kroemer, L. Zitvogel, B. Routy, The negative impact of antibiotics on outcomes in cancer patients treated with immunotherapy: A new independent prognostic factor? Ann. Oncol. 30, 1572–1579 (2019). 31268133 10.1093/annonc/mdz206

[14] B. Di Luccia, M. Molgora, D. Khantakova, N. Jaeger, H. W. Chang, R. S. Czepielewski, B. A. Helmink, E. J. Onufer, J. L. Fachi, B. Bhattarai, T. Trsan, P. F. Rodrigues, J. Hou, J. K. Bando, C. S. da Silva, M. Cella, S. Gilfillan, R. D. Schreiber, J. I. Gordon, M. Colonna, TREM2 deficiency reprograms intestinal macrophages and microbiota to enhance anti-PD-1 tumor immunotherapy. Sci. Immunol. 9, eadi5374 (2024). 38758808 10.1126/sciimmunol.adi5374 PMC11299520

[15] M. Fidelle, C. Rauber, C. A. C. Silva, A. L. Tian, I. Lahmar, A. M. de La Varende, L. Zhao, C. Thelemaque, I. Lebhar, M. Messaoudene, E. Pizzato, R. Birebent, M. D. M. Fonkou, S. Zoppi, A. Reni, C. Dalban, M. Leduc, G. Ferrere, S. Durand, P. Ly, A. Silvin, K. Mulder, C. A. Dutertre, F. Ginhoux, S. Yonekura, M. P. Roberti, M. Tidjani-Alou, S. Terrisse, J. Chen, O. Kepp, A. Schippers, N. Wagner, J. Suarez-Gosalvez, S. Kobold, J. E. Fahrner, C. Richard, J. Bosq, L. Lordello, G. Vitali, N. Galleron, B. Quinquis, E. Le Chatelier, L. Blanchard, J. P. Girard, A. Jarry, N. Gervois, E. Godefroy, N. Labarriere, R. Koschny, R. Daillere, B. Besse, C. Truntzer, F. Ghiringhelli, N. Coatnoan, V. Mhanna, D. Klatzmann, D. Drubay, L. Albiges, A. M. Thomas, N. Segata, F. X. Danlos, A. Marabelle, B. Routy, L. Derosa, G. Kroemer, L. Zitvogel, A microbiota-modulated checkpoint directs immunosuppressive intestinal T cells into cancers. Science 380, eabo2296 (2023). 37289890 10.1126/science.abo2296

[16] E. N. Baruch, I. Youngster, G. Ben-Betzalel, R. Ortenberg, A. Lahat, L. Katz, K. Adler, D. Dick-Necula, S. Raskin, N. Bloch, D. Rotin, L. Anafi, C. Avivi, J. Melnichenko, Y. Steinberg-Silman, R. Mamtani, H. Harati, N. Asher, R. Shapira-Frommer, T. Brosh-Nissimov, Y. Eshet, S. Ben-Simon, O. Ziv, M. A. W. Khan, M. Amit, N. J. Ajami, I. Barshack, J. Schachter, J. A. Wargo, O. Koren, G. Markel, B. Boursi, Fecal microbiota transplant promotes response in immunotherapy-refractory melanoma patients. Science 371, 602–609 (2021). 33303685 10.1126/science.abb5920

[17] D. Davar, A. K. Dzutsev, J. A. McCulloch, R. R. Rodrigues, J. M. Chauvin, R. M. Morrison, R. N. Deblasio, C. Menna, Q. Ding, O. Pagliano, B. Zidi, S. Zhang, J. H. Badger, M. Vetizou, A. M. Cole, M. R. Fernandes, S. Prescott, R. G. F. Costa, A. K. Balaji, A. Morgun, I. Vujkovic-Cvijin, H. Wang, A. A. Borhani, M. B. Schwartz, H. M. Dubner, S. J. Ernst, A. Rose, Y. G. Najjar, Y. Belkaid, J. M. Kirkwood, G. Trinchieri, H. M. Zarour, Fecal microbiota transplant overcomes resistance to anti-PD-1 therapy in melanoma patients. Science 371, 595–602 (2021). 33542131 10.1126/science.abf3363 PMC8097968

[18] B. Routy, J. G. Lenehan, W. H. Miller Jr., R. Jamal, M. Messaoudene, B. A. Daisley, C. Hes, K. F. Al, L. Martinez-Gili, M. Puncochar, S. Ernst, D. Logan, K. Belanger, K. Esfahani, C. Richard, M. Ninkov, G. Piccinno, F. Armanini, F. Pinto, M. Krishnamoorthy, R. Figueredo, P. Thebault, P. Takis, J. Magrill, L. Ramsay, L. Derosa, J. R. Marchesi, S. N. Parvathy, A. Elkrief, I. R. Watson, R. Lapointe, N. Segata, S. M. M. Haeryfar, B. H. Mullish, M. S. Silverman, J. P. Burton, S. Maleki Vareki, Fecal microbiota transplantation plus anti-PD-1 immunotherapy in advanced melanoma: A phase I trial. Nat. Med. 29, 2121–2132 (2023). 37414899 10.1038/s41591-023-02453-x

[19] M. E. Johansson, M. Phillipson, J. Petersson, A. Velcich, L. Holm, G. C. Hansson, The inner of the two Muc2 mucin-dependent mucus layers in colon is devoid of bacteria. Proc. Natl. Acad. Sci. U.S.A. 105, 15064–15069 (2008). 18806221 10.1073/pnas.0803124105 PMC2567493

[20] E. W. Rogier, A. L. Frantz, M. E. Bruno, L. Wedlund, D. A. Cohen, A. J. Stromberg, C. S. Kaetzel, Secretory antibodies in breast milk promote long-term intestinal homeostasis by regulating the gut microbiota and host gene expression. Proc. Natl. Acad. Sci. U.S.A. 111, 3074–3079 (2014). 24569806 10.1073/pnas.1315792111 PMC3939878

[21] L. Perruzza, F. Strati, G. Gargari, A. M. D’Erchia, B. Fosso, G. Pesole, S. Guglielmetti, F. Grassi, Enrichment of intestinal Lactobacillus by enhanced secretory IgA coating alters glucose homeostasis in P2rx7^−/−^ mice. Sci. Rep. 9, 9315 (2019). 31249344 10.1038/s41598-019-45724-9 PMC6597561

[22] S. Fagarasan, T. Honjo, Intestinal IgA synthesis: Regulation of front-line body defences. Nat. Rev. Immunol. 3, 63–72 (2003). 12511876 10.1038/nri982

[23] M. Proietti, V. Cornacchione, T. Rezzonico Jost, A. Romagnani, C. E. Faliti, L. Perruzza, R. Rigoni, E. Radaelli, F. Caprioli, S. Preziuso, B. Brannetti, M. Thelen, K. D. McCoy, E. Slack, E. Traggiai, F. Grassi, ATP-gated ionotropic P2X7 receptor controls follicular T helper cell numbers in Peyer’s patches to promote host-microbiota mutualism. Immunity 41, 789–801 (2014). 25464855 10.1016/j.immuni.2014.10.010

[24] L. Perruzza, G. Gargari, M. Proietti, B. Fosso, A. M. D’Erchia, C. E. Faliti, T. Rezzonico-Jost, D. Scribano, L. Mauri, D. Colombo, G. Pellegrini, A. Moregola, C. Mooser, G. Pesole, M. Nicoletti, G. D. Norata, M. B. Geuking, K. D. McCoy, S. Guglielmetti, F. Grassi, T follicular helper cells promote a beneficial gut ecosystem for host metabolic homeostasis by sensing microbiota-derived extracellular ATP. Cell Rep. 18, 2566–2575 (2017). 28297661 10.1016/j.celrep.2017.02.061 PMC5368345

[25] M. Proietti, L. Perruzza, D. Scribano, G. Pellegrini, R. D’Antuono, F. Strati, M. Raffaelli, S. F. Gonzalez, M. Thelen, W. D. Hardt, E. Slack, M. Nicoletti, F. Grassi, ATP released by intestinal bacteria limits the generation of protective IgA against enteropathogens. Nat. Commun. 10, 250 (2019). 30651557 10.1038/s41467-018-08156-z PMC6335424

[26] L. Perruzza, F. Strati, M. Raneri, H. Li, G. Gargari, T. Rezzonico-Jost, M. Palatella, I. Kwee, D. Morone, F. Seehusen, P. Sonego, C. Donati, P. Franceschi, A. J. Macpherson, S. Guglielmetti, V. Greiff, F. Grassi, Apyrase-mediated amplification of secretory IgA promotes intestinal homeostasis. Cell Rep. 40, 111112 (2022). 35858559 10.1016/j.celrep.2022.111112

[27] O. Pabst, E. Slack, IgA and the intestinal microbiota: The importance of being specific. Mucosal Immunol. 13, 12–21 (2020). 31740744 10.1038/s41385-019-0227-4 PMC6914667

[28] Y. Uchimura, T. Fuhrer, H. Li, M. A. Lawson, M. Zimmermann, B. Yilmaz, J. Zindel, F. Ronchi, M. Sorribas, S. Hapfelmeier, S. C. Ganal-Vonarburg, M. Gomez de Aguero, K. D. McCoy, U. Sauer, A. J. Macpherson, Antibodies set boundaries limiting microbial metabolite penetration and the resultant mammalian host response. Immunity 49, 545–559.e5 (2018). 30193848 10.1016/j.immuni.2018.08.004 PMC6162337

[29] P. E. Conrey, L. Denu, K. C. O’Boyle, I. Rozich, J. Green, J. Maslanka, J. B. Lubin, T. Duranova, B. L. Haltzman, L. Gianchetti, D. A. Oldridge, N. De Luna, L. A. Vella, D. Allman, J. M. Spergel, C. Tanes, K. Bittinger, S. E. Henrickson, M. A. Silverman, IgA deficiency destabilizes homeostasis toward intestinal microbes and increases systemic immune dysregulation. Sci. Immunol. 8, eade2335 (2023). 37235682 10.1126/sciimmunol.ade2335 PMC11623094

[30] D. Scribano, A. Petrucca, M. Pompili, C. Ambrosi, E. Bruni, C. Zagaglia, G. Prosseda, L. Nencioni, M. Casalino, F. Polticelli, M. Nicoletti, Polar localization of PhoN2, a periplasmic virulence-associated factor of Shigella flexneri, is required for proper IcsA exposition at the old bacterial pole. PLOS ONE 9, e90230 (2014). 24587292 10.1371/journal.pone.0090230 PMC3937361

[31] F. P. Canale, C. Basso, G. Antonini, M. Perotti, N. Li, A. Sokolovska, J. Neumann, M. J. James, S. Geiger, W. Jin, J. P. Theurillat, K. A. West, D. S. Leventhal, J. M. Lora, F. Sallusto, R. Geiger, Metabolic modulation of tumours with engineered bacteria for immunotherapy. Nature 598, 662–666 (2021). 34616044 10.1038/s41586-021-04003-2

[32] E. De Marchi, E. Orioli, A. Pegoraro, S. Sangaletti, P. Portararo, A. Curti, M. P. Colombo, F. Di Virgilio, E. Adinolfi, The P2X7 receptor modulates immune cells infiltration, ectonucleotidases expression and extracellular ATP levels in the tumor microenvironment. Oncogene 38, 3636–3650 (2019). 30655604 10.1038/s41388-019-0684-y PMC6756114

[33] F. Fransen, E. Zagato, E. Mazzini, B. Fosso, C. Manzari, S. El Aidy, A. Chiavelli, A. M. D’Erchia, M. K. Sethi, O. Pabst, M. Marzano, S. Moretti, L. Romani, G. Penna, G. Pesole, M. Rescigno, BALB/c and C57BL/6 mice differ in polyreactive IgA abundance, which impacts the generation of antigen-specific IgA and microbiota diversity. Immunity 43, 527–540 (2015). 26362264 10.1016/j.immuni.2015.08.011

[34] M. T. Chow, A. J. Ozga, R. L. Servis, D. T. Frederick, J. A. Lo, D. E. Fisher, G. J. Freeman, G. M. Boland, A. D. Luster, Intratumoral activity of the CXCR3 chemokine system is required for the efficacy of anti-PD-1 therapy. Immunity 50, 1498–1512.e5 (2019). 31097342 10.1016/j.immuni.2019.04.010 PMC6527362

[35] M. Gohda, J. Kunisawa, F. Miura, Y. Kagiyama, Y. Kurashima, M. Higuchi, I. Ishikawa, I. Ogahara, H. Kiyono, Sphingosine 1-phosphate regulates the egress of IgA plasmablasts from Peyer’s patches for intestinal IgA responses. J. Immunol. 180, 5335–5343 (2008). 18390715 10.4049/jimmunol.180.8.5335

[36] S. Nicolaides, A. Boussioutas, Immune-related adverse events of the gastrointestinal system. Cancers 15, 691 (2023). 36765649 10.3390/cancers15030691 PMC9913287

[37] N. G. Nunez, F. Berner, E. Friebel, S. Unger, N. Wyss, J. M. Gomez, M. T. Purde, R. Niederer, M. Porsch, C. Lichtensteiger, R. Kramer, M. Erdmann, C. Schmitt, L. Heinzerling, M. T. Abdou, J. Karbach, D. Schadendorf, L. Zimmer, S. Ugurel, N. Klumper, M. Holzel, L. Power, S. Kreutmair, M. Capone, G. Madonna, L. Cevhertas, A. Heider, T. Amaral, O. Hasan Ali, D. Bomze, F. Dimitriou, S. Diem, P. A. Ascierto, R. Dummer, E. Jager, C. Driessen, M. P. Levesque, W. van de Veen, M. Joerger, M. Fruh, B. Becher, L. Flatz, Immune signatures predict development of autoimmune toxicity in patients with cancer treated with immune checkpoint inhibitors. Med 4, 113–129.e7 (2023). 36693381 10.1016/j.medj.2022.12.007

[38] A. Bertocchi, S. Carloni, P. S. Ravenda, G. Bertalot, I. Spadoni, A. Lo Cascio, S. Gandini, M. Lizier, D. Braga, F. Asnicar, N. Segata, C. Klaver, P. Brescia, E. Rossi, A. Anselmo, S. Guglietta, A. Maroli, P. Spaggiari, N. Tarazona, A. Cervantes, S. Marsoni, L. Lazzari, M. G. Jodice, C. Luise, M. Erreni, S. Pece, P. P. Di Fiore, G. Viale, A. Spinelli, C. Pozzi, G. Penna, M. Rescigno, Gut vascular barrier impairment leads to intestinal bacteria dissemination and colorectal cancer metastasis to liver. Cancer Cell 39, 708–724.e11 (2021). 33798472 10.1016/j.ccell.2021.03.004

[39] I. Spadoni, E. Zagato, A. Bertocchi, R. Paolinelli, E. Hot, A. Di Sabatino, F. Caprioli, L. Bottiglieri, A. Oldani, G. Viale, G. Penna, E. Dejana, M. Rescigno, A gut-vascular barrier controls the systemic dissemination of bacteria. Science 350, 830–834 (2015). 26564856 10.1126/science.aad0135

[40] K. L. VanDussen, A. Stojmirovic, K. Li, T. C. Liu, P. K. Kimes, B. D. Muegge, K. F. Simpson, M. A. Ciorba, J. G. Perrigoue, J. R. Friedman, J. E. Towne, R. D. Head, T. S. Stappenbeck, Abnormal small intestinal epithelial microvilli in patients with Crohn’s disease. Gastroenterology 155, 815–828 (2018). 29782846 10.1053/j.gastro.2018.05.028 PMC6378688

[41] H. H. Al-Numan, R. M. Jan, N. B. S. Al-Saud, O. M. Rashidi, N. M. Alrayes, H. A. Alsufyani, A. Mujalli, N. A. Shaik, M. H. Mosli, R. Elango, O. I. Saadah, B. Banaganapalli, Exome sequencing identifies the extremely rare *ITGAV* and *FN1* variants in early onset inflammatory bowel disease patients. Front. Pediatr. 10, 895074 (2022). 35692981 10.3389/fped.2022.895074 PMC9178107

[42] D. M. Alvarado, B. Chen, M. Iticovici, A. I. Thaker, N. Dai, K. L. VanDussen, N. Shaikh, C. K. Lim, G. J. Guillemin, P. I. Tarr, M. A. Ciorba, Epithelial indoleamine 2,3-dioxygenase 1 modulates aryl hydrocarbon receptor and notch signaling to increase differentiation of secretory cells and alter mucus-associated microbiota. Gastroenterology 157, 1093–1108.e11 (2019). 31325428 10.1053/j.gastro.2019.07.013 PMC6756966

[43] S. Ashizuka, K. Inagaki-Ohara, K. Kuwasako, J. Kato, H. Inatsu, K. Kitamura, Adrenomedullin treatment reduces intestinal inflammation and maintains epithelial barrier function in mice administered dextran sulphate sodium. Microbiol. Immunol. 53, 573–581 (2009). 19780971 10.1111/j.1348-0421.2009.00159.x

[44] K. A. Knoop, P. E. Coughlin, A. N. Floyd, I. M. Ndao, C. Hall-Moore, N. Shaikh, A. J. Gasparrini, B. Rusconi, M. Escobedo, M. Good, B. B. Warner, P. I. Tarr, R. D. Newberry, Maternal activation of the EGFR prevents translocation of gut-residing pathogenic Escherichia coli in a model of late-onset neonatal sepsis. Proc. Natl. Acad. Sci. U.S.A. 117, 7941–7949 (2020). 32179676 10.1073/pnas.1912022117 PMC7148560

[45] C. C. Leow, M. S. Romero, S. Ross, P. Polakis, W. Q. Gao, Hath1, down-regulated in colon adenocarcinomas, inhibits proliferation and tumorigenesis of colon cancer cells. Cancer Res. 64, 6050–6057 (2004). 15342386 10.1158/0008-5472.CAN-04-0290

[46] G. Lorden, I. Sanjuan-Garcia, N. de Pablo, C. Meana, I. Alvarez-Miguel, M. T. Perez-Garcia, P. Pelegrin, J. Balsinde, M. A. Balboa, Lipin-2 regulates NLRP3 inflammasome by affecting P2X7 receptor activation. J. Exp. Med. 214, 511–528 (2017). 28031477 10.1084/jem.20161452 PMC5294860

[47] M. C. Ryan, K. Lee, Y. Miyashita, W. G. Carter, Targeted disruption of the LAMA3 gene in mice reveals abnormalities in survival and late stage differentiation of epithelial cells. J. Cell Biol. 145, 1309–1324 (1999). 10366601 10.1083/jcb.145.6.1309 PMC2133157

[48] L. Zhu, J. Han, L. Li, Y. Wang, Y. Li, S. Zhang, Claudin family participates in the pathogenesis of inflammatory bowel diseases and colitis-associated colorectal cancer. Front. Immunol. 10, 1441 (2019). 31316506 10.3389/fimmu.2019.01441 PMC6610251

[49] Y. Zhou, Y. B. Medik, B. Patel, D. B. Zamler, S. Chen, T. Chapman, S. Schneider, E. M. Park, R. L. Babcock, T. T. Chrisikos, L. M. Kahn, A. M. Dyevoich, J. E. Pineda, M. C. Wong, A. K. Mishra, S. H. Cass, A. P. Cogdill, D. H. Johnson, S. B. Johnson, K. Wani, D. A. Ledesma, C. W. Hudgens, J. Wang, M. A. Wadud Khan, C. B. Peterson, A. Y. Joon, W. Peng, H. S. Li, R. Arora, X. Tang, M. G. Raso, X. Zhang, W. C. Foo, M. T. Tetzlaff, G. E. Diehl, K. Clise-Dwyer, E. M. Whitley, M. M. Gubin, J. P. Allison, P. Hwu, N. J. Ajami, A. Diab, J. A. Wargo, S. S. Watowich, Intestinal toxicity to CTLA-4 blockade driven by IL-6 and myeloid infiltration. J. Exp. Med. 220, e20221333 (2023). 36367776 10.1084/jem.20221333 PMC9664499

[50] K. E. Lewis, M. J. Selby, G. Masters, J. Valle, G. Dito, W. R. Curtis, R. Garcia, K. A. Mink, K. S. Waggie, M. S. Holdren, J. F. Grosso, A. J. Korman, M. Jure-Kunkel, S. R. Dillon, Interleukin-21 combined with PD-1 or CTLA-4 blockade enhances antitumor immunity in mouse tumor models. Oncoimmunology 7, e1377873 (2017). 29296539 10.1080/2162402X.2017.1377873 PMC5739581

[51] S. J. Im, M. Hashimoto, M. Y. Gerner, J. Lee, H. T. Kissick, M. C. Burger, Q. Shan, J. S. Hale, J. Lee, T. H. Nasti, A. H. Sharpe, G. J. Freeman, R. N. Germain, H. I. Nakaya, H. H. Xue, R. Ahmed, Defining CD8^+^ T cells that provide the proliferative burst after PD-1 therapy. Nature 537, 417–421 (2016). 27501248 10.1038/nature19330 PMC5297183

[52] B. C. Miller, D. R. Sen, R. Al Abosy, K. Bi, Y. V. Virkud, M. W. LaFleur, K. B. Yates, A. Lako, K. Felt, G. S. Naik, M. Manos, E. Gjini, J. R. Kuchroo, J. J. Ishizuka, J. L. Collier, G. K. Griffin, S. Maleri, D. E. Comstock, S. A. Weiss, F. D. Brown, A. Panda, M. D. Zimmer, R. T. Manguso, F. S. Hodi, S. J. Rodig, A. H. Sharpe, W. N. Haining, Subsets of exhausted CD8^+^ T cells differentially mediate tumor control and respond to checkpoint blockade. Nat. Immunol. 20, 326–336 (2019). 30778252 10.1038/s41590-019-0312-6 PMC6673650

[53] M. Singer, C. Wang, L. Cong, N. D. Marjanovic, M. S. Kowalczyk, H. Zhang, J. Nyman, K. Sakuishi, S. Kurtulus, D. Gennert, J. Xia, J. Y. H. Kwon, J. Nevin, R. H. Herbst, I. Yanai, O. Rozenblatt-Rosen, V. K. Kuchroo, A. Regev, A. C. Anderson, A distinct gene module for dysfunction uncoupled from activation in tumor-infiltrating T cells. Cell 166, 1500–1511.e9 (2016). 27610572 10.1016/j.cell.2016.08.052 PMC5019125

[54] W. H. Hudson, J. Gensheimer, M. Hashimoto, A. Wieland, R. M. Valanparambil, P. Li, J. X. Lin, B. T. Konieczny, S. J. Im, G. J. Freeman, W. J. Leonard, H. T. Kissick, R. Ahmed, Proliferating transitory T cells with an effector-like transcriptional signature emerge from PD-1^+^ stem-like CD8^+^ T cells during chronic infection. Immunity 51, 1043–1058.e4 (2019). 31810882 10.1016/j.immuni.2019.11.002 PMC6920571

[55] M. Sade-Feldman, K. Yizhak, S. L. Bjorgaard, J. P. Ray, C. G. de Boer, R. W. Jenkins, D. J. Lieb, J. H. Chen, D. T. Frederick, M. Barzily-Rokni, S. S. Freeman, A. Reuben, P. J. Hoover, A. C. Villani, E. Ivanova, A. Portell, P. H. Lizotte, A. R. Aref, J. P. Eliane, M. R. Hammond, H. Vitzthum, S. M. Blackmon, B. Li, V. Gopalakrishnan, S. M. Reddy, Z. A. Cooper, C. P. Paweletz, D. A. Barbie, A. Stemmer-Rachamimov, K. T. Flaherty, J. A. Wargo, G. M. Boland, R. J. Sullivan, G. Getz, N. Hacohen, Defining T cell states associated with response to checkpoint immunotherapy in melanoma. Cell 175, 998–1013.e20 (2018). 30388456 10.1016/j.cell.2018.10.038 PMC6641984

[56] S. Fagarasan, S. Kawamoto, O. Kanagawa, K. Suzuki, Adaptive immune regulation in the gut: T cell-dependent and T cell-independent IgA synthesis. Annu. Rev. Immunol. 28, 243–273 (2010). 20192805 10.1146/annurev-immunol-030409-101314

[57] M. A. Jackson, C. Pearson, N. E. Ilott, K. E. Huus, A. N. Hegazy, J. Webber, B. B. Finlay, A. J. Macpherson, F. Powrie, L. H. Lam, Accurate identification and quantification of commensal microbiota bound by host immunoglobulins. Microbiome 9, 33 (2021). 33516266 10.1186/s40168-020-00992-w PMC7847592

[58] D. Jia, Q. Wang, Y. Qi, Y. Jiang, J. He, Y. Lin, Y. Sun, J. Xu, W. Chen, L. Fan, R. Yan, W. Zhang, G. Ren, C. Xu, Q. Ge, L. Wang, W. Liu, F. Xu, P. Wu, Y. Wang, S. Chen, L. Wang, Microbial metabolite enhances immunotherapy efficacy by modulating T cell stemness in pan-cancer. Cell 187, 1651–1665.e21 (2024). 38490195 10.1016/j.cell.2024.02.022

[59] H. Kawanabe-Matsuda, K. Takeda, M. Nakamura, S. Makino, T. Karasaki, K. Kakimi, M. Nishimukai, T. Ohno, J. Omi, K. Kano, A. Uwamizu, H. Yagita, I. G. Boneca, G. Eberl, J. Aoki, M. J. Smyth, K. Okumura, Dietary *Lactobacillus*-derived exopolysaccharide enhances immune-checkpoint blockade therapy. Cancer Discov. 12, 1336–1355 (2022). 35180303 10.1158/2159-8290.CD-21-0929 PMC9662940

[60] I. Campedelli, H. Mathur, E. Salvetti, S. Clarke, M. C. Rea, S. Torriani, R. P. Ross, C. Hill, P. W. O’Toole, Genus-wide assessment of antibiotic resistance in *Lactobacillus* spp. Appl. Environ. Microbiol. 85, e01738-18 (2019). 30366997 10.1128/AEM.01738-18 PMC6293106

[61] N. Jacquelot, D. P. Enot, C. Flament, N. Vimond, C. Blattner, J. M. Pitt, T. Yamazaki, M. P. Roberti, R. Daillere, M. Vetizou, V. Poirier-Colame, M. Semeraro, A. Caignard, C. L. Slingluff Jr., F. Sallusto, S. Rusakiewicz, B. Weide, A. Marabelle, H. Kohrt, S. Dalle, A. Cavalcanti, G. Kroemer, A. M. Di Giacomo, M. Maio, P. Wong, J. Yuan, J. Wolchok, V. Umansky, A. Eggermont, L. Zitvogel, Chemokine receptor patterns in lymphocytes mirror metastatic spreading in melanoma. J. Clin. Invest. 126, 921–937 (2016). 26854930 10.1172/JCI80071 PMC4767356

[62] A. D. G. Macandog, C. Catozzi, M. Capone, A. Nabinejad, P. P. Nanaware, S. Liu, S. Vinjamuri, J. A. Stunnenberg, S. Galie, M. G. Jodice, F. Montani, F. Armanini, E. Cassano, G. Madonna, D. Mallardo, B. Mazzi, S. Pece, M. Tagliamonte, V. Vanella, M. Barberis, P. F. Ferrucci, C. U. Blank, M. Bouvier, M. C. Andrews, X. Xu, L. Santambrogio, N. Segata, L. Buonaguro, E. Cocorocchio, P. A. Ascierto, T. Manzo, L. Nezi, Longitudinal analysis of the gut microbiota during anti-PD-1 therapy reveals stable microbial features of response in melanoma patients. Cell Host Microbe 32, 2004–2018.e9 (2024). 39481388 10.1016/j.chom.2024.10.006 PMC11629153

[63] M. Tomura, A. Hata, S. Matsuoka, F. H. Shand, Y. Nakanishi, R. Ikebuchi, S. Ueha, H. Tsutsui, K. Inaba, K. Matsushima, A. Miyawaki, K. Kabashima, T. Watanabe, O. Kanagawa, Tracking and quantification of dendritic cell migration and antigen trafficking between the skin and lymph nodes. Sci. Rep. 4, 6030 (2014). 25112380 10.1038/srep06030 PMC4129424

[64] H. Li, A. M. van der Leun, I. Yofe, Y. Lubling, D. Gelbard-Solodkin, A. C. J. van Akkooi, M. van den Braber, E. A. Rozeman, J. Haanen, C. U. Blank, H. M. Horlings, E. David, Y. Baran, A. Bercovich, A. Lifshitz, T. N. Schumacher, A. Tanay, I. Amit, Dysfunctional CD8 T cells form a proliferative, dynamically regulated compartment within human melanoma. Cell 176, 775–789.e18 (2019). 30595452 10.1016/j.cell.2018.11.043 PMC7253294

[65] E. M. Park, M. Chelvanambi, N. Bhutiani, G. Kroemer, L. Zitvogel, J. A. Wargo, Targeting the gut and tumor microbiota in cancer. Nat. Med. 28, 690–703 (2022). 35440726 10.1038/s41591-022-01779-2

[66] R. Goguyer-Deschaumes, L. Waeckel, M. Killian, N. Rochereau, S. Paul, Metabolites and secretory immunoglobulins: Messengers and effectors of the host-microbiota intestinal equilibrium. Trends Immunol. 43, 63–77 (2022). 34848167 10.1016/j.it.2021.11.005

[67] A. Biram, A. Stromberg, E. Winter, L. Stoler-Barak, R. Salomon, Y. Addadi, R. Dahan, G. Yaari, M. Bemark, Z. Shulman, BCR affinity differentially regulates colonization of the subepithelial dome and infiltration into germinal centers within Peyer’s patches. Nat. Immunol. 20, 482–492 (2019). 30833793 10.1038/s41590-019-0325-1

[68] K. J. Lee, D. Choi, N. Tae, H. W. Song, Y. W. Kang, M. Lee, D. Moon, Y. Oh, S. Park, J. H. Kim, S. Jeong, J. Yang, U. Park, D. H. Hong, M. S. Byun, S. H. Park, J. Sohn, Y. Park, S. K. Im, S. S. Choi, D. H. Kim, S. W. Lee, IL-7-primed bystander CD8 tumor-infiltrating lymphocytes optimize the antitumor efficacy of T cell engager immunotherapy. Cell Rep. Med. 5, 101567 (2024). 38744277 10.1016/j.xcrm.2024.101567 PMC11148861

[69] J. Liang, L. Zhu, J. Li, K. Wu, M. Zhang, S. Ma, X. Chen, B. Xia, Comprehensive analysis to identify IL7R as a immunotherapy biomarker from pan-cancer analysis to in vitro validation. Discov. Oncol. 15, 509 (2024). 39347891 10.1007/s12672-024-01357-7 PMC11442881

[70] G. Micevic, A. Daniels, K. Flem-Karlsen, K. Park, R. Talty, M. McGeary, H. Mirza, H. N. Blackburn, E. Sefik, J. F. Cheung, N. I. Hornick, L. Aizenbud, N. S. Joshi, H. Kluger, A. Iwasaki, M. W. Bosenberg, R. A. Flavell, IL-7R licenses a population of epigenetically poised memory CD8^+^ T cells with superior antitumor efficacy that are critical for melanoma memory. Proc. Natl. Acad. Sci. U.S.A. 120, e2304319120 (2023). 37459511 10.1073/pnas.2304319120 PMC10372654

[71] M. J. Bender, A. C. McPherson, C. M. Phelps, S. P. Pandey, C. R. Laughlin, J. H. Shapira, L. Medina Sanchez, M. Rana, T. G. Richie, T. S. Mims, A. M. Gocher-Demske, L. Cervantes-Barragan, S. J. Mullett, S. L. Gelhaus, T. C. Bruno, N. Cannon, J. A. McCulloch, D. A. A. Vignali, R. Hinterleitner, A. V. Joglekar, J. F. Pierre, S. T. M. Lee, D. Davar, H. M. Zarour, M. Meisel, Dietary tryptophan metabolite released by intratumoral *Lactobacillus reuteri* facilitates immune checkpoint inhibitor treatment. Cell 186, 1846–1862.e26 (2023). 37028428 10.1016/j.cell.2023.03.011 PMC10148916

[72] Y. Choi, J. N. Lichterman, L. A. Coughlin, N. Poulides, W. Li, P. Del Valle, S. N. Palmer, S. Gan, J. Kim, X. Zhan, Y. Gao, B. M. Evers, L. V. Hooper, C. Pasare, A. Y. Koh, Immune checkpoint blockade induces gut microbiota translocation that augments extraintestinal antitumor immunity. Sci. Immunol. 8, eabo2003 (2023). 36867675 10.1126/sciimmunol.abo2003 PMC10080670

[73] J. R. Bjork, L. A. Bolte, A. Maltez Thomas, K. A. Lee, N. Rossi, T. T. Wind, L. M. Smit, F. Armanini, F. Asnicar, A. Blanco-Miguez, R. Board, N. Calbet-Llopart, L. Derosa, N. Dhomen, K. Brooks, M. Harland, M. Harries, P. Lorigan, P. Manghi, R. Marais, J. Newton-Bishop, L. Nezi, F. Pinto, M. Potrony, S. Puig, P. Serra-Bellver, H. M. Shaw, S. Tamburini, S. Valpione, L. Waldron, L. Zitvogel, M. Zolfo, E. G. E. de Vries, P. Nathan, R. S. N. Fehrmann, T. D. Spector, V. Bataille, N. Segata, G. A. P. Hospers, R. K. Weersma, Longitudinal gut microbiome changes in immune checkpoint blockade-treated advanced melanoma. Nat. Med. 30, 785–796 (2024). 38365950 10.1038/s41591-024-02803-3 PMC10957474

[74] S. Yonekura, S. Terrisse, C. A. C. Silva, A. Lafarge, V. Iebba, G. Ferrere, A. G. Goubet, J. E. Fahrner, I. Lahmar, K. Ueda, G. Mansouri, E. Pizzato, P. Ly, M. Mazzenga, C. Thelemaque, M. Fidelle, F. Jaulin, J. Cartry, M. Deloger, M. Aglave, N. Droin, P. Opolon, A. Puget, F. Mann, M. Neunlist, A. Bessard, L. Aymeric, T. Matysiak-Budnik, J. Bosq, P. Hofman, C. P. M. Duong, S. Ugolini, V. Quiniou, S. Berrard, B. Ryffel, O. Kepp, G. Kroemer, B. Routy, L. Lordello, M. A. Bani, N. Segata, F. Y. Yengej, H. Clevers, J. Y. Scoazec, E. Pasolli, L. Derosa, L. Zitvogel, Cancer induces a stress ileopathy depending on β-adrenergic receptors and promoting dysbiosis that contributes to carcinogenesis. Cancer Discov. 12, 1128–1151 (2022). 34930787 10.1158/2159-8290.CD-21-0999

[75] H. Chen, X. Cong, C. Wu, X. Wu, J. Wang, K. Mao, J. Li, G. Zhu, F. Liu, X. Meng, J. Song, X. Sun, X. Wang, S. Liu, S. Zhang, X. Yang, Y. Song, Y. G. Yang, T. Sun, Intratumoral delivery of CCL25 enhances immunotherapy against triple-negative breast cancer by recruiting CCR9^+^ T cells. Sci. Adv. 6, eaax4690 (2020). 32064335 10.1126/sciadv.aax4690 PMC6989134

[76] K. A. Papadakis, C. Landers, J. Prehn, E. A. Kouroumalis, S. T. Moreno, J. C. Gutierrez-Ramos, M. R. Hodge, S. R. Targan, CC chemokine receptor 9 expression defines a subset of peripheral blood lymphocytes with mucosal T cell phenotype and Th1 or T-regulatory 1 cytokine profile. J. Immunol. 171, 159–165 (2003). 12816994 10.4049/jimmunol.171.1.159

[77] T. Stuart, A. Butler, P. Hoffman, C. Hafemeister, E. Papalexi, W. M. Mauck III, Y. Hao, M. Stoeckius, P. Smibert, R. Satija, Comprehensive integration of single-cell data. Cell 177, 1888–1902.e21 (2019). 31178118 10.1016/j.cell.2019.05.031 PMC6687398

[78] F. Grandi, J. Caroli, O. Romano, M. Marchionni, M. Forcato, S. Bicciato, popsicleR: A R package for pre-processing and quality control analysis of single cell RNA-seq data. J. Mol. Biol. 434, 167560 (2022). 35662457 10.1016/j.jmb.2022.167560

[79] S. L. Wolock, R. Lopez, A. M. Klein, Scrublet: Computational identification of cell doublets in single-cell transcriptomic data. Cell Syst. 8, 281–291.e9 (2019). 30954476 10.1016/j.cels.2018.11.005 PMC6625319

[80] E. Notario, G. Visci, B. Fosso, C. Gissi, N. Tanaskovic, M. Rescigno, M. Marzano, G. Pesole, Amplicon-based microbiome profiling: From second- to third-generation sequencing for higher taxonomic resolution. Genes 14, 1567 (2023). 37628619 10.3390/genes14081567 PMC10454624

[81] B. J. Callahan, J. Wong, C. Heiner, S. Oh, C. M. Theriot, A. S. Gulati, S. K. McGill, M. K. Dougherty, High-throughput amplicon sequencing of the full-length 16S rRNA gene with single-nucleotide resolution. Nucleic Acids Res. 47, e103 (2019). 31269198 10.1093/nar/gkz569 PMC6765137

[82] M. A. Nadkarni, F. E. Martin, N. A. Jacques, N. Hunter, Determination of bacterial load by real-time PCR using a broad-range (universal) probe and primers set. Microbiology 148, 257–266 (2002). 11782518 10.1099/00221287-148-1-257

[83] A. Romagnani, V. Vettore, T. Rezzonico-Jost, S. Hampe, E. Rottoli, W. Nadolni, M. Perotti, M. A. Meier, C. Hermanns, S. Geiger, G. Wennemuth, C. Recordati, M. Matsushita, S. Muehlich, M. Proietti, V. Chubanov, T. Gudermann, F. Grassi, S. Zierler, TRPM7 kinase activity is essential for T cell colonization and alloreactivity in the gut. Nat. Commun. 8, 1917 (2017). 29203869 10.1038/s41467-017-01960-z PMC5714948

[84] P.-L. Chen, W. Roh, A. Reuben, Z. A. Cooper, C. N. Spencer, P. A. Prieto, J. P. Miller, R. L. Bassett, V. Gopalakrishnan, K. Wani, M. P. De Macedo, J. L. Austin-Breneman, H. Jiang, Q. Chang, S. M. Reddy, W.-S. Chen, M. T. Tetzlaff, R. J. Broaddus, M. A. Davies, J. E. Gershenwald, L. Haydu, A. J. Lazar, S. P. Patel, P. Hwu, W.-J. Hwu, A. Diab, I. C. Glitza, S. E. Woodman, L. M. Vence, I. I. Wistuba, R. N. Amaria, L. N. Kwong, V. Prieto, R. E. Davis, W. Ma, W. W. Overwijk, A. H. Sharpe, J. Hu, P. A. Futreal, J. Blando, P. Sharma, J. P. Allison, L. Chin, J. A. Wargo, Analysis of immune signatures in longitudinal tumor samples yields insight into biomarkers of response and mechanisms of resistance to immune checkpoint blockade. Cancer Discov. 6, 827–837 (2016). 27301722 10.1158/2159-8290.CD-15-1545 PMC5082984

[85] N. Riaz, J. J. Havel, V. Makarov, A. Desrichard, W. J. Urba, J. S. Sims, F. S. Hodi, S. Martin-Algarra, R. Mandal, W. H. Sharfman, S. Bhatia, W. J. Hwu, T. F. Gajewski, C. L. Slingluff Jr., D. Chowell, S. M. Kendall, H. Chang, R. Shah, F. Kuo, L. G. T. Morris, J. W. Sidhom, J. P. Schneck, C. E. Horak, N. Weinhold, T. A. Chan, Tumor and microenvironment evolution during immunotherapy with nivolumab. Cell 171, 934–949.e16 (2017). 29033130 10.1016/j.cell.2017.09.028 PMC5685550

[86] M. D. Robinson, D. J. McCarthy, G. K. Smyth, edgeR: A bioconductor package for differential expression analysis of digital gene expression data. Bioinformatics 26, 139–140 (2010). 19910308 10.1093/bioinformatics/btp616 PMC2796818

[87] X. Cheng, Y. Liu, J. Wang, Y. Chen, A. G. Robertson, X. Zhang, S. J. M. Jones, S. Taubert, cSurvival: A web resource for biomarker interactions in cancer outcomes and in cell lines. Brief. Bioinform. 23, bbac090 (2022). 35368077 10.1093/bib/bbac090 PMC9116376

[88] F. A. Wolf, P. Angerer, F. J. Theis, SCANPY: Large-scale single-cell gene expression data analysis. Genome Biol. 19, 15 (2018). 29409532 10.1186/s13059-017-1382-0 PMC5802054

