# Supplementary material for: Secretory IgA amplification during immune checkpoint blockade enhances the control of tumor growth by enterotropic T cells
Source: Sci Adv. 2025 Oct 3;11(40):eaeb5308. doi: 10.1126/sciadv.aeb5308 (PMC12494019; doi:10.1126/sciadv.aeb5308)
Supplement: Supplementary file 1 — Figs. S1 to S13 Legends for tables S1 to S4 [file sciadv.aeb5308_sm.pdf]

Science Advances
AAAS

Supplementary Materials for

Secretory IgA amplification during immune checkpoint blockade enhances the control of tumor growth by enterotropic T cells

Benedetta De Ponte Conti *et al.*

Corresponding author: Fabio Grassi, fabio.grassi@irb.usi.ch

Sci. Adv. **11**, eaeb5308 (2025)
DOI: 10.1126/sciadv.aeb5308

The PDF file includes:

Figs. S1 to S13
Legends for tables S1 to S4

Other Supplementary Material for this manuscript includes the following:

Tables S1 to S4

SUPPLEMENTARY FIGURES AND FIGURE LEGENDS

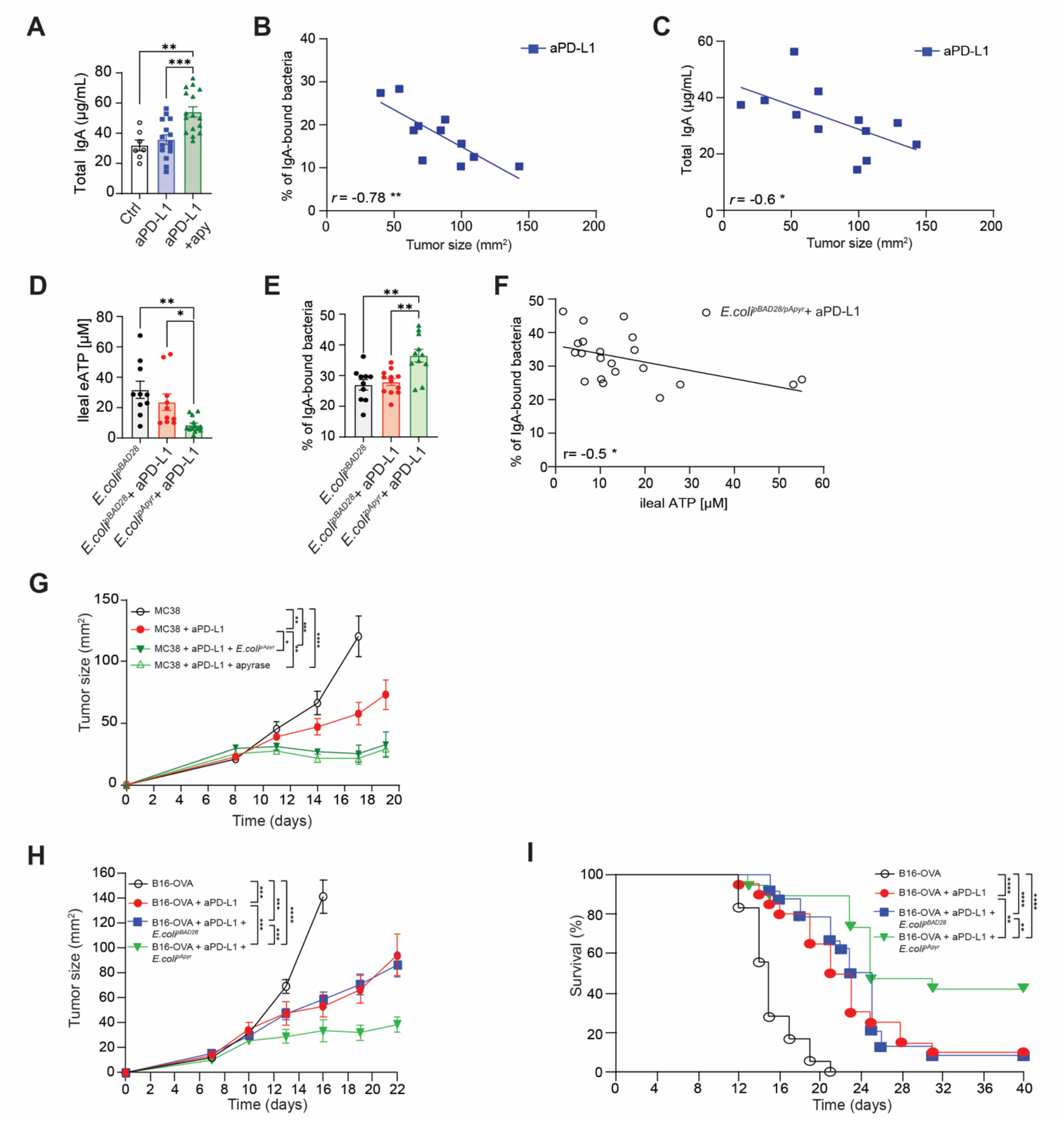

Fig. S1. SIgA correlates with response to immunotherapy and apyrase treatment is effective in boosting anti-tumor immunity. (**A**) ELISA of total IgA in the ileum of mice treated as in Figure 1A. Data are presented as the mean ± SEM from two experiments pooled together. (**B**) Correlation between IgA-coated bacteria or (**C**) total IgA in the ileum and tumor size at day 18 in anti-PD-L1 treated mice. Data points represent single mice. Data from two or three pooled experiments are shown, respectively. Pearson correlation coefficient (r) and p-value are shown. (**D**-**F**) Mice were daily gavaged with either 10^{10} CFUs of *E. coli*^{pBAD28} or *E. coli*^{pApyr} for 7 days. On day 3 and 6 mice were intraperitoneally treated with 100 µg of isotype control or anti-PD-L1 and mice were euthanized on day 8. Bar plot of ATP concentration (**D**) and IgA-coated bacteria (**E**) in the lumen of ileum from differently treated mice. (**F**) Correlation between ileal ATP concentration and % of IgA-coated bacteria in all mice treated with anti-PD-L1. Pearson correlation coefficient (r) and p-value are shown. Data points represent single mice. Data from 2 pooled experiments. Error bars represent SEM. One-way ANOVA with Turkey's multiple comparison test and Pearson correlation were used. *p<0.05, **p<0.01. (**G**) Tumor growth of mice treated with isotype or anti PD-L1 alone or in combination with 40µg pure apyrase or PBS or *E.coli*$^{pA\mathrm{pyr}}$ (n=6-20 mice/group). Data are presented as the mean ± SEM from 2 experiments pooled together. (**H-I**) Mice were injected with $1x10^6$ B16-OVA tumor cells and starting from day 5, when tumors were measurable, mice were daily gavaged with either 10^{10} CFUs of *E coli*^{pBAD28} or *E.coli*$^{pA\mathrm{pyr}}$. On days 8, 11, 14 and 17 after tumor inoculation, mice were intraperitoneally treated with 100 µg of either isotype control or anti-PD-L1 antibodies. (**H**) Tumor size and (**I**) survival (n=17-20 mice/group). Two-way ANOVA for tumor growth and Mantel-Cox log-rank test for survival curve were performed. *p < 0.05, **p < 0.01, ***p < 0.001.

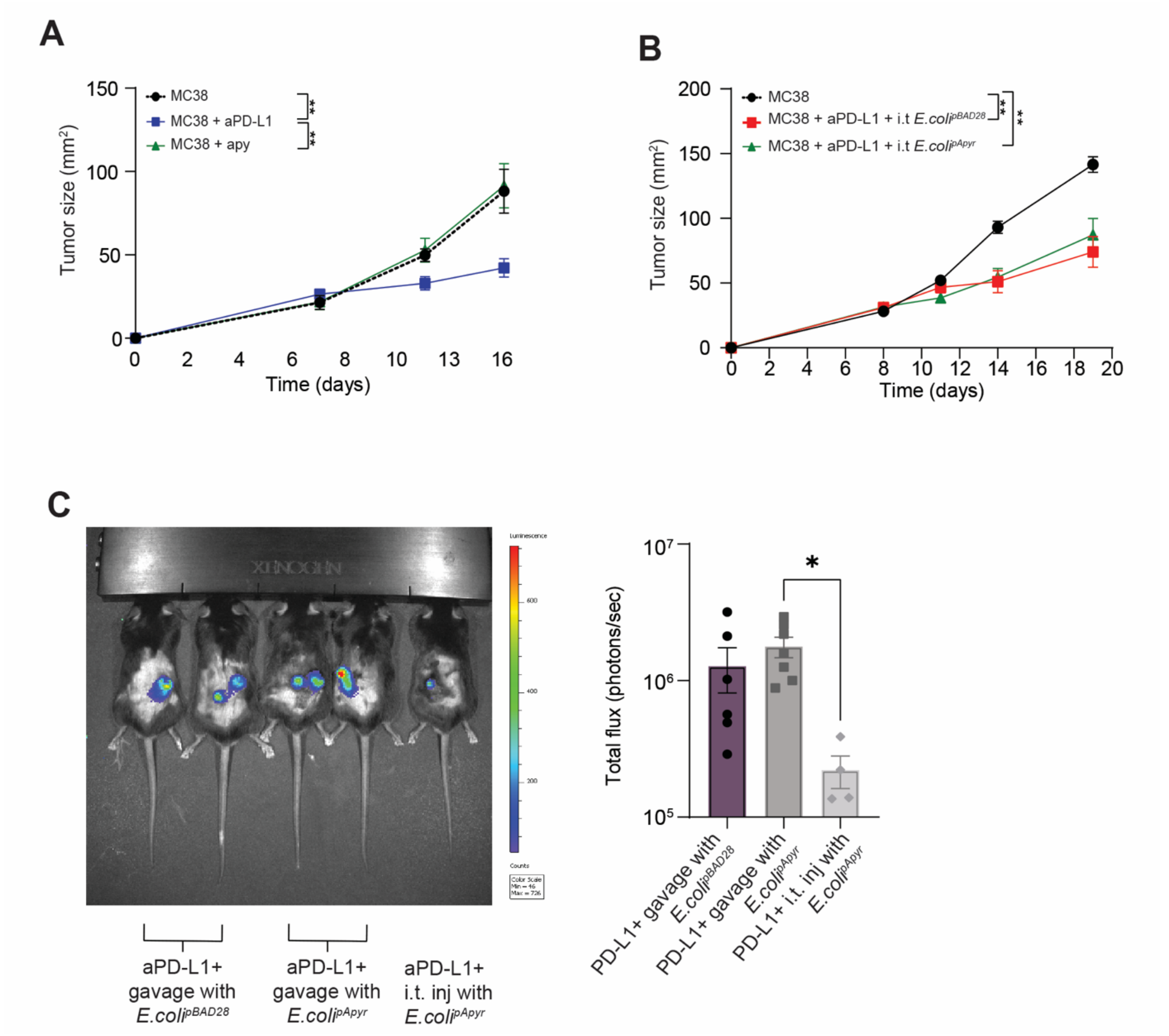

Fig. S2. Apyrase mediated anti-tumor immunity is not effective in absence of ICB and apyrase does not influence ICB within the TME. (**A**) Tumor size of MC38 tumor bearing mice receiving either anti-PD-L1 i.p. or apyrase via oral gavage (n=10-18 mice/group). Data are presented as the mean ± SEM from two experiments pooled together. (**B**) Tumor size of MC38 tumor bearing mice receiving anti-PD-L1 and intratumor injection of *E coli*^{pBAD28} or *E.coli*$^{pA\mathrm{pyr}}$ (n=15 mice/group). Data are presented as the mean ± SEM from two experiments pooled together. (**C**) Imaging of C57BL/6 mice with IVIS luminometer at day 12 after subcutaneous engraftment of melanoma B16-pmeLUC cells (tumour size ≅ 50 mm^2). From day 5 mice received oral gavage of *E coli*^{pBAD28} or *E.coli*$^{pA\mathrm{pyr}}$ or intratumor injection of *E.coli*$^{pA\mathrm{pyr}}$ on day 5, 7, 9 and 11. All three groups received one injection of anti-PD-L1 on day 11. Bioluminescence was recorded after i.p. injection of D/luciferin; values (photons/sec) among groups are reported on the right (n=4-6 mice/group). $^*p < 0.05$, $^{**}p < 0.01$.

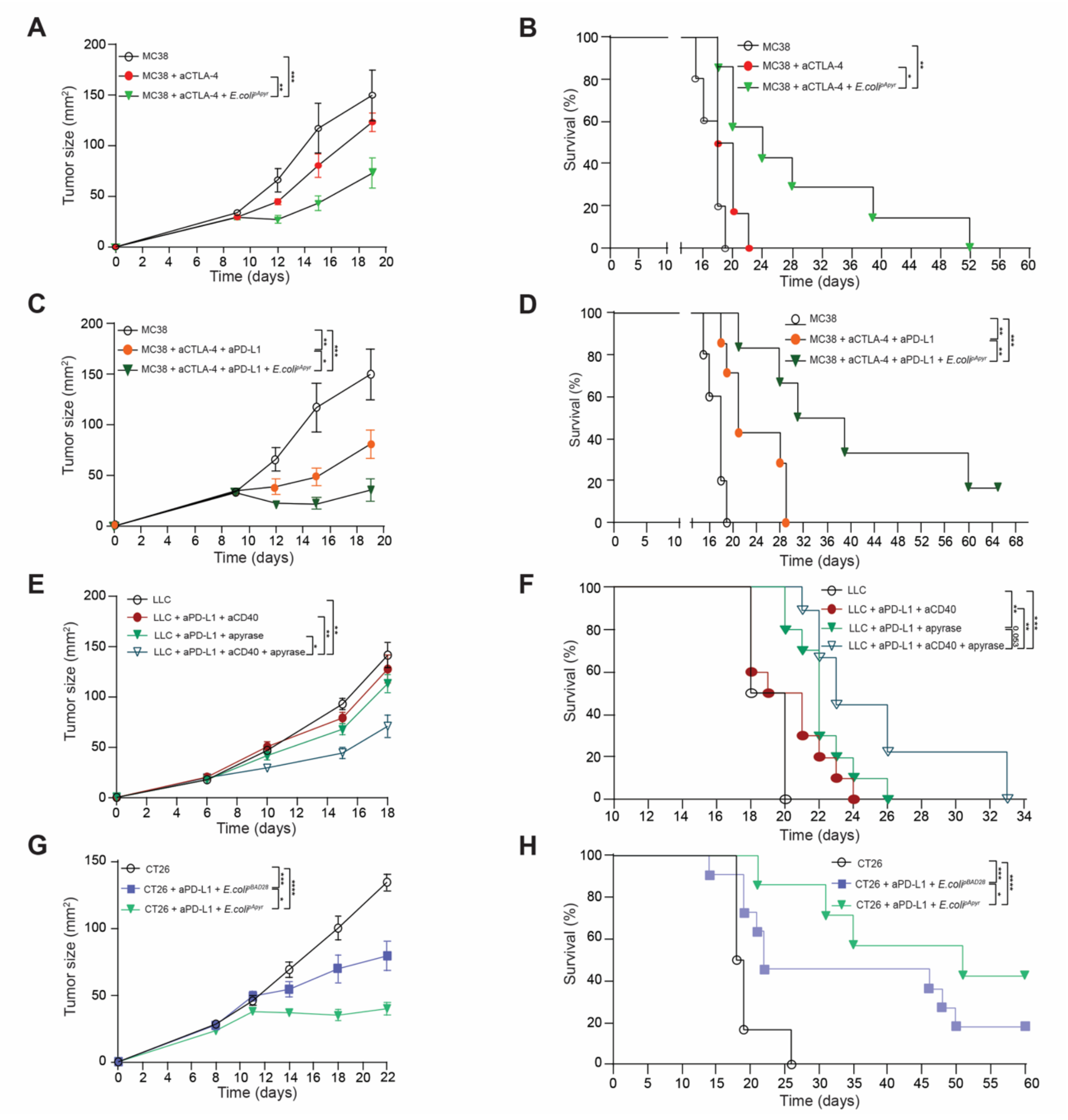

Fig. S3. Apyrase is effective in multiple tumor models and different ICB protocols. (**A-D**) Tumor growth (**A,C**) and survival (**B,D**) curves of mice treated as in Figure 1A either with isotype, anti-CTLA-4 or anti PD-L1/anti CTLA-4 either as standalone ICB or in combination with *E.coli*^{pA}pyr (n=7 mice/group). Data are presented as the mean ± SEM. (**E-F**) Tumor size (**E**) and survival (**F**) of C57BL/6 subcutaneously injected with 6 x 10^5 of LLC cells and on days 8, 11, 14 and 17 treated with anti-PD-L1with or without anti-CD40 either combined or not with 40 µg of apyrase. Data are presented as the mean ± SEM from two experiments pooled together (n=6-9 mice/group). (**G-H**) Tumor size (**G**) and survival (**H**) of Balb/c mice subcutaneously injected with 1x10^6 CT26 tumor cells and treated as in Fig. 1A with isotype or anti-PD-L1 alone or in combination with *E.coli*^{pA}pyr. Data are presented as the mean ± SEM from two experiments pooled together (n=6-11 mice/group). Two-way ANOVA for tumor growth and Mantel-Cox log-rank test for survival curve were performed. *p < 0.05, **p < 0.01, ***p < 0.001.

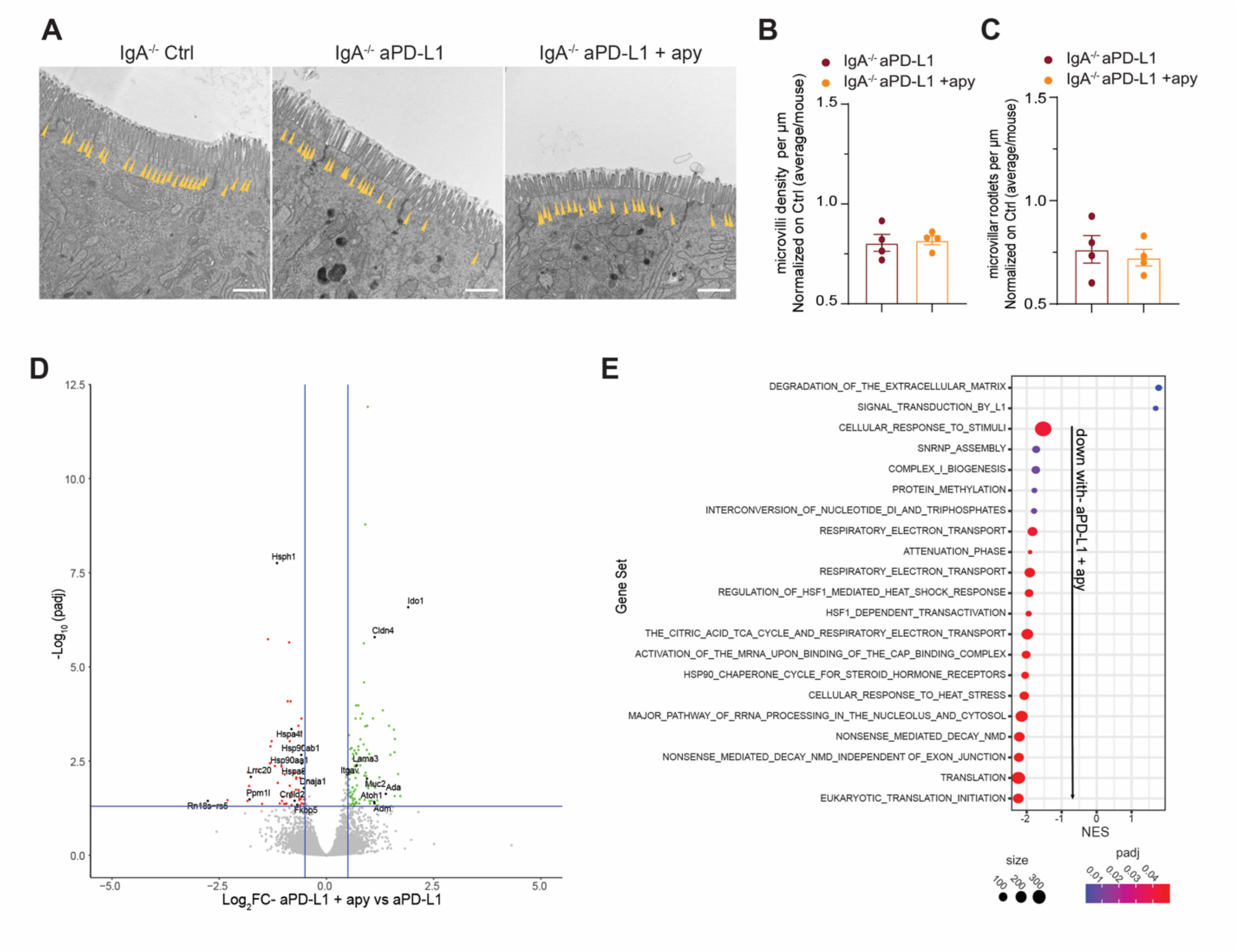

Fig. S4. Transcriptional analysis of IEC. (**A**)TEM images of enterocytes captured from the upper portion of villi from MC38 tumor bearing mice that were untreated (Ctrl, n=4), treated with standalone anti-PD-L1 or combined with apyrase in IgA$^{-/-}$ mice. Yellow arrowheads designate microvilli rootlets. TEM images are representative of 4 mice per group. Scale bar: 1 µm. (**B**,**C**) Graphs of average microvilli (**B**) and rootlet (**C**) densities quantified from TEM images are displayed as mean ± SEM. Data points represent single mice. (**D**) Volcano plot showing differential gene expression in epithelial cells from mice treated with standalone anti-PD-L1 (n=5) or combined with apyrase (n=4). For each gene, the differential expression (log2FoldChange) and the respective statistical significance (log10FDR-adjusted p-value) [log10(padj)] are shown. The green dots represent the genes with log2FoldChange >0.5 and padj <0.05. The red dots represent the genes with log2FoldChange <0.5 and padj <0.05. The analysis revealed 101 UP-regulated genes and 57 DOWN-regulated genes. The dots highlighted and labeled in black are genes of interest. (**E**) Plot illustrating the results of GSEA analysis. Pathways from Reactome database were used. The figure shows the top 2 positively (NES >0) and 19 negatively (NES <0) enriched pathways associated with anti-PD-L1 treatment in combination with apyrase with respect to mice treated with standalone anti-PD-L1. The degree of significance is given by the adjusted p-value (padj) and represented in a scale from blue to red. The dot size indicates the number of genes associated to a specific pathway.

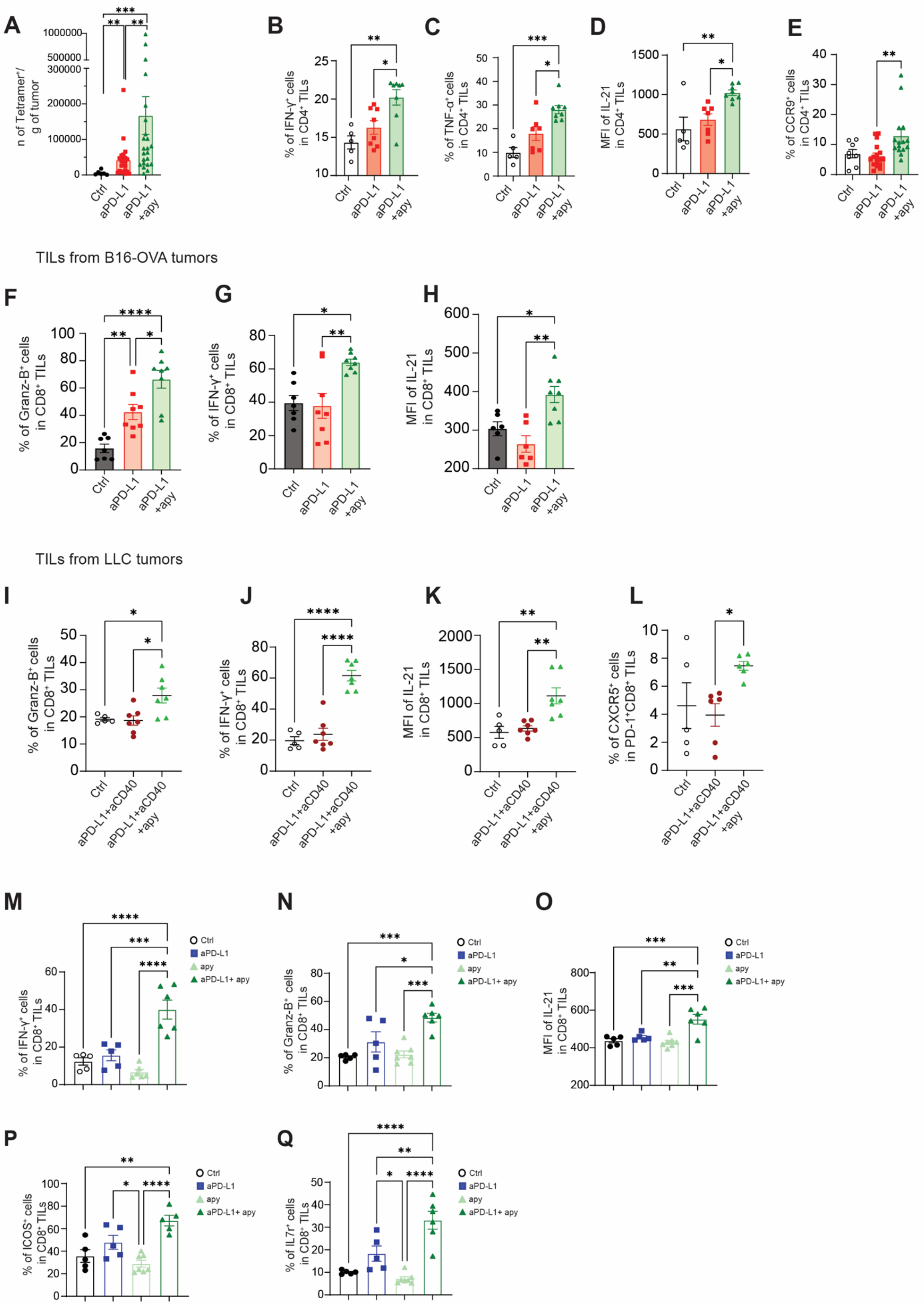

A
n of Tetramer+/ g of tumor
Ctrl
aPD-L1
aPD-L1 +apy
B
% of IFN-γ+ cells in CD4+ TILs
C
% of TNF-α+ cells in CD4+ TILs
D
MFI of IL-21 in CD4+ TILs
E
% of CCR9+ cells in CD4+ TILs
TILs from B16-OVA tumors
F
% of Granz-B+ cells in CD8+ TILs
G
% of IFN-γ+ cells in CD8+ TILs
H
MFI of IL-21 in CD8+ TILs
TILs from LLC tumors
I
% of Granz-B+ cells in CD8+ TILs
aPD-L1+aCD40
aPD-L1+aCD40 +apy
J
% of IFN-γ+ cells in CD8+ TILs
K
MFI of IL-21 in CD8+ TILs
L
% of CXCR5+ cells in PD-1+CD8+ TILs
M
% of IFN-γ+ cells in CD8+ TILs
Ctrl
aPD-L1
apy
aPD-L1+ apy
N
% of Granz-B+ cells in CD8+ TILs
O
MFI of IL-21 in CD8+ TILs
P
% of ICOS+ cells in CD8+ TILs
Q
% of IL7r+ cells in CD8+ TILs

Fig S5. Increased tumor specific CD8 cells by apyrase combination with ICB, differential phenotype of CD4 TILs and increased CD8 effector functions in TILs from different tumors by apyrase within ICB but not as monotherapy. (**A**) Quantification of OVA-specific $CD8^+$ TILs in B16-OVA tumors recovered at day 12 of the schedule shown in Figure 1A. Cells were selected for $Zombie^-$, $CD45^+$, $CD8^+TCR\beta^+$, $Tetramer^+$ and analyzed on the gated lymphocyte population. Data points represent single mice. Data were from two experiments pooled together. Error bars represent SEM; Two-tailed Mann Whitney U test was used. (**B-E**) $CD4^+$ TILs subpopulations were recovered from MC38 tumors of day 12 (**B-D**) or 18 (**E**) of the schedule presented in Figure 1A. Histograms and bar graphs reflect the proportion of IFN-γ^+ (**B**), TNF-α^+ (**C**), CCR9 (**E**) or mean fluorescence intensity (MFI) of IL21 (**D**) of $CD4^+$ TILs. Data points represent single mice. Error bars represent SEM. One-way ANOVA with Turkey's multiple comparison test was used. (**F-L**) Increased effector functions in TILs by apyrase administration in different tumor models. Bar plots of $CD8^+$ TILs subpopulations recovered from tumors at day 12 of the schedule shown in Fig. 1A (24 h after the 2nd dose of anti-PD-L1) of mice injected either with B16-OVA melanoma or Lewis lung carcinoma (LLC). Cells were selected as $Zombie^-$, $CD45^+$, $CD8^+TCR\beta^+$ and analyzed on the gated lymphocyte population. Bar graphs reflect the proportion of Granzyme B^+ (**F,I**), IFN-γ^+ (**G**,**J**), $CXCR5^+$ (**L**) and mean fluorescence intensity (MFI) of IL-21 (**H**,**K**) in the indicated subsets of $CD8^+$ T cells. The boundaries to quantify positive cells with the different staining were established using isotype matched negative antibodies. Data points represent single mice. Error bars represent SEM. One-way ANOVA with Turkey's multiple comparison test was used. (**M-Q**) Bar graphs showing the proportion of IFN-γ^+ (**M**), Granzyme B^+ (**N**), mean fluorescence intensity (MFI) of IL21 (**O**) or the proportion of $ICOS^+$ (**P**) and $IL7r^+$ (**Q**) $CD8^+$ TILs from mice treated with PBS, standalone anti-PD-L1 or apyrase alone or in combination with anti-PD-L1. Data points represent single mice. Error bars represent SEM. One-way ANOVA with Turkey's multiple comparison test was used. $^*p < 0.05$, $^{**}p < 0.01$, $^{***}p < 0.001$, $^{****}p < 0.0001$.

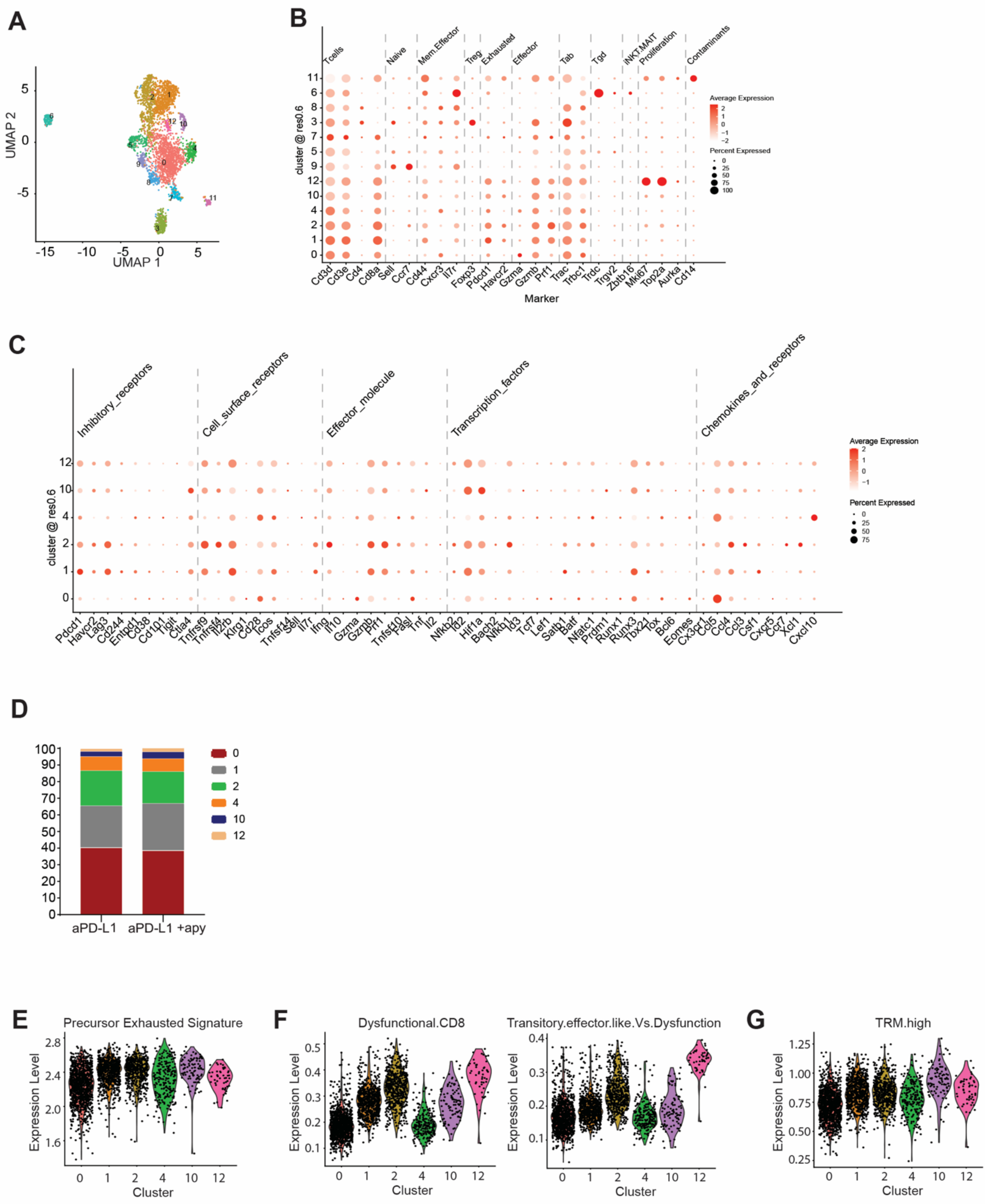

A
B
C
D
E
F
G
UMAP 1
UMAP 2
Marker
cluster @ res0.6
Average Expression
Percent Expressed
Tcells
Naive
Mem.Effector
Treg
Exhausted
Effector
Tab
Tgd
iNKT.MAIT
Proliferation
Contaminants
Inhibitory_receptors
Cell_surface_receptors
Effector_molecule
Transcription_factors
Chemokines_and_receptors
aPD-L1
aPD-L1 +apy
Precursor Exhausted Signature
Dysfunctional.CD8
Transitory.effector.like.Vs.Dysfunction
TRM.high
Expression Level
Cluster

Fig. S6. CD8 clustering and transcriptional regulation in CD8 TILs by apyrase combination to ICB. (**A**) Uniform manifold approximation and projection (UMAP) of the single-cell RNA-seq dataset comprising T cells infiltrating tumors treated with anti-PD-L1 or anti-PD-L1 plus apyrase. Cells are colored according to cluster identity. (**B**) Dot plot showing the expression of markers used to annotate clusters of T cells. Dot size is proportional to the percentage of cluster cells expressing the gene, dot color represents standardized average expression. (**C**) Dot plot showing the expression of markers related to T cell biology in clusters of CD8 T cells. Dot size is proportional to the percentage of cluster cells expressing the gene, dot color represents standardized average expression. **(D)** CD8 clusters composition amongst anti-PD-L1 and anti-PD-L1 plus apyrase treated groups was compared using Fisher's Exact Test. (**E-G**) Violin plots showing the expression of gene signatures representing diverse CD8 T cell states in CD8 T cells clusters.

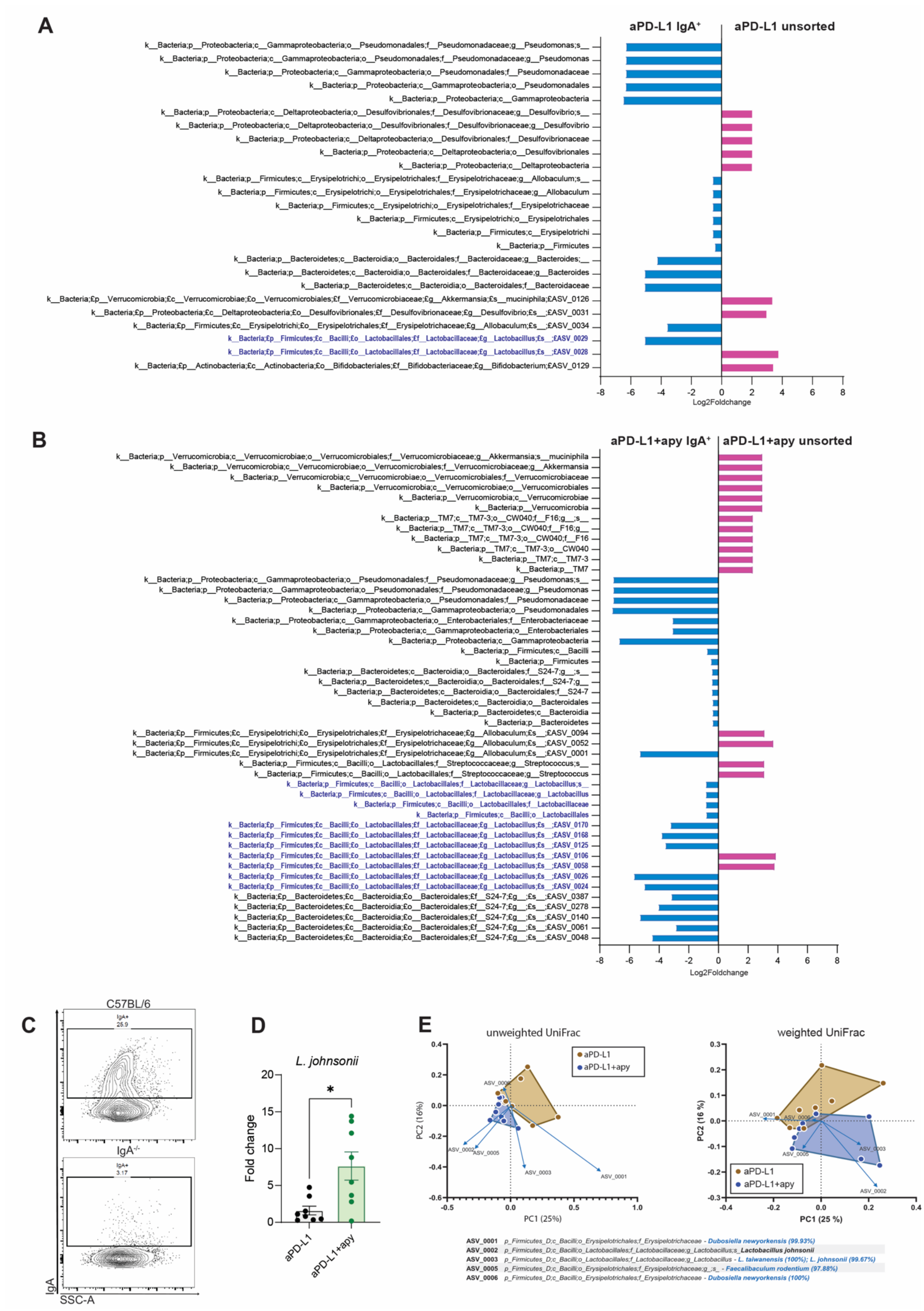

A
aPD-L1 IgA⁺
aPD-L1 unsorted
k__Bacteria;p__Proteobacteria;c__Gammaproteobacteria;o__Pseudomonadales;f__Pseudomonadaceae;g__Pseudomonas;s__
k__Bacteria;p__Proteobacteria;c__Gammaproteobacteria;o__Pseudomonadales;f__Pseudomonadaceae;g__Pseudomonas
k__Bacteria;p__Proteobacteria;c__Gammaproteobacteria;o__Pseudomonadales;f__Pseudomonadaceae
k__Bacteria;p__Proteobacteria;c__Gammaproteobacteria;o__Pseudomonadales
k__Bacteria;p__Proteobacteria;c__Gammaproteobacteria
k__Bacteria;p__Proteobacteria;c__Deltaproteobacteria;o__Desulfovibrionales;f__Desulfovibrionaceae;g__Desulfovibrio;s__
k__Bacteria;p__Proteobacteria;c__Deltaproteobacteria;o__Desulfovibrionales;f__Desulfovibrionaceae;g__Desulfovibrio
k__Bacteria;p__Proteobacteria;c__Deltaproteobacteria;o__Desulfovibrionales;f__Desulfovibrionaceae
k__Bacteria;p__Proteobacteria;c__Deltaproteobacteria;o__Desulfovibrionales
k__Bacteria;p__Proteobacteria;c__Deltaproteobacteria
k__Bacteria;p__Firmicutes;c__Erysipelotrichi;o__Erysipelotrichales;f__Erysipelotrichaceae;g__Allobaculum;s__
k__Bacteria;p__Firmicutes;c__Erysipelotrichi;o__Erysipelotrichales;f__Erysipelotrichaceae;g__Allobaculum
k__Bacteria;p__Firmicutes;c__Erysipelotrichi;o__Erysipelotrichales;f__Erysipelotrichaceae
k__Bacteria;p__Firmicutes;c__Erysipelotrichi;o__Erysipelotrichales
k__Bacteria;p__Firmicutes;c__Erysipelotrichi
k__Bacteria;p__Firmicutes
k__Bacteria;p__Bacteroidetes;c__Bacteroidia;o__Bacteroidales;f__Bacteroidaceae;g__Bacteroides;__
k__Bacteria;p__Bacteroidetes;c__Bacteroidia;o__Bacteroidales;f__Bacteroidaceae;g__Bacteroides
k__Bacteria;p__Bacteroidetes;c__Bacteroidia;o__Bacteroidales;f__Bacteroidaceae
k__Bacteria;£p__Verrucomicrobia;£c__Verrucomicrobiae;£o__Verrucomicrobiales;£f__Verrucomicrobiaceae;£g__Akkermansia;£s__muciniphila;£ASV_0126
k__Bacteria;£p__Proteobacteria;£c__Deltaproteobacteria;£o__Desulfovibrionales;£f__Desulfovibrionaceae;£g__Desulfovibrio;£s__;£ASV_0031
k__Bacteria;£p__Firmicutes;£c__Erysipelotrichi;£o__Erysipelotrichales;£f__Erysipelotrichaceae;£g__Allobaculum;£s__;£ASV_0034
k__Bacteria;£p__Firmicutes;£c__Bacilli;£o__Lactobacillales;£f__Lactobacillaceae;£g__Lactobacillus;£s__;£ASV_0029
k__Bacteria;£p__Firmicutes;£c__Bacilli;£o__Lactobacillales;£f__Lactobacillaceae;£g__Lactobacillus;£s__;£ASV_0028
k__Bacteria;£p__Actinobacteria;£c__Actinobacteria;£o__Bifidobacteriales;£f__Bifidobacteriaceae;£g__Bifidobacterium;£ASV_0129
-8 -6 -4 -2 0 2 4 6 8
Log2Foldchange
B
aPD-L1+apy IgA⁺
aPD-L1+apy unsorted
k__Bacteria;p__Verrucomicrobia;c__Verrucomicrobiae;o__Verrucomicrobiales;f__Verrucomicrobiaceae;g__Akkermansia;s__muciniphila
k__Bacteria;p__Verrucomicrobia;c__Verrucomicrobiae;o__Verrucomicrobiales;f__Verrucomicrobiaceae;g__Akkermansia
k__Bacteria;p__Verrucomicrobia;c__Verrucomicrobiae;o__Verrucomicrobiales;f__Verrucomicrobiaceae
k__Bacteria;p__Verrucomicrobia;c__Verrucomicrobiae;o__Verrucomicrobiales
k__Bacteria;p__Verrucomicrobia;c__Verrucomicrobiae
k__Bacteria;p__Verrucomicrobia
k__Bacteria;p__TM7;c__TM7-3;o__CW040;f__F16;g__;s__
k__Bacteria;p__TM7;c__TM7-3;o__CW040;f__F16;g__
k__Bacteria;p__TM7;c__TM7-3;o__CW040;f__F16
k__Bacteria;p__TM7;c__TM7-3;o__CW040
k__Bacteria;p__TM7;c__TM7-3
k__Bacteria;p__TM7
k__Bacteria;p__Proteobacteria;c__Gammaproteobacteria;o__Pseudomonadales;f__Pseudomonadaceae;g__Pseudomonas;s__
k__Bacteria;p__Proteobacteria;c__Gammaproteobacteria;o__Pseudomonadales;f__Pseudomonadaceae;g__Pseudomonas
k__Bacteria;p__Proteobacteria;c__Gammaproteobacteria;o__Pseudomonadales;f__Pseudomonadaceae
k__Bacteria;p__Proteobacteria;c__Gammaproteobacteria;o__Pseudomonadales
k__Bacteria;p__Proteobacteria;c__Gammaproteobacteria;o__Enterobacteriales;f__Enterobacteriaceae
k__Bacteria;p__Proteobacteria;c__Gammaproteobacteria;o__Enterobacteriales
k__Bacteria;p__Proteobacteria;c__Gammaproteobacteria
k__Bacteria;p__Firmicutes;c__Bacilli
k__Bacteria;p__Firmicutes
k__Bacteria;p__Bacteroidetes;c__Bacteroidia;o__Bacteroidales;f__S24-7;g__;s__
k__Bacteria;p__Bacteroidetes;c__Bacteroidia;o__Bacteroidales;f__S24-7;g__
k__Bacteria;p__Bacteroidetes;c__Bacteroidia;o__Bacteroidales;f__S24-7
k__Bacteria;p__Bacteroidetes;c__Bacteroidia;o__Bacteroidales
k__Bacteria;p__Bacteroidetes;c__Bacteroidia
k__Bacteria;p__Bacteroidetes
k__Bacteria;£p__Firmicutes;£c__Erysipelotrichi;£o__Erysipelotrichales;£f__Erysipelotrichaceae;£g__Allobaculum;£s__;£ASV_0094
k__Bacteria;£p__Firmicutes;£c__Erysipelotrichi;£o__Erysipelotrichales;£f__Erysipelotrichaceae;£g__Allobaculum;£s__;£ASV_0052
k__Bacteria;£p__Firmicutes;£c__Erysipelotrichi;£o__Erysipelotrichales;£f__Erysipelotrichaceae;£g__Allobaculum;£s__;£ASV_0001
k__Bacteria;p__Firmicutes;c__Bacilli;o__Lactobacillales;f__Streptococcaceae;g__Streptococcus;s__
k__Bacteria;p__Firmicutes;c__Bacilli;o__Lactobacillales;f__Streptococcaceae;g__Streptococcus
k__Bacteria;p__Firmicutes;c__Bacilli;o__Lactobacillales;f__Lactobacillaceae;g__Lactobacillus;s__
k__Bacteria;p__Firmicutes;c__Bacilli;o__Lactobacillales;f__Lactobacillaceae;g__Lactobacillus
k__Bacteria;p__Firmicutes;c__Bacilli;o__Lactobacillales;f__Lactobacillaceae
k__Bacteria;p__Firmicutes;c__Bacilli;o__Lactobacillales
k__Bacteria;£p__Firmicutes;£c__Bacilli;£o__Lactobacillales;£f__Lactobacillaceae;£g__Lactobacillus;£s__;£ASV_0170
k__Bacteria;£p__Firmicutes;£c__Bacilli;£o__Lactobacillales;£f__Lactobacillaceae;£g__Lactobacillus;£s__;£ASV_0168
k__Bacteria;£p__Firmicutes;£c__Bacilli;£o__Lactobacillales;£f__Lactobacillaceae;£g__Lactobacillus;£s__;£ASV_0125
k__Bacteria;£p__Firmicutes;£c__Bacilli;£o__Lactobacillales;£f__Lactobacillaceae;£g__Lactobacillus;£s__;£ASV_0106
k__Bacteria;£p__Firmicutes;£c__Bacilli;£o__Lactobacillales;£f__Lactobacillaceae;£g__Lactobacillus;£s__;£ASV_0058
k__Bacteria;£p__Firmicutes;£c__Bacilli;£o__Lactobacillales;£f__Lactobacillaceae;£g__Lactobacillus;£s__;£ASV_0026
k__Bacteria;£p__Firmicutes;£c__Bacilli;£o__Lactobacillales;£f__Lactobacillaceae;£g__Lactobacillus;£s__;£ASV_0024
k__Bacteria;£p__Bacteroidetes;£c__Bacteroidia;£o__Bacteroidales;£f__S24-7;£g__;£s__;£ASV_0387
k__Bacteria;£p__Bacteroidetes;£c__Bacteroidia;£o__Bacteroidales;£f__S24-7;£g__;£s__;£ASV_0278
k__Bacteria;£p__Bacteroidetes;£c__Bacteroidia;£o__Bacteroidales;£f__S24-7;£g__;£s__;£ASV_0140
k__Bacteria;£p__Bacteroidetes;£c__Bacteroidia;£o__Bacteroidales;£f__S24-7;£g__;£s__;£ASV_0061
k__Bacteria;£p__Bacteroidetes;£c__Bacteroidia;£o__Bacteroidales;£f__S24-7;£g__;£s__;£ASV_0048
-8 -6 -4 -2 0 2 4 6 8
Log2Foldchange
C
C57BL/6
IgA+
25.9
IgA-/-
IgA+
3.17
IgA
SSC-A
D
L. johnsonii
*
Fold change
0 5 10 15 20
aPD-L1
aPD-L1+apy
E
unweighted UniFrac
aPD-L1
aPD-L1+apy
ASV_0006
ASV_0002
ASV_0005
ASV_0003
ASV_0001
PC2 (16%)
PC1 (25%)
weighted UniFrac
ASV_0001
ASV_0006
ASV_0005
ASV_0003
ASV_0002
PC2 (16 %)
PC1 (25 %)
ASV_0001 p_Firmicutes_D;c_Bacilli;o_Erysipelotrichales;f_Erysipelotrichaceae - Dubosiella newyorkensis (99.93%)
ASV_0002 p_Firmicutes_D;c_Bacilli;o_Lactobacillales;f_Lactobacillaceae;g_Lactobacillus;s_Lactobacillus johnsonii
ASV_0003 p_Firmicutes_D;c_Bacilli;o_Lactobacillales;f_Lactobacillaceae;g_Lactobacillus - L. taiwanensis (100%); L. johnsonii (99.67%)
ASV_0005 p_Firmicutes_D;c_Bacilli;o_Erysipelotrichales;f_Erysipelotrichaceae;g_;s_ - Faecalibaculum rodentium (97.88%)
ASV_0006 p_Firmicutes_D;c_Bacilli;o_Erysipelotrichales;f_Erysipelotrichaceae - Dubosiella newyorkensis (100%)

Fig. S7. Gating strategy for IgA-Seq, differential abundance analysis, qPCR of *L. johnsonii* and beta diversity of ileal microbiota. (**A-B**) Differential abundance analyses performed with DESeq2 comparing microbial taxa in the *IgA*$^+$ fraction versus unsorted bacteria from mice treated with standalone anti-*PD-L1* (**A**) and IgA$^+$ fraction versus unsorted bacteria from mice treated with anti-PD-L1 and apyrase (**B**). Each panel displays the $\log_2$ fold changes of bacterial taxa that are significantly different, as determined by Wald tests followed by Hochberg correction for multiple testing. Taxa belonging to the genus *Lactobacillus* are highlighted in blue. All taxa shown met the statistical significance threshold after correction (padj<0.05). (**C**) Representative dot plot of IgA-coated bacteria in the ileum of WT or IgA$^{-/-}$ C57BL/6 mice and gating strategy for sorting of the IgA$^+$ fraction. (**D**) Quantification of *L. johnsonii* by qPCR in the ileal content of mice treated with standalone anti-PD-L1 or combined with apyrase, 18 days post tumor injection. Data are expressed as fold change relative to EUB quantification. (**E**) Biplot of the beta-diversity analysis based on the weighted and unweighted UniFrac distances. ASV, amplicon sequence variant. PC, principal coordinate. The first five bacterial taxa that explain more the variance among samples are indicated. Taxonomic names written in blue were determined through a manual BLASTN search in GenBank using the sequence of the corresponding ASV (percentages of DNA sequence identity are reported among brackets).

Taxonomy	*padj*	aPD-L1 +apy	aPD-L1 +apy +Vanco
d__Bacteria;p__Actinobacteriota;c__Actinobacteria;o__Bifidobacteriales;f__Bifidobacteriaceae;g__Bifidobacterium;s__Bifidobacterium_pseudolongum;ASV_0295.3v2	0.014266	1.94132	0
d__Bacteria;p__Bacteroidota;c__Bacteroidia;o__Bacteroidales;f__Muribaculaceae;g__Muribaculaceae;s__uncultured_bacterium;ASV_0018.3v2	0.001953	2.474972	3.942032
d__Bacteria;p__Bacteroidota;c__Bacteroidia;o__Bacteroidales;f__Muribaculaceae;g__Muribaculaceae;s__uncultured_bacterium;ASV_0077.3v2	0.013672	0	2.446171
d__Bacteria;p__Bacteroidota;c__Bacteroidia;o__Bacteroidales;f__Muribaculaceae;g__Muribaculaceae;s__uncultured_bacterium;ASV_0079.3v2	0.005922	3.384281	0
d__Bacteria;p__Bacteroidota;c__Bacteroidia;o__Bacteroidales;f__Muribaculaceae;g__Muribaculaceae;s__uncultured_bacterium;ASV_0080.3v2	0.009152	2.658864	0
d__Bacteria;p__Bacteroidota;c__Bacteroidia;o__Bacteroidales;f__Muribaculaceae;g__Muribaculaceae;s__uncultured_bacterium;ASV_0083.3v2	0.036032	0	2.191794
d__Bacteria;p__Bacteroidota;c__Bacteroidia;o__Bacteroidales;f__Muribaculaceae;g__Muribaculaceae;s__uncultured_bacterium;ASV_0091.3v2	0.000977	0	2.323584
d__Bacteria;p__Bacteroidota;c__Bacteroidia;o__Bacteroidales;f__Muribaculaceae;g__Muribaculaceae;s__uncultured_bacterium;ASV_0094.3v2	0.036032	0	2.068651
d__Bacteria;p__Bacteroidota;c__Bacteroidia;o__Bacteroidales;f__Muribaculaceae;g__Muribaculaceae;s__uncultured_bacterium;ASV_0099.3v2	0.005922	0	2.108079
d__Bacteria;p__Bacteroidota;c__Bacteroidia;o__Bacteroidales;f__Muribaculaceae;g__Muribaculaceae;s__uncultured_bacterium;ASV_0104.3v2	0.022494	2.131453	0
d__Bacteria;p__Bacteroidota;c__Bacteroidia;o__Bacteroidales;f__Muribaculaceae;g__Muribaculaceae;s__uncultured_bacterium;ASV_0106.3v2	0.036032	0	1.876607
d__Bacteria;p__Bacteroidota;c__Bacteroidia;o__Bacteroidales;f__Muribaculaceae;g__Muribaculaceae;s__uncultured_bacterium;ASV_0109.3v2	0.009152	2.283785	0
d__Bacteria;p__Bacteroidota;c__Bacteroidia;o__Bacteroidales;f__Muribaculaceae;g__Muribaculaceae;s__uncultured_bacterium;ASV_0113.3v2	0.014266	2.368689	0
d__Bacteria;p__Bacteroidota;c__Bacteroidia;o__Bacteroidales;f__Muribaculaceae;g__Muribaculaceae;s__uncultured_bacterium;ASV_0118.3v2	0.000977	2.597927	0
d__Bacteria;p__Bacteroidota;c__Bacteroidia;o__Bacteroidales;f__Muribaculaceae;g__Muribaculaceae;s__uncultured_bacterium;ASV_0128.3v2	0.014266	0	2.390318
d__Bacteria;p__Bacteroidota;c__Bacteroidia;o__Bacteroidales;f__Muribaculaceae;g__Muribaculaceae;s__uncultured_bacterium;ASV_0165.3v2	0.009152	2.432614	0
d__Bacteria;p__Bacteroidota;c__Bacteroidia;o__Bacteroidales;f__Muribaculaceae;g__Muribaculaceae;s__uncultured_bacterium;ASV_0190.3v2	0.009152	2.658864	0
d__Bacteria;p__Bacteroidota;c__Bacteroidia;o__Bacteroidales;f__Muribaculaceae;g__Muribaculaceae;s__uncultured_bacterium;ASV_0191.3v2	0.036032	2.51312	0
d__Bacteria;p__Bacteroidota;c__Bacteroidia;o__Bacteroidales;f__Muribaculaceae;g__Muribaculaceae;s__uncultured_bacterium;ASV_0203.3v2	0.022494	2.168676	0
d__Bacteria;p__Bacteroidota;c__Bacteroidia;o__Bacteroidales;f__Muribaculaceae;g__Muribaculaceae;s__uncultured_bacterium;ASV_0206.3v2	0.014266	2.163573	0
d__Bacteria;p__Bacteroidota;c__Bacteroidia;o__Bacteroidales;f__Muribaculaceae;g__Muribaculaceae;s__uncultured_bacterium;ASV_0214.3v2	0.014266	2.108076	0
d__Bacteria;p__Bacteroidota;c__Bacteroidia;o__Bacteroidales;f__Muribaculaceae;g__Muribaculaceae;s__uncultured_bacterium;ASV_0217.3v2	0.036032	2.151246	0
d__Bacteria;p__Bacteroidota;c__Bacteroidia;o__Bacteroidales;f__Muribaculaceae;g__Muribaculaceae;s__uncultured_bacterium;ASV_0226.3v2	0.036032	1.34152	0
d__Bacteria;p__Bacteroidota;c__Bacteroidia;o__Bacteroidales;f__Muribaculaceae;g__Muribaculaceae;s__uncultured_bacterium;ASV_0234.3v2	0.022494	2.345962	0
d__Bacteria;p__Bacteroidota;c__Bacteroidia;o__Bacteroidales;f__Muribaculaceae;g__Muribaculaceae;s__uncultured_bacterium;ASV_0300.3v2	0.036032	1.257954	0
d__Bacteria;p__Bacteroidota;c__Bacteroidia;o__Bacteroidales;f__Muribaculaceae;g__Muribaculaceae;s__uncultured_bacterium;ASV_0310.3v2	0.022494	2.051511	0
d__Bacteria;p__Bacteroidota;c__Bacteroidia;o__Bacteroidales;f__Tannerellaceae;g__Parabacteroides;ASV_0173.3v2	0.009152	0	1.383079
d__Bacteria;p__Bacteroidota;c__Bacteroidia;o__Bacteroidales;f__Tannerellaceae;g__Parabacteroides;ASV_0175.3v2	0.009152	0	1.556556
d__Bacteria;p__Bacteroidota;c__Bacteroidia;o__Bacteroidales;f__Tannerellaceae;g__Parabacteroides;ASV_0432.3v2	0.036032	0	0.411852
d__Bacteria;p__Firmicutes;c__Bacilli;o__Erysipelotrichales;f__Erysipelotrichaceae;g__Dubosiella;s__uncultured_bacterium;ASV_0022.3v2	0.000977	3.552135	0
d__Bacteria;p__Firmicutes;c__Bacilli;o__Erysipelotrichales;f__Erysipelotrichaceae;g__Dubosiella;s__uncultured_bacterium;ASV_0089.3v2	0.022494	2.672686	0
d__Bacteria;p__Firmicutes;c__Bacilli;o__Erysipelotrichales;f__Erysipelotrichaceae;g__Dubosiella;s__uncultured_bacterium;ASV_0110.3v2	0.036032	2.194628	0
d__Bacteria;p__Firmicutes;c__Bacilli;o__Erysipelotrichales;f__Erysipelotrichaceae;g__Faecalibaculum;s__Faecalibaculum_rodentium;ASV_0244.3v2	0.022494	2.232535	0
d__Bacteria;p__Firmicutes;c__Bacilli;o__Erysipelotrichales;f__Erysipelotrichaceae;g__Faecalibaculum;s__Faecalibaculum_rodentium;ASV_0267.3v2	0.036032	1.897018	0
**d__Bacteria;p__Firmicutes;c__Bacilli;o__Lactobacillales;f__Lactobacillaceae;g__Lactobacillus;s__Lactobacillus_johnsonii;ASV_0010.3v2**	0.000977	4.833606	0
**d__Bacteria;p__Firmicutes;c__Bacilli;o__Lactobacillales;f__Lactobacillaceae;g__Lactobacillus;s__Lactobacillus_johnsonii;ASV_0013.3v2**	0.000977	4.936981	0
**d__Bacteria;p__Firmicutes;c__Bacilli;o__Lactobacillales;f__Lactobacillaceae;g__Lactobacillus;s__Lactobacillus_johnsonii;ASV_0029.3v2**	0.005922	4.090346	0
**d__Bacteria;p__Firmicutes;c__Bacilli;o__Lactobacillales;f__Lactobacillaceae;g__Lactobacillus;s__Lactobacillus_johnsonii;ASV_0055.3v2**	0.022494	3.24074	0
**d__Bacteria;p__Firmicutes;c__Bacilli;o__Lactobacillales;f__Lactobacillaceae;g__Lactobacillus;s__Lactobacillus_johnsonii;ASV_0060.3v2**	0.005922	3.473616	0
**d__Bacteria;p__Firmicutes;c__Bacilli;o__Lactobacillales;f__Lactobacillaceae;g__Lactobacillus;s__Lactobacillus_johnsonii;ASV_0062.3v2**	0.000977	3.40266	0
**d__Bacteria;p__Firmicutes;c__Bacilli;o__Lactobacillales;f__Lactobacillaceae;g__Lactobacillus;s__Lactobacillus_johnsonii;ASV_0067.3v2**	0.009152	3.53223	0
**d__Bacteria;p__Firmicutes;c__Bacilli;o__Lactobacillales;f__Lactobacillaceae;g__Lactobacillus;s__Lactobacillus_johnsonii;ASV_0084.3v2**	0.022494	3.206763	0
**d__Bacteria;p__Firmicutes;c__Bacilli;o__Lactobacillales;f__Lactobacillaceae;g__Lactobacillus;s__Lactobacillus_johnsonii;ASV_0087.3v2**	0.005922	2.880251	0
**d__Bacteria;p__Firmicutes;c__Bacilli;o__Lactobacillales;f__Lactobacillaceae;g__Lactobacillus;s__Lactobacillus_johnsonii;ASV_0102.3v2**	0.014266	2.578198	0
**d__Bacteria;p__Firmicutes;c__Bacilli;o__Lactobacillales;f__Lactobacillaceae;g__Lactobacillus;s__Lactobacillus_johnsonii;ASV_0136.3v2**	0.022494	2.374719	0
**d__Bacteria;p__Firmicutes;c__Bacilli;o__Lactobacillales;f__Lactobacillaceae;g__Lactobacillus;s__Lactobacillus_johnsonii;ASV_0137.3v2**	0.022494	2.898858	0
d__Bacteria;p__Firmicutes;c__Bacilli;o__Lactobacillales;f__Lactobacillaceae;g__Lactobacillus;s__Lactobacillus_murinus;ASV_0001.3v2	0.000977	0	5.451666
d__Bacteria;p__Firmicutes;c__Bacilli;o__Lactobacillales;f__Lactobacillaceae;g__Lactobacillus;s__Lactobacillus_murinus;ASV_0002.3v2	0.001953	3.605705	5.403698
d__Bacteria;p__Firmicutes;c__Bacilli;o__Lactobacillales;f__Lactobacillaceae;g__Lactobacillus;s__Lactobacillus_murinus;ASV_0003.3v2	0.000977	3.003403	5.023143
d__Bacteria;p__Firmicutes;c__Bacilli;o__Lactobacillales;f__Lactobacillaceae;g__Lactobacillus;s__Lactobacillus_murinus;ASV_0004.3v2	0.001953	3.327039	4.916125
d__Bacteria;p__Firmicutes;c__Bacilli;o__Lactobacillales;f__Lactobacillaceae;g__Lactobacillus;s__Lactobacillus_murinus;ASV_0005.3v2	0.000977	3.213733	4.786145
d__Bacteria;p__Firmicutes;c__Bacilli;o__Lactobacillales;f__Lactobacillaceae;g__Lactobacillus;s__Lactobacillus_murinus;ASV_0006.3v2	0.000977	2.964293	4.988954
d__Bacteria;p__Firmicutes;c__Bacilli;o__Lactobacillales;f__Lactobacillaceae;g__Lactobacillus;s__Lactobacillus_murinus;ASV_0008.3v2	0.000977	2.96766	4.435395
d__Bacteria;p__Firmicutes;c__Bacilli;o__Lactobacillales;f__Lactobacillaceae;g__Lactobacillus;s__Lactobacillus_murinus;ASV_0009.3v2	0.000977	0	4.286429
d__Bacteria;p__Firmicutes;c__Bacilli;o__Lactobacillales;f__Lactobacillaceae;g__Lactobacillus;s__Lactobacillus_murinus;ASV_0015.3v2	0.000977	0	4.231002
d__Bacteria;p__Firmicutes;c__Bacilli;o__Lactobacillales;f__Lactobacillaceae;g__Lactobacillus;s__Lactobacillus_murinus;ASV_0017.3v2	0.004883	0	4.04313
d__Bacteria;p__Firmicutes;c__Bacilli;o__Lactobacillales;f__Lactobacillaceae;g__Lactobacillus;s__Lactobacillus_murinus;ASV_0024.3v2	0.000977	0	3.273017
d__Bacteria;p__Firmicutes;c__Bacilli;o__Lactobacillales;f__Lactobacillaceae;g__Lactobacillus;s__Lactobacillus_murinus;ASV_0026.3v2	0.001953	0	4.083468
d__Bacteria;p__Firmicutes;c__Bacilli;o__Lactobacillales;f__Lactobacillaceae;g__Lactobacillus;s__Lactobacillus_murinus;ASV_0027.3v2	0.000977	0	3.427966
d__Bacteria;p__Firmicutes;c__Bacilli;o__Lactobacillales;f__Lactobacillaceae;g__Lactobacillus;s__Lactobacillus_murinus;ASV_0028.3v2	0.000977	0	3.403773
d__Bacteria;p__Firmicutes;c__Bacilli;o__Lactobacillales;f__Lactobacillaceae;g__Lactobacillus;s__Lactobacillus_murinus;ASV_0035.3v2	0.00293	0	3.474899
d__Bacteria;p__Firmicutes;c__Bacilli;o__Lactobacillales;f__Lactobacillaceae;g__Lactobacillus;s__Lactobacillus_murinus;ASV_0036.3v2	0.004883	0	3.614327
d__Bacteria;p__Firmicutes;c__Bacilli;o__Lactobacillales;f__Lactobacillaceae;g__Lactobacillus;s__Lactobacillus_murinus;ASV_0037.3v2	0.00293	0	3.482953
d__Bacteria;p__Firmicutes;c__Bacilli;o__Lactobacillales;f__Lactobacillaceae;g__Lactobacillus;s__Lactobacillus_murinus;ASV_0038.3v2	0.006836	0	3.519235
d__Bacteria;p__Firmicutes;c__Bacilli;o__Lactobacillales;f__Lactobacillaceae;g__Lactobacillus;s__Lactobacillus_murinus;ASV_0040.3v2	0.005922	0	3.779802
d__Bacteria;p__Firmicutes;c__Bacilli;o__Lactobacillales;f__Lactobacillaceae;g__Lactobacillus;s__Lactobacillus_murinus;ASV_0042.3v2	0.001953	0	3.531758
d__Bacteria;p__Firmicutes;c__Bacilli;o__Lactobacillales;f__Lactobacillaceae;g__Lactobacillus;s__Lactobacillus_murinus;ASV_0044.3v2	0.008045	0	2.878056
d__Bacteria;p__Firmicutes;c__Bacilli;o__Lactobacillales;f__Lactobacillaceae;g__Lactobacillus;s__Lactobacillus_murinus;ASV_0046.3v2	0.000977	0	3.470206
d__Bacteria;p__Firmicutes;c__Bacilli;o__Lactobacillales;f__Lactobacillaceae;g__Lactobacillus;s__Lactobacillus_murinus;ASV_0047.3v2	0.000977	0	3.171858
d__Bacteria;p__Firmicutes;c__Bacilli;o__Lactobacillales;f__Lactobacillaceae;g__Lactobacillus;s__Lactobacillus_murinus;ASV_0050.3v2	0.005922	0	3.379715
d__Bacteria;p__Firmicutes;c__Bacilli;o__Lactobacillales;f__Lactobacillaceae;g__Lactobacillus;s__Lactobacillus_murinus;ASV_0051.3v2	0.005922	0	3.408281
d__Bacteria;p__Firmicutes;c__Bacilli;o__Lactobacillales;f__Lactobacillaceae;g__Lactobacillus;s__Lactobacillus_murinus;ASV_0054.3v2	0.001953	0	3.155073
d__Bacteria;p__Firmicutes;c__Bacilli;o__Lactobacillales;f__Lactobacillaceae;g__Lactobacillus;s__Lactobacillus_murinus;ASV_0057.3v2	0.001953	0	3.013141
d__Bacteria;p__Firmicutes;c__Bacilli;o__Lactobacillales;f__Lactobacillaceae;g__Lactobacillus;s__Lactobacillus_murinus;ASV_0058.3v2	0.008045	0	3.043932
d__Bacteria;p__Firmicutes;c__Bacilli;o__Lactobacillales;f__Lactobacillaceae;g__Lactobacillus;s__Lactobacillus_murinus;ASV_0061.3v2	0.005922	0	2.982893
d__Bacteria;p__Firmicutes;c__Bacilli;o__Lactobacillales;f__Lactobacillaceae;g__Lactobacillus;s__Lactobacillus_murinus;ASV_0063.3v2	0.020863	0	3.086216
d__Bacteria;p__Firmicutes;c__Bacilli;o__Lactobacillales;f__Lactobacillaceae;g__Lactobacillus;s__Lactobacillus_murinus;ASV_0064.3v2	0.012851	0	2.279165
d__Bacteria;p__Firmicutes;c__Bacilli;o__Lactobacillales;f__Lactobacillaceae;g__Lactobacillus;s__Lactobacillus_murinus;ASV_0073.3v2	0.008045	0	2.660456
d__Bacteria;p__Firmicutes;c__Bacilli;o__Lactobacillales;f__Lactobacillaceae;g__Lactobacillus;s__Lactobacillus_murinus;ASV_0074.3v2	0.005922	0	2.487842
d__Bacteria;p__Firmicutes;c__Bacilli;o__Lactobacillales;f__Lactobacillaceae;g__Lactobacillus;s__Lactobacillus_murinus;ASV_0075.3v2	0.008045	0	2.700483
d__Bacteria;p__Firmicutes;c__Bacilli;o__Lactobacillales;f__Lactobacillaceae;g__Lactobacillus;s__Lactobacillus_reuteri;ASV_0030.3v2	0.013672	0	3.140048
d__Bacteria;p__Firmicutes;c__Bacilli;o__Lactobacillales;f__Lactobacillaceae;g__Lactobacillus;s__Lactobacillus_reuteri;ASV_0045.3v2	0.019059	0	2.823278
d__Bacteria;p__Firmicutes;c__Bacilli;o__Lactobacillales;f__Lactobacillaceae;g__Lactobacillus;s__Lactobacillus_reuteri;ASV_0066.3v2	0.009152	3.112131	0
d__Bacteria;p__Firmicutes;c__Bacilli;o__Lactobacillales;f__Lactobacillaceae;g__Lactobacillus;s__Lactobacillus_reuteri;ASV_0070.3v2	0.000977	3.12224	0
d__Bacteria;p__Firmicutes;c__Bacilli;o__Lactobacillales;f__Lactobacillaceae;g__Lactobacillus;s__Lactobacillus_reuteri;ASV_0108.3v2	0.022494	2.108985	0
d__Bacteria;p__Firmicutes;c__Bacilli;o__Lactobacillales;f__Lactobacillaceae;g__Lactobacillus;s__Lactobacillus_reuteri;ASV_0114.3v2	0.036032	0	1.599285
d__Bacteria;p__Firmicutes;c__Bacilli;o__Lactobacillales;f__Lactobacillaceae;g__Lactobacillus;s__Lactobacillus_reuteri;ASV_0120.3v2	0.036032	0	2.294372
d__Bacteria;p__Proteobacteria;c__Gammaproteobacteria;o__Burkholderiales;f__Sutterellaceae;g__Parasutterella;ASV_0048.3v2	0.000977	0	3.16881
d__Bacteria;p__Proteobacteria;c__Gammaproteobacteria;o__Burkholderiales;f__Sutterellaceae;g__Parasutterella;ASV_0052.3v2	0.000977	0	3.118163
d__Bacteria;p__Proteobacteria;c__Gammaproteobacteria;o__Burkholderiales;f__Sutterellaceae;g__Parasutterella;ASV_0072.3v2	0.005922	0	2.26349
d__Bacteria;p__Proteobacteria;c__Gammaproteobacteria;o__Burkholderiales;f__Sutterellaceae;g__Parasutterella;ASV_0076.3v2	0.029974	0	2.377127
d__Bacteria;p__Proteobacteria;c__Gammaproteobacteria;o__Burkholderiales;f__Sutterellaceae;g__Parasutterella;ASV_0090.3v2	0.000977	0	2.513248
d__Bacteria;p__Proteobacteria;c__Gammaproteobacteria;o__Burkholderiales;f__Sutterellaceae;g__Parasutterella;ASV_0139.3v2	0.009152	0	2.055587
d__Bacteria;p__Proteobacteria;c__Gammaproteobacteria;o__Burkholderiales;f__Sutterellaceae;g__Parasutterella;ASV_0146.3v2	0.014266	0	1.839102
d__Bacteria;p__Proteobacteria;c__Gammaproteobacteria;o__Burkholderiales;f__Sutterellaceae;g__Parasutterella;ASV_0153.3v2	0.042315	0	1.90133
d__Bacteria;p__Proteobacteria;c__Gammaproteobacteria;o__Burkholderiales;f__Sutterellaceae;g__Parasutterella;ASV_0176.3v2	0.036032	0	1.98
d__Bacteria;p__Proteobacteria;c__Gammaproteobacteria;o__Burkholderiales;f__Sutterellaceae;g__Parasutterella;ASV_0192.3v2	0.022494	0	1.711631
d__Bacteria;p__Proteobacteria;c__Gammaproteobacteria;o__Burkholderiales;f__Sutterellaceae;g__Parasutterella;ASV_0194.3v2	0.036032	0	1.643779
d__Bacteria;p__Proteobacteria;c__Gammaproteobacteria;o__Burkholderiales;f__Sutterellaceae;g__Parasutterella;ASV_0252.3v2	0.036032	0	1.683409
d__Bacteria;p__Proteobacteria;c__Gammaproteobacteria;o__Burkholderiales;f__Sutterellaceae;g__Parasutterella;s__uncultured_organism;ASV_0178.3v2	0.014433	0	1.712543
d__Bacteria;p__Proteobacteria;c__Gammaproteobacteria;o__Burkholderiales;f__Sutterellaceae;g__Parasutterella;s__uncultured_organism;ASV_0341.3v2	0.022494	0	1.086998
d__Bacteria;p__Proteobacteria;c__Gammaproteobacteria;o__Burkholderiales;f__Sutterellaceae;g__Parasutterella;s__uncultured_organism;ASV_0444.3v2	0.014266	0	0.822555
d__Bacteria;p__Proteobacteria;c__Gammaproteobacteria;o__Enterobacterales;f__Enterobacteriaceae;g__Escherichia.Shigella;s__Escherichia_coli;ASV_0092.3v2	0.000977	0	2.014576
d__Bacteria;p__Proteobacteria;c__Gammaproteobacteria;o__Enterobacterales;f__Enterobacteriaceae;g__Escherichia.Shigella;s__Escherichia_coli;ASV_0112.3v2	0.000977	0	2.080336
d__Bacteria;p__Proteobacteria;c__Gammaproteobacteria;o__Enterobacterales;f__Enterobacteriaceae;g__Escherichia.Shigella;s__Escherichia_coli;ASV_0122.3v2	0.005922	0	2.129171
d__Bacteria;p__Proteobacteria;c__Gammaproteobacteria;o__Enterobacterales;f__Enterobacteriaceae;g__Escherichia.Shigella;s__Escherichia_coli;ASV_0132.3v2	0.009152	0	1.952391
d__Bacteria;p__Proteobacteria;c__Gammaproteobacteria;o__Enterobacterales;f__Enterobacteriaceae;g__Escherichia.Shigella;s__Escherichia_coli;ASV_0134.3v2	0.000977	0	2.01501
d__Bacteria;p__Proteobacteria;c__Gammaproteobacteria;o__Enterobacterales;f__Enterobacteriaceae;g__Escherichia.Shigella;s__Escherichia_coli;ASV_0141.3v2	0.000977	0	1.997437
d__Bacteria;p__Proteobacteria;c__Gammaproteobacteria;o__Enterobacterales;f__Enterobacteriaceae;g__Escherichia.Shigella;s__Escherichia_coli;ASV_0164.3v2	0.009152	0	1.6437
d__Bacteria;p__Verrucomicrobiota;c__Verrucomicrobiae;o__Verrucomicrobiales;f__Akkermansiaceae;g__Akkermansia;ASV_0007.3v2	0.000977	1.579203	4.466964
d__Bacteria;p__Verrucomicrobiota;c__Verrucomicrobiae;o__Verrucomicrobiales;f__Akkermansiaceae;g__Akkermansia;ASV_0011.3v2	0.000977	0	4.101451
d__Bacteria;p__Verrucomicrobiota;c__Verrucomicrobiae;o__Verrucomicrobiales;f__Akkermansiaceae;g__Akkermansia;ASV_0012.3v2	0.000977	0	4.150572
d__Bacteria;p__Verrucomicrobiota;c__Verrucomicrobiae;o__Verrucomicrobiales;f__Akkermansiaceae;g__Akkermansia;ASV_0014.3v2	0.000977	0	4.080111
d__Bacteria;p__Verrucomicrobiota;c__Verrucomicrobiae;o__Verrucomicrobiales;f__Akkermansiaceae;g__Akkermansia;ASV_0016.3v2	0.000977	0	4.224386
d__Bacteria;p__Verrucomicrobiota;c__Verrucomicrobiae;o__Verrucomicrobiales;f__Akkermansiaceae;g__Akkermansia;ASV_0020.3v2	0.000977	0	3.714252
d__Bacteria;p__Verrucomicrobiota;c__Verrucomicrobiae;o__Verrucomicrobiales;f__Akkermansiaceae;g__Akkermansia;ASV_0021.3v2	0.000977	0	3.780255
d__Bacteria;p__Verrucomicrobiota;c__Verrucomicrobiae;o__Verrucomicrobiales;f__Akkermansiaceae;g__Akkermansia;ASV_0023.3v2	0.000977	0	3.999043
d__Bacteria;p__Verrucomicrobiota;c__Verrucomicrobiae;o__Verrucomicrobiales;f__Akkermansiaceae;g__Akkermansia;ASV_0033.3v2	0.000977	0	3.381662
d__Bacteria;p__Verrucomicrobiota;c__Verrucomicrobiae;o__Verrucomicrobiales;f__Akkermansiaceae;g__Akkermansia;ASV_0039.3v2	0.000977	0	3.056689
d__Bacteria;p__Verrucomicrobiota;c__Verrucomicrobiae;o__Verrucomicrobiales;f__Akkermansiaceae;g__Akkermansia;ASV_0041.3v2	0.000977	0	3.415431

0.00 5.45

Median of CLR-transformed abundances

Fig. S8. Full-length 16S rRNA gene sequencing by PacBio SMRTbell of ileal bacteria from mice treated with anti-PD-L1 and apyrase associated or not with vancomycin. Significantly different taxa determined through Mann-Whitney test carried out with CLR-transformed bacterial abundances. The blue-white-red heatmap represents the median CLR-transformed abundances of the reported taxonomic units. The taxonomic lineage of each taxon is shown: p, phylum; c, class; o, order; f, family; g, genus; s, species. Taxonomic names written in blue were determined through a manual BLASTN search in GenBank using the sequence of the corresponding Amplicon Sequence Variant (ASV). The bacterial community structure of the ileum was analyzed in anti-PDL1 plus apyrase-treated mice with (n=10) or without (n=10) the administration of vancomycin.

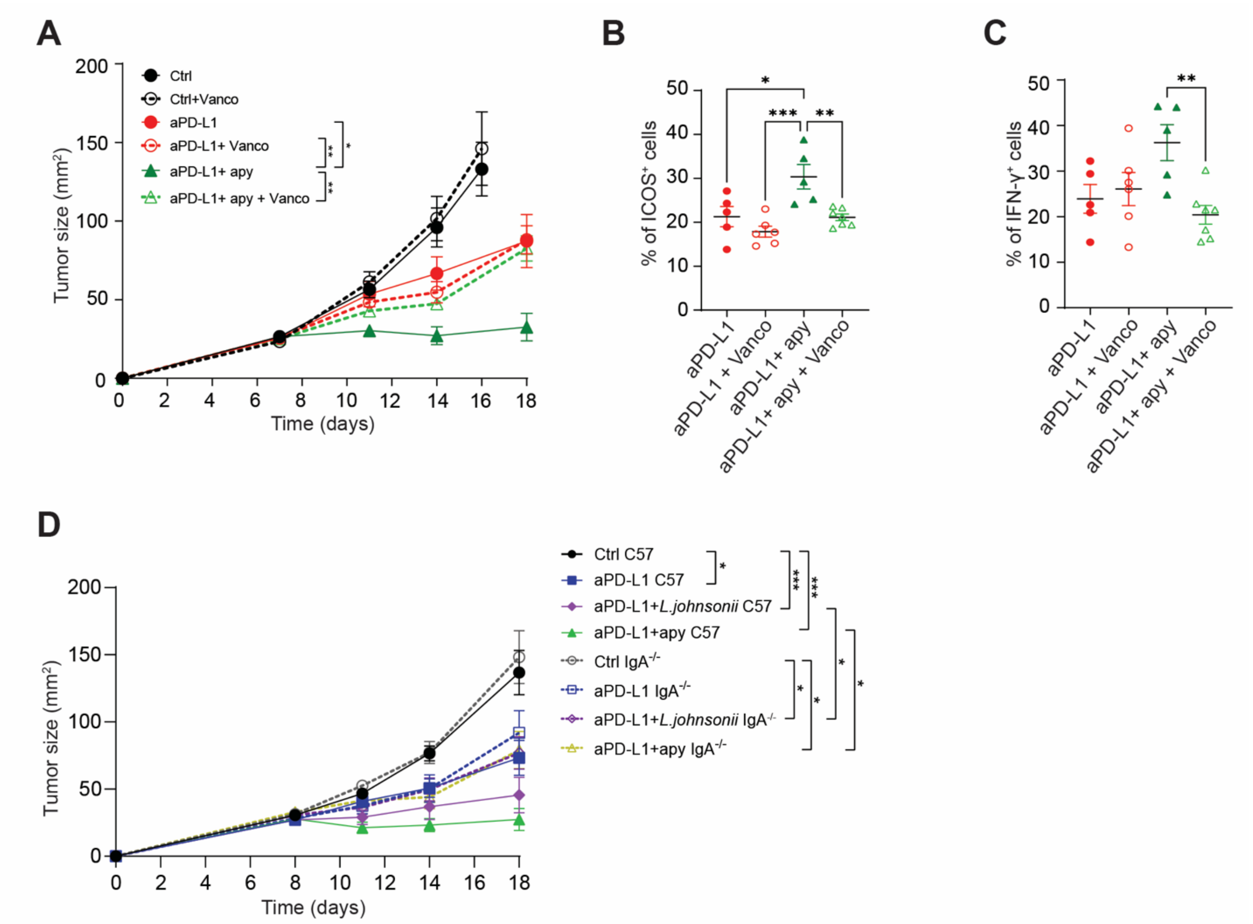

Fig.S9. Effects of vancomycin or *L. johnsonii* administration on IgA-dependent apyrase enhancement of ICB. (**A-C**) Starting from 15 days before tumor inoculation, mice were administered with 200 mg/L of Vancomycin in water ad libitum and kept under vancomycin for the whole duration of the experiment. On day 0 mice were subcutaneously injected with 1 x 10^6 MC38 tumor cells and treated with isotype, standalone anti PD-L1 or combined with 40 µg pure apyrase. (**A**) Tumor growth of mice treated as indicated. Data are presented as the mean ± SEM. Data are from two pooled experiments (n= 8-15 mice/group). Statistical analysis of ICOS (**B**) and IFN-γ (**C**) in $CD8^+$ TILs recovered from tumors at day 18. Data points represent single mice. Data are from two pooled experiments. (**D**) Tumor growth in WT and $IgA^{-/-}$mice treated with PBS or apyrase or *L. johnsonii* in combination with anti-PD-L1. Means ± SEM from two pooled experiments are shown (n= 8-10 mice/group). Two-way ANOVA for tumor growth and One-way ANOVA with Turkey's multiple comparison test were used *p<0.05, **p<0.01, ***p<0.001.

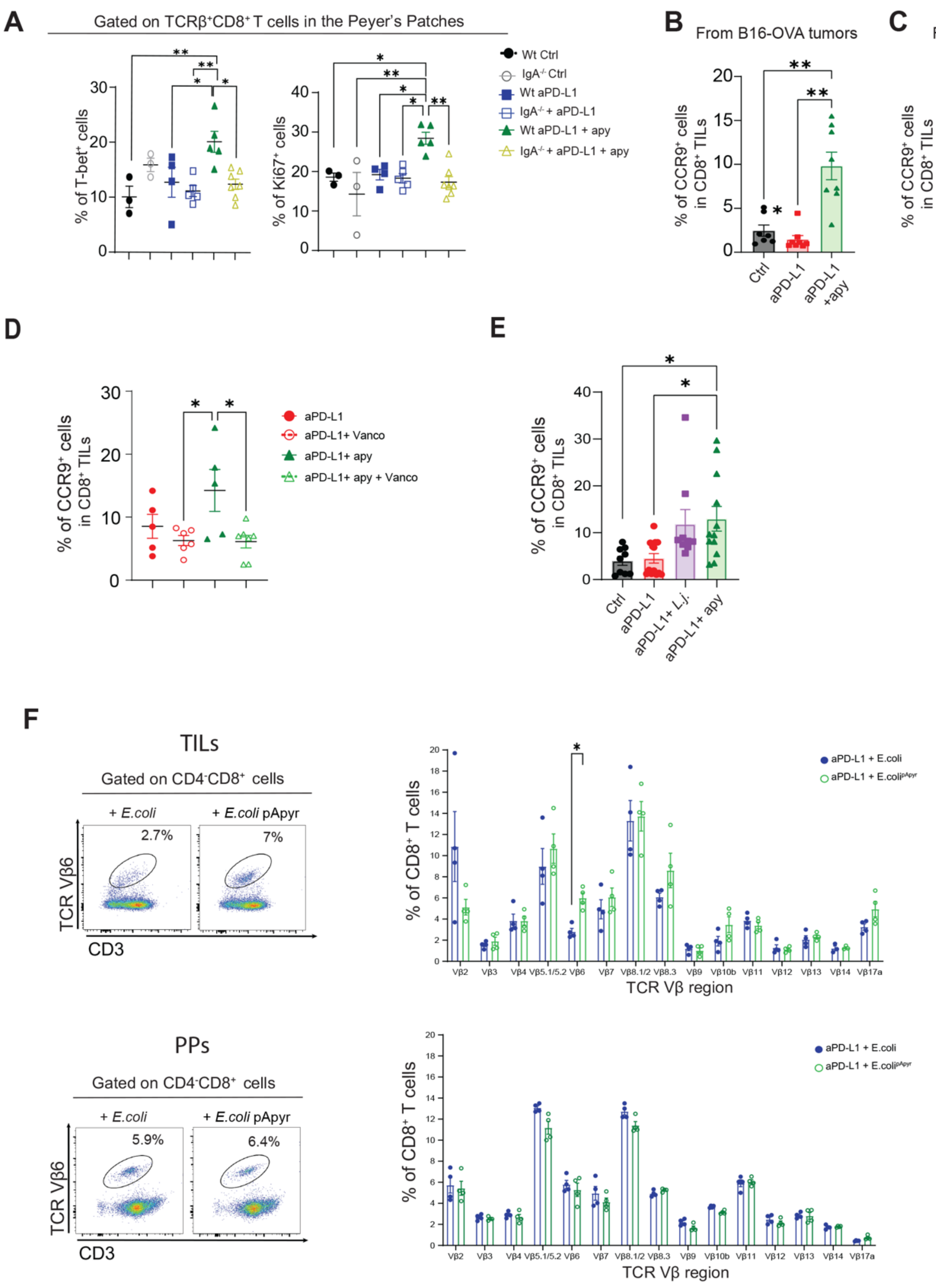

A
Gated on TCRβ+CD8+ T cells in the Peyer's Patches
% of T-bet+ cells
% of Ki67+ cells
Wt Ctrl
IgA-/- Ctrl
Wt aPD-L1
IgA-/- + aPD-L1
Wt aPD-L1 + apy
IgA-/- + aPD-L1 + apy
B
From B16-OVA tumors
% of CCR9+ cells in CD8+ TILs
Ctrl
aPD-L1
aPD-L1 +apy
C
From LLC tumors
% of CCR9+ cells in CD8+ TILs
Ctrl
aPD-L1+aCD40
aPD-L1+aCD40 +apy
D
% of CCR9+ cells in CD8+ TILs
aPD-L1
aPD-L1+ Vanco
aPD-L1+ apy
aPD-L1+ apy + Vanco
E
% of CCR9+ cells in CD8+ TILs
Ctrl
aPD-L1
aPD-L1+ L.j.
aPD-L1+ apy
F
TILs
Gated on CD4-CD8+ cells
+ E.coli
+ E.coli pApyr
2.7%
7%
TCR Vβ6
CD3
% of CD8+ T cells
aPD-L1 + E.coli
aPD-L1 + E.colipApyr
Vβ2 Vβ3 Vβ4 Vβ5.1/5.2 Vβ6 Vβ7 Vβ8.1/2 Vβ8.3 Vβ9 Vβ10b Vβ11 Vβ12 Vβ13 Vβ14 Vβ17a
TCR Vβ region
PPs
Gated on CD4-CD8+ cells
+ E.coli
+ E.coli pApyr
5.9%
6.4%
TCR Vβ6
CD3
% of CD8+ T cells
aPD-L1 + E.coli
aPD-L1 + E.colipApyr
Vβ2 Vβ3 Vβ4 Vβ5.1/5.2 Vβ6 Vβ7 Vβ8.1/2 Vβ8.3 Vβ9 Vβ10b Vβ11 Vβ12 Vβ13 Vβ14 Vβ17a
TCR Vβ region

Fig S10. Functional characterization of intestinal T cells, CCR9 expression in CD8 TILs and TCRVβ repertoire in CD8 TILs and T cells from PPs. (**A**) Bar plots representing the proportions of $Tbet^+$ and $Ki67^+$ cells in CD8 T cells from PPs of WT and $IgA^{-/-}$ mice treated as indicated. **(B**-**E**) Statistical analysis of $CCR9^+$ cells in CD8 TILs at day 18 from subcutaneous engraftment of B16-OVA (**B**) and LLC (**C**) cells in mice treated as indicated, in MC38 tumors from mice treated with anti-PD-L1 or anti-PD-L1 with apyrase in the presence or not of vancomycin (**D**) and mice gavaged with apyrase or *L .johnsonii* (**E**). Displayed data are means ± SEM. (**F**) Representative flow cytometry dot plots and statistical analysis of cells expressing different TCRVβs among CD8 TILs (upper panel) or CD8 T cells from PPs (lower panel). Every dot represents 5 mice from different experiments. The Anti-Mouse TCRVβ Screening Panel (BD Biosciences) was used. One-way ANOVA with Turkey's multiple comparison. *p<0.05, **p<0.01, ***p<0.00

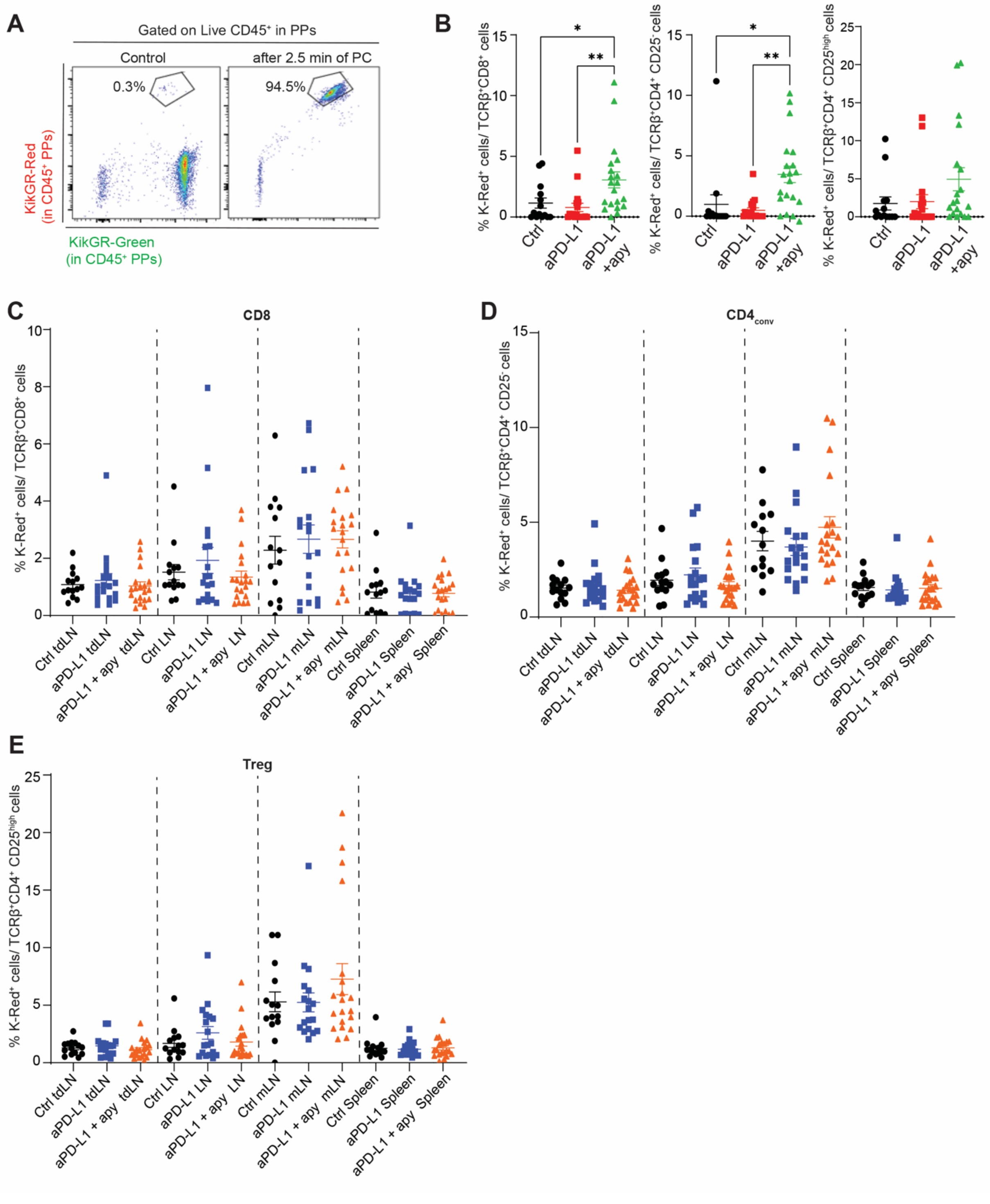
A
Gated on Live CD45+ in PPs
Control
after 2.5 min of PC
0.3%
94.5%
KikGR-Red (in CD45+ PPs)
KikGR-Green (in CD45+ PPs)
B
% K-Red+ cells/ TCRβ+CD8+ cells
% K-Red+ cells/ TCRβ+CD4+ CD25- cells
% K-Red+ cells/ TCRβ+CD4+ CD25high cells
Ctrl
aPD-L1
aPD-L1 +apy
C
CD8
% K-Red+ cells/ TCRβ+CD8+ cells
D
CD4conv
% K-Red+ cells/ TCRβ+CD4+ CD25- cells
E
Treg
% K-Red+ cells/ TCRβ+CD4+ CD25high cells
Ctrl tdLN
aPD-L1 tdLN
aPD-L1 + apy tdLN
Ctrl LN
aPD-L1 LN
aPD-L1 + apy LN
Ctrl mLN
aPD-L1 mLN
aPD-L1 + apy mLN
Ctrl Spleen
aPD-L1 Spleen
aPD-L1 + apy Spleen

Fig. S11. Analysis of gut-derived T cells in lymphoid organs from mice treated with anti-PD-L1 and apyrase. (**A**) Flow cytometry dot-plots showing $CD45^+$ cells from non-photoconverted PPs (Control) or isolated from the PPs 2.5 min after photoconversion. (**B**) Percentage of $K\text{-}Red^+$ cells within parental $CD8^+$ T cells, $CD25^{neg}$ Tconv or $CD25^{hi}$ Tregs in the TME 24 h post-photoconversion. Data points represent single mice. Three experiments were pooled together. Error bars represent SEM. (**C-E**) Percentage of $K\text{-}Red^+$ cells within the same T cell subsets isolated from the tumor-draining lymph node, controlateral lymph node, mesenteric lymph nodes or spleen 24 h post-photoconversion. Data points represent single mice. Three experiments pooled together are shown. Error bars represent SEM. One-way ANOVA with Turkey's multiple comparison test was used. $*p < 0.05$, $**p < 0.01$.

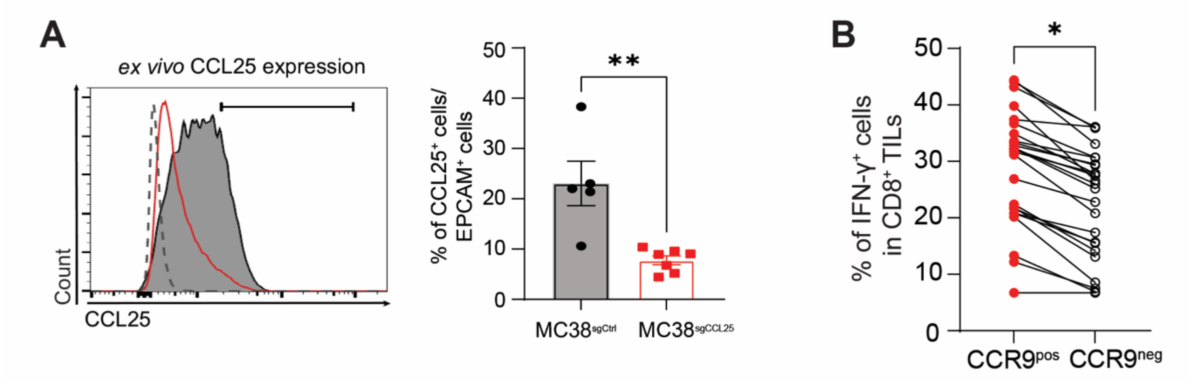

Fig. S12. Down-regulation of CCL25 in *ex vivo* MC38^{sgCCL25} cells and IFN-γ expression in CD8^{+} TILs either expressing or not CCR9. (**A**) Representative histogram (left) and statistical analysis (right) of CCL25 expression in ex vivo isolated MC38 cells. Tumors were explanted after 15 days from subcutaneous injection and cells were selected as Zombie^{-}, CD45^{-} and EPCAM^{+}. Dotted line shows the staining of MC38^{sgCtrl} cells with the secondary antibody, whereas red empty line represents the staining of MC38 cells transduced with sg^{CCL25} and the black full line represents MC38 cells transduced with sg^{Ctrl} stained with anti-CCL25. Two experiments pooled together are shown. (**B**) Percentage of IFN-γ secreting cells among CCR9^{+} or CCR9^{-} CD8 TILs. Two experiments pooled together are shown. Error bars represent SEMs. Two-tailed Mann-Whitney U test was used $^{*}p < 0.05$; $^{**}p < 0.01$.

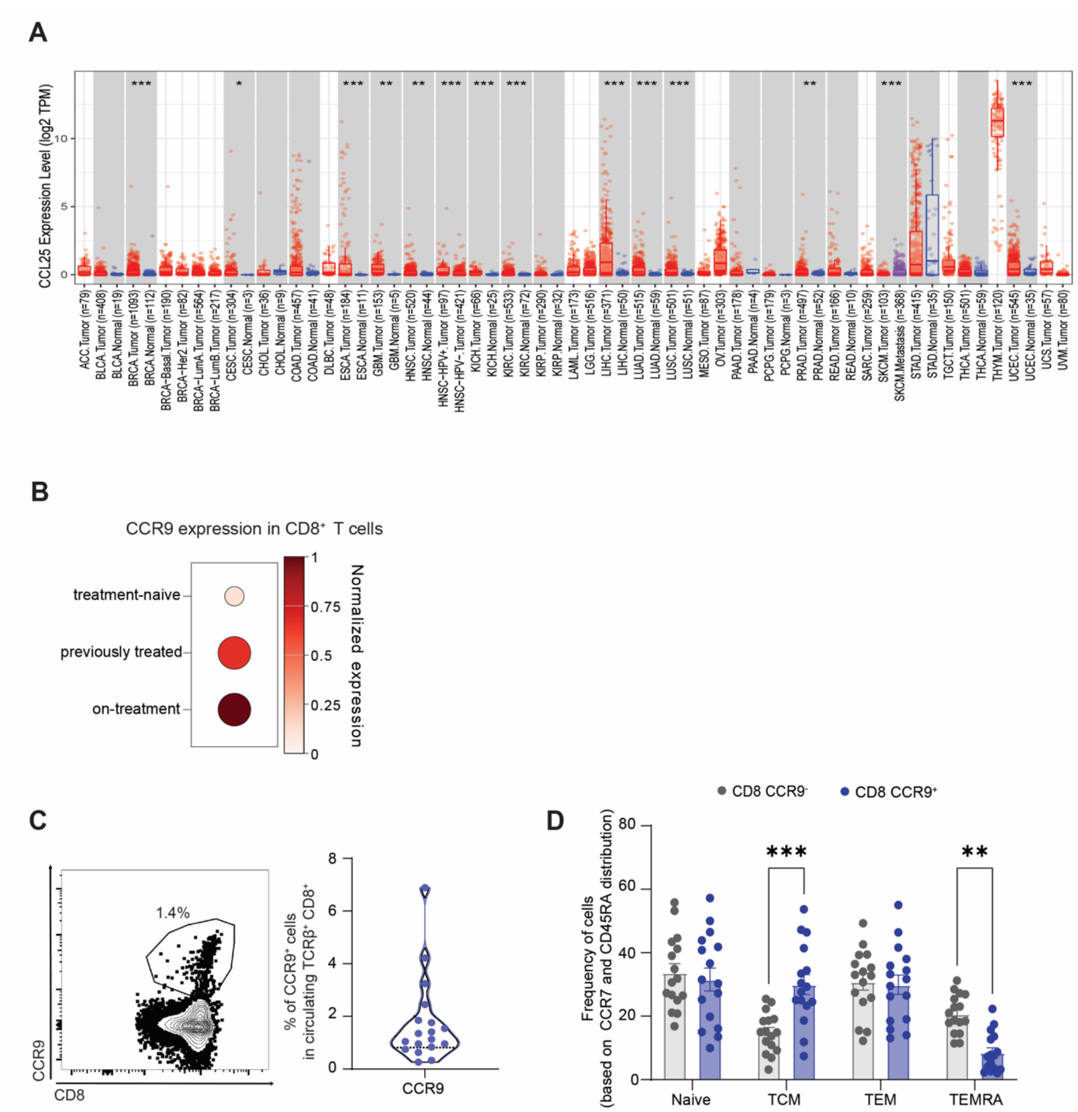

Fig. S13. CCL25 expression in different tumors and phenotype of human circulating CCR9⁺ cells. (**A**) Differential expression between tumor and adjacent normal tissues of *CCL25* across TCGA tumors. Distributions of gene expression levels are displayed using box plots. Statistical differences were determined by Wilcoxon matched-pairs signed rank test; $*p < 0.05$, $**p < 0.01$, $***p < 0.001$ (http://timer.cistrome.org/). (**B**) Dot plot showing the normalized expression of CCR9 in $CD8^+$ T cells isolated from ICB-naïve melanoma patients, patients that had been previously treated, and those on treatment. (**C**) Representative dot plot and graphical visualization of the percentage of circulating $CCR9^+$ cells gated on $CD3^+CD8^+$ cells in the PBMCs of healthy individuals. (**D**) Statistical analysis of $CCR9^+$ vs $CCR9^-$ circulating CD8 T cells for Naïve, T central memory (Tcm), T effector memory (Tem) and Terminally differentiated effector (Temra) cells. Every dot represents a single individual. Error bars represent SEM. One-way ANOVA with Turkey's multiple comparison test was used. $**p < 0.01$, $***p < 0.001$.

Table S1. RNASeq of epithelial cells

Table S2. GSEA Reactome of epithelial cells

Table S3. ASV assignment of IgA^{+} bacteria

Table S4. CCL25 expression in melanoma patients
